# Deep sequencing of *Brachypodium *small RNAs at the global genome level identifies microRNAs involved in cold stress response

**DOI:** 10.1186/1471-2164-10-449

**Published:** 2009-09-23

**Authors:** Jingyu Zhang, Yunyuan Xu, Qing Huan, Kang Chong

**Affiliations:** 1 Key Laboratory of Photosynthesis and Environmental Molecular Physiology, Chinese Academy of Sciences, and National Centre for Plant Gene Research, Beijing 100093, PR China

## Abstract

**Background:**

MicroRNAs (miRNAs) are endogenous small RNAs having large-scale regulatory effects on plant development and stress responses. Extensive studies of miRNAs have only been performed in a few model plants. Although miRNAs are proved to be involved in plant cold stress responses, little is known for winter-habit monocots. *Brachypodium distachyon*, with close evolutionary relationship to cool-season cereals, has recently emerged as a novel model plant. There are few reports of *Brachypodium *miRNAs.

**Results:**

High-throughput sequencing and whole-genome-wide data mining led to the identification of 27 conserved miRNAs, as well as 129 predicted miRNAs in *Brachypodium*. For multiple-member conserved miRNA families, their sizes in *Brachypodium *were much smaller than those in rice and *Populus*. The genome organization of miR395 family in *Brachypodium *was quite different from that in rice. The expression of 3 conserved miRNAs and 25 predicted miRNAs showed significant changes in response to cold stress. Among these miRNAs, some were cold-induced and some were cold-suppressed, but all the conserved miRNAs were up-regulated under cold stress condition.

**Conclusion:**

Our results suggest that *Brachypodium *miRNAs are composed of a set of conserved miRNAs and a large proportion of non-conserved miRNAs with low expression levels. Both kinds of miRNAs were involved in cold stress response, but all the conserved miRNAs were up-regulated, implying an important role for cold-induced miRNAs. The different size and genome organization of miRNA families in *Brachypodium *and rice suggest that the frequency of duplication events or the selection pressure on duplicated miRNAs are different between these two closely related plant species.

## Background

MicroRNAs (miRNAs) are a class of small non-protein-coding RNAs generated from single-stranded precursors with unique hairpin structures. They regulate the expression of mRNAs by targeting transcripts for cleavage or translational repression [1]. The miRNAs were initially isolated in *Caenorhabditis elegans *as developmental timing regulators [2]. Since then, they have been found in a broad range of plants, as well as viruses and mammals. Plants miRNAs were first identified in *Arabidopsis *by different research groups [3-5]. The biogenesis of plant miRNAs is a complex multi-step enzymatic process [6-8]. The miRNAs are initially transcribed by RNA polymerase II in the cell nucleus as long primary miRNAs that are cleaved into the miRNA:miRNA* duplexes by Dicer-like 1 (DCL1). Export of the duplexes into the cell cytoplasm is mediated by HASTY. After methyl groups are added to the 3' ends of the duplexes catalyzed by HEN1, one strand of the duplexes is selectively incorporated into the RNA-induced silencing complex (RISC) to form the mature miRNAs, whereas the other strand, designated the miRNA*, is typically degraded. DCL4 has also been shown to play a role in the biogenesis of a few miRNAs with long hairpin precursors [9]. Plant miRNAs exhibit high complementarity to their targets and can direct RISC-mediated cleavage of target mRNAs. Thus, it is widely accepted that plant miRNAs induce post-transcriptional gene silencing predominantly through guiding mRNA cleavage. Recent studies, however, have showed that translational inhibition is another kind of action mechanism for miRNAs in plants [10-13].

Although initial studies have largely demonstrated the role of plant miRNAs in development and morphogenesis processes, there are increasing number of reports indicating that plant miRNAs also target genes involved in biotic as well as abiotic stress responses [1,14,15]. Low temperature is one of the most important environmental stimuli that affect plant growth and development. Recently, more and more reports demonstrate the role of plant miRNAs in cold stress response. The first study in this area, performed by Sunkar and Zhu, revealed the induced expression of miR393, miR397b and miR402 in response to cold stress as well as other kinds of stress treatment. In this study, miR319c appeared to be up-regulated by cold but not by dehydration, NaCl, or ABA treatment [16]. Zhou *et al*. (2008) identified cold up-regulated miRNAs using a computational, transcriptome-based approach [17]. Liu *et al*. (2008) and Lu *et al*. (2008) used microarray analysis to identify cold-responsive miRNAs in *Arabidopsis *and *Populus*, respectively [18,19]. These studies indicate that the expression of several miRNAs is affected by the cold treatment. Despite these efforts, our knowledge of the role played by miRNAs in plant cold stress response is still limited at the whole-genome level.

The most challenging problem in understanding plant miRNAs is to identify more novel miRNAs. Three major approaches have been used for miRNA discovery in plants: forward genetics, bioinformatic prediction as well as direct cloning and sequencing. Only a few miRNAs were identified by forward genetic studies [10,20-22] and predicting species-specific miRNAs using bioinformatics method is difficult. Thus, direct cloning and sequencing is the most effective method for plant miRNA discovery. Only a few hundred miRNAs have been identified with this approach, which leads to a premature conclusion that the types of miRNAs in plants are limited. Recently, the deep sequencing approach appeared and allowed the identification of numerous small RNAs [9,23-27]. It not only revealed a lot more species-specific miRNAs, but also provided a picture of the genomic landscape of small RNAs.

Although significant progress has been made in identifying plant miRNAs and understanding their action mechanism, the discovery of novel miRNAs in plants on a genome-wide scale is still at the preliminary stage. One limiting factor in miRNA discovery is the availability of the whole genome sequence, only with which a comprehensive analysis of the potential hairpin precursor structures of cloned small RNAs can be performed to distinguish miRNAs from other kinds of small RNAs. Thus, most of the studies have been done in *Arabidopsis*, rice and *Populus*, whose whole genome sequences are known. Many evolutionarily or economically important species have not been examined yet. To further understand the function of plant miRNAs, more effort should be directed toward plant species with specific developmental features, which may contain miRNAs that are specific for these features [27]. Several economically important winter-habit monocots, such as winter wheat, barley, oat, rye, as well as cool-season biofuel and forage grasses, can cold-acclimate and acquire high tolerance to low temperature. Although it has been shown that miRNAs are involved in plant cold stress response, little work has been done for monocotyledonous plant, especially for these winter-habit monocots, probably because rice, the model plant for monocots, is a tropical plant and incapable of cold acclimation.

*Brachypodium distachyon *has emerged as a new monocot model plant, especially for temperate cereals and related grasses [28,29]. *Brachypodium *not only has a closer evolutionary relationship with cool-season temperate cereals and grasses than rice, but also possesses growth and developmental features that are common to these plants. As a widely distributed winter-habit temperate plant, it has vernalization requirement and is capable of cold acclimation [30,31]. Recently, the draft genome sequence of *Brachypodium *has been released , which makes this plant a good model for performing whole-genome-wide study of miRNAs involved in cold response of winter-habit temperate cereals and grasses. Here we sequenced small RNA populations in *Brachypodium *with and without cold stress treatment using Solexa, the high-throughput sequencing technology. Our studies not only identified species-specific miRNAs for the novel monocotyledonous model plant, but also provided useful information for cold-responsive miRNAs in temperate cereals and grasses.

## Results

### Deep sequencing of *Brachypodium *small RNAs

Two small RNA libraries, with (WC) and without (NC) cold-treatment, were generated using pooled RNA isolated from the aerial parts of *Brachypodium *seedlings. Sequencing of the *Brachypodium *small RNA libraries was performed with Solexa, a high throughput sequencing technology producing highly accurate, reproducible and quantitative readouts of small RNAs [32,33]. It can be used as a tool for miRNA expression profiling [24,26,34]. For direct comparison, *Brachypodium *seedlings used for NC and WC library construction were grown under similar conditions except for the cold treatment. The same amount of RNA was used to construct these two libraries and the samples were prepared in a similar manner. Sequencing of these two libraries was performed on the Illumina's Solexa Sequencer and the samples were run side by side.

**Figure 1:**
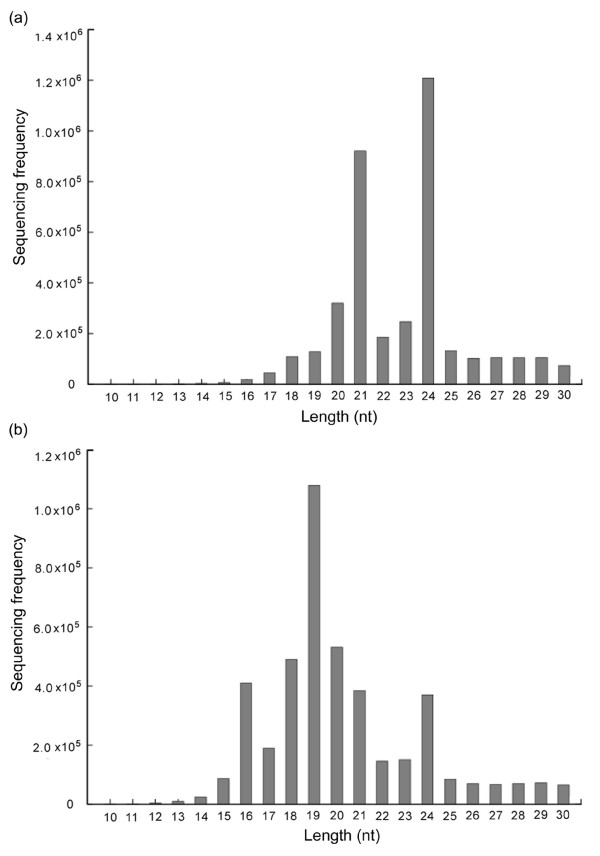
**The size distribution of *Brachypodium *small RNAs in NC (a) and WC (b) libraries**.

Solexa sequencing of NC and WC libraries generated a total of 5,339,385 and 5,452,742 raw reads, respectively. Analysis of these reads resulted in identification of 3,741,194 and 3,585,498 sequences ranging from 18 to 30 nucleotides (nt), respectively (Table 1). The remaining sequences were either of low quality (readings without reliable 3' adaptor sequence) or smaller than 18 nt, and were excluded from further analysis. The majority of the obtained small RNA sequences from the NC library were 20-24 nt in size, which is the typical size range for Dicer-derived products (Figure 1a). These small RNAs were used for further analysis.

**Table 1:** Statistics of small RNA sequences from the *Brachypodium *NC and WC libraries.

	**Sequences generated**	**Unique sequences**
NC library		
Raw reads	5,339,385	
Low quality reads removed	4,702,002	
Adaptors removed	3,816,932	
Sequences < 18 nt removed	3,741,194	
Sequences matching *Brachypodium *draft genome	2,603,872	
Non-coding RNA exact matches removed	2,248,474	711,207
Match known miRNAs	851,945	27
Genome-matched small RNAs with predicted hairpin structures	41,155	235
WC library		
Raw reads	5,452,742	
Low quality reads removed	4,720,226	
Adaptors removed	4,314,516	
Sequences < 18 nt removed	3,585,498	
Sequences matching *Brachypodium *draft genome	2,958,093	
Non-coding RNA exact matches removed	1,264,919	423,463
Match known miRNAs	337,863	27
Genome-matched small RNAs with predicted hairpin structures	12,750	218

The 20-24 nt sequences from the NC and WC libraries were aligned to the draft *Brachypodium *genome using SOAP [35], and a total of 2,603,872 (NC) and 2,958,093 (WC) sequences were found to match the genome perfectly. After removing the sequences corresponding to known rRNAs, tRNAs, small nuclear RNAs and small nucleolar RNAs, a total of 2,248,474 (NC) and 1,264,919 (WC) sequences were obtained (Table 1). The WC library contained more non-coding RNAs than the NC library, which reduced the population size of non-coding-RNA-removed genome-matched sequences in the WC library to half of that in the NC library. For direct comparison, the NC library was normalized to the WC library according to the population size mentioned above. In both NC and WC libraries, most of the known miRNAs and predicted miRNAs were detected at least 3 times, enabling the comparison of miRNA expression levels between these two libraries.

### Identification of novel monocot-specific and *Brachypodium*-specific miRNAs

One important feature that distinguishes miRNAs from other endogenous small RNAs is that their surrounding sequences can adopt a hairpin structure [36,37]. The raw whole genome sequence data of *Brachypodium distachyon *has been released by the US Department of Energy Joint Genome Institute ;  and gene annotation was accomplished with FGENESH. In this study, the annotated *Brachypodium *genome sequences were used to predict hairpin structures based on miRNA surrounding sequences. To identify miRNAs in *Brachypodium*, the following strategy was adopted. First, the cloned sequences were aligned with the *Brachypodium *genome sequence using SOAP [35] to search for perfectly matched sequences. In the second step, sequences corresponding to known non-coding RNAs (rRNAs, tRNAs, small nuclear and small nucleolar RNAs) were discarded by performing the BLASTn search [38] against the GenBank  and Rfam databases [39]. The remaining sequences were used for the fold-back structure prediction and classified as either miRNA candidates or other kinds of endogenous small RNAs.

Our analysis revealed that in the NC library 2,248,474 sequences can be used for fold-back structure prediction and those that fulfilled the hairpin structure criteria described by Jones-Rhoades *et al*. [1] were selected as candidate miRNAs (Table 1). Among these candidates, some also matched the plastid or mitochondrial genomes. These sequences were not considered as miRNAs in this study. After these analyses, 235 unique sequences were obtained as miRNA candidates (Table 1). According to the currently available gene annotation of the *Brachypodium *genome , 43 of these miRNA candidates were located in the exons of potentially protein-coding genes, which were not further analyzed. Among the remaining 192 *Brachypodium *miRNA candidates, 63 of them have really low sequencing reads and were not detected in the WC library. Those candidates were excluded from further research according to the updated plant miRNA annotation criteria [40]. The remaining 129 putative *Brachypodium *miRNAs were carefully examined to be sure that they are qualified for the updated plant miRNA annotation criteria [40].

The lengths of the 129 predicted *Brachypodium *miRNAs vary from 20 to 22 nt, and more than half of them begin with 5' uridine (Additional file 1, Additional file 2), which is a characteristic feature of miRNAs [4,41,42]. The minimum free energies of these miRNA precursors range from -37.50 to -158.20 kcal mol^-1^, and the average value is about -73.73 kcal mol^-1 ^(Additional file 1), which is similar to the free energy values of miRNA precursors in rice and *Arabidopsis *[43]. These values are much lower than the folding free energies of tRNAs (-27.5 kcal mol^-1^) or rRNAs (-33 kcal mo^-1^) [44]. The predicted hairpin structures for the miRNA precursors are in the range of 71-265 nt (Additional file 1), which is similar to what was observed in rice [25]. None of these putative miRNAs has been found in *Arabidopsis *or rice before.

### Identification of conserved miRNAs in *Brachypodium*

To identify the conserved miRNA homologs in *Brachypodium*, small RNA libraries were analyzed for the presence of known miRNAs. BLASTn search [38] with an E-value cutoff of 10 was employed to search for small RNAs with predicted hairpin structures against the central miRNA Registry Database (miRBase, ). With this similarity search, 27 unique sequences were identified as known miRNAs. They showed higher sequence similarity to their homologs in rice than in *Arabidopsis *and *Populus *(Table 2). All of these conserved miRNA precursors were identified in the draft *Brachypodium *genome and they can adopt hairpin structures (Additional file 3). Based on sequence similarity, these known miRNAs could be grouped into 21 families (Table 2). Four conserved miRNA families were represented by more than one sequence variant in the small RNA libraries. MiR164, miR166 and miR172 were represented by two variants and miR169 was represented by four variants in the library (Table 2). Some of these miRNAs including miR156, miR160, miR166 and mi171 are deeply conserved, even in lower plants such as *Physcomitrella patens *[45]. MiR528 and miR529, which are species-specific miRNAs, were also found in the NC library. According to miRBase  and published papers, miR528 only appears in *Oryza sativa *and miR529 has been identified in *Eschscholzia californica *[46], *Oryza sativa, Physcomitrella patens *as well as some *Pinus *species [46]. The miRNA* sequences were detected for some bdi-miRNAs (Table 2, Additional file 3). The isolation frequencies for most of these miRNA* sequences were about one-tenth of those for mature miRNAs, consistent with the conclusion of Rajagopalan *et al*. [9]. For miR529, there were 631 reads of the miRNA and 17 reads of the miRNA* in the NC library. Similarly, in the WC library miR529 had 268 reads of the miRNA and 11 reads of the miRNA*. The miR529* was reported to be sequenced more often than the miR529 in rice and the annotated miR529*, therefore, is suspected to be the true miRNA [26]. Our data indicate that miR529 is the true miRNA and the existing annotation should not be changed. It is also interesting to note that the miR169f was sequenced 140 times in the NC library and its potential miRNA* was sequenced 103 times, which is almost equal to the mature miRNA sequencing frequency.

**Table 2:** Conserved *Brachypodium *miRNAs and their sequence similarity to known miRNAs from other plant species.

**Name**	**Sequence (5'-3')**	**L** **(nt)**	**Arm** **/nt**	**Location**	**Plant species**			
					**Ara**	**Pop**	**Ric**	**Whe**
bdi-miR156a	UGACAGAAGAGAGUGAGCAC	20	5'/123	super_2:28299658-28299677	++++	++++	++++	++++
bdi-miR156b			5'/123	super_2:28299895-28299914				
bdi-miR156c			5'/152	super_2:28300123-28300142				
bdi-miR156d			5'/112	super_5:10043111-10043130				
bdi-miR156e			5'/112	super_6:14734765-14734784				
bdi-miR156f			5'/186	super_8:656857-656876				
bdi-miR156g			5'/124	super_10:7252868-7252887				
bdi-miR160a	UGCCUGGCUCCCUGUAUGCCA	21	5'/116	super_0:10869231-10869251	++++	++++	++++	++++
bdi-miR160b			5'/126	super_0:34912728-34912748				
bdi-miR160c			5'/129	super_3:8233739-8233759				
bdi-miR160d			5'/121	super_6:15675857-15675877				
bdi-miR162	UCGAUAAACCUCUGCAUCCGG	21	3'/133	super_5:10427165-10427185	+++	+++	+++	
bdi-miR164a	UGGAGAAGCAGGGCACGUGCA	21	5'/186	super_0:24670885-24670905	++++	++++	++++	+++
bdi-miR164b			5'/127	super_2:4986204-4986224				
bdi-miR164c			5'/161	super_2:12019567-12019587				
bdi-miR164d	UGGAGAAGAAGGGCACAUGCA	21	5'/165	super_2:12019673-12019693	+	++	+	
bdi-miR166a	UCGGACCAGGCUUCAUUCCCC	21	3'/222	super_0:8156180-8156200	++++	++++	++++	
bdi-miR166b			3'/119	super_0:32809584-32809604				
bdi-miR166c			3'/108	super_1:32626519-32626539				
bdi-miR166d			3'/121	super_5:8578628-8578648				
bdi-miR166e			3'/148	super_8:6993194-6993214				
bdi-miR166f			3'/137	super_8:12758179-12758199				
bdi-miR166g	UCGGACCAGGCUUCAUUCCUC	21	3'/115	super_12:6251912-6251932	+++	+++	++++	++
bdi-miR167a	UGAAGCUGCCAGCAUGAUCUA	21	5'/127	super_0:33041618-33041638	++++	++++	++++	++++
bdi-miR167b			5'/165	super_0:35725085-35725105				
bdi-miR167c			5'/122	super_12:1692366-1692386				
bdi-miR168	UCGCUUGGUGCAGAUCGGGAC	21	5'/101	super_6:17349865-17349885	++	++	++++	++++
bdi-miR169a	AGCCAAGGAUGACUUGCCGG	20	5'/138	super_0:11730514-11730533	+++	++	+++	+++
bdi-miR169b			5'/117	super_5:1266552-1266571				
bdi-miR169c	UAGCCAAGGAUGACUUGCCUG	21	5'/129	super_5:16644104-16644124	++++	++	++++	
bdi-miR169d			5'/138	super_5:16647089-16647109				
bdi-miR169e	CAGCCAAGAAUGGCUUGCCUA	21	5'/112	super_7:4154045-4154066	+	+	+	+
bdi-miR169f	CAGCCAAGGAUGACUUGCCGG	21	5'/125	super_9:4769323-4769343	++++	+++	++++	++++
bdi-miR171a	UGAUUGAGCCGUGCCAAUAUC	21	3'/102	super_0:7984997-7985017	+	+	++++	+
bdi-miR171b			3'/132	super_0:32458278-32458298				
bdi-miR171c			3'/130	super_1:34018204-34018224				
bdi-miR171d			3'/125	super_9:5726634-5726654				
bdi-miR172a	AGAAUCUUGAUGAUGCUGCAU	21	3'/123	super_5:4196588-4196608	+++	+++	+++	+++
bdi-miR172b	GAAUCUUGAUGAUGCUGCAU	20	3'/226	super_13:3713167-3713186	++++	++++	++++	++++
bdi-miR319	UUGGACUGAAGGGUGCUCCCU	21	3'/190	super_4:17219328-17219348	+++	++	+++	+++
bdi-miR390	AAGCUCAGGAGGGAUAGCGCC	21	5'/186	super_0:36774796-36774816	++++	++++	++++	++++
bdi-miR393	UCCAAAGGGAUCGCAUUGAUC	21	5'/125	super_9:7153943-7153963	+++	++++	++++	++++
bdi-miR394	UUGGCAUUCUGUCCACCUCC	20	5'/172	super_5:7680773-7680792	++++	++++	++++	
bdi-miR395a	UGAAGUGUUUGGGGGAACUC	20	3'/90	super_1:16273497-16273476	+++	+	++	
bdi-miR395b			3'/89	super_1:16273342-16273323				
bdi-miR395c			3'/95	super_1:16272920-16272901				
bdi-miR395d			3'/143	super_6:4370549-4370568				
bdi-miR395e			3'/150	super_9:6484473-6484492				
bdi-miR395f			3'/96	super_9:6484650-6484669				
bdi-miR395g			3'/89	super_9:6484944-6484963				
bdi-miR395h			3'/76	super_9:6485096-6485115				
bdi-miR395i			3'/86	super_9:6485234-6485253				
bdi-miR395j			3'/88	super_9:6485371-6485390				
bdi-miR395k			3'/86	super_9:6485509-6485528				
bdi-miR395l			3'/96	super_9:6485786-6485805				
bdi-miR395m			3'/96	super_9:6486060-6486079				
bdi-miR395n			3'/90	super_9:6486197-6486216				
bdi-miR396a	UCCACAGGCUUUCUUGAACUG	21	5'/161	super_0:1364119-1364139	++	+	++++	
bdi-miR396b			5'/176	super_5:545413-545433				
bdi-miR397a	AUUGAGUGCAGCGUUGAUGAA	21	5'/115	super_6:15909808-15909828	+	+	+	+++
bdi-miR397b			5'/112	super_6:15937103-15937123				
bdi-miR398	UGUGUUCUCAGGUCGCCCCUG	21	3'/131	super_4:6919237-6919257	+++	++++	++++	
bdi-miR528	UGGAAGGGGCAUGCAGAGGAG	21	5'/119	super_1:34315939-34315959			++++	
bdi-miR529	AGAAGAGAGAGAGUACAGCCU	21	5'/107	super_5:15162755-15162775		+++	+++	
bdi-miR827	UUAGAUGACCAUCAGCAAACA	21	3'/141	super_10:7087762-7087782	++	++	+	

Name, the name of conserved *Brachypodium *miRNAs and underlines denote miRNAs whose miRNA* have been detected in Solexa sequencing analysis; Sequence, miRNA sequence cloned in the NC library and; L, the length of miRNA; Arm/nt, the miRNA location in the predicted hairpin structure (5' or 3' arm)/the length of the precursor; N, the number of loci in the genome for different miRNA families; Location, miRNA location in the genome; Ara, *Arabidopsis*; Pop, *Populus*; Ric, Rice; Whe, Wheat; For plus symbols in the table: ++++, miRNA sequences of *Brachypodium *were identical to those in other plant species; +++, miRNA sequences of *Brachypodium *were conserved in other plant species but have variations at 1 nucleotide positions; ++, miRNA sequences of *Brachypodium *were conserved in other plant species but have variations at 2 nucleotide positions; +, miRNA sequences of *Brachypodium *were conserved in other plant species but have variations at ≥ 3 nucleotide positions. For miRNAs whose homologs are not identified in rice, sequence comparison was only performed for the other four plant species.

**Figure 2:**
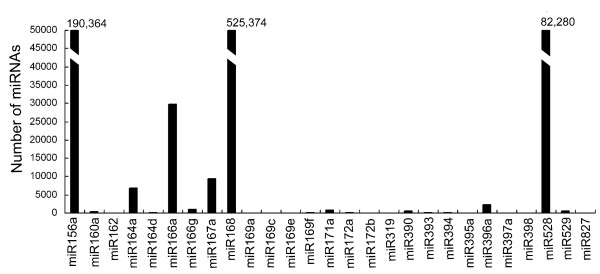
**Abundance of conserved miRNAs sequences in *Brachypodium *small RNA libraries**. The sequencing frequency of conserved miRNAs in the NC library. The sequencing frequency of miR156a, miR168 and miR528 are too high to be indicated in the figure and are shown as numbers on the top of bars.

The sequencing frequencies for miRNAs in the library could be used as an index for estimating the relative abundance of miRNAs. Solexa sequencing produced a large number of miRNA sequences, allowing us to determine the relative abundance of miRNAs in *Brachypodium*. The frequencies of the miRNAs varied from 4 (miR398) to 525,374 (miR168), indicating that the expression of miRNAs varied greatly in *Brachypodium *(Figure 2). The miR168 was the most abundant miRNA in our two sequencing datasets, accounting for about 20% of all sequence reads matching with the *Brachypodium *genome and about 60% of the sequence reads of the conserved miRNAs (Table 1, Figure 2). MiR168 has also been the most abundant miRNA in previous high-throughput sequencing studies in rice [47,48], but not in *Arabidopsis *.

According to the sequencing frequencies, some miRNAs (miR156a, miR164a, miR166a, miR166g, miR167a, miR168, miR171a, miR396a, miR528) were highly expressed in *Brachypodium*, and were sequenced more than 1000 times each. The miR160a, miR164d, miR169f, miR172a, miR172b, miR319, miR390, miR393, miR394, miR395a, miR397a, miR529 and miR827 were moderately expressed, and were represented by the number of sequences varying between 10 and 100. The third category, whose expression level was low (< 10 sequences), was represented by miR162, miR169a, miR169c, miR169e and miR398 (Figure 2). Sequence analysis indicated that the relative abundance of certain members within a miRNA family varied greatly in *Brachypodium*. For instance, the sequencing frequency for miR166a was 29,730, whereas miR166g's sequencing frequency was only 1,145 (Figure 2). These results indicate that in one miRNA family different members show clearly different expression levels, probably because their expression is tissue- or developmental-specific.

### Genomic organization of the miRNAs

**Figure 3:**
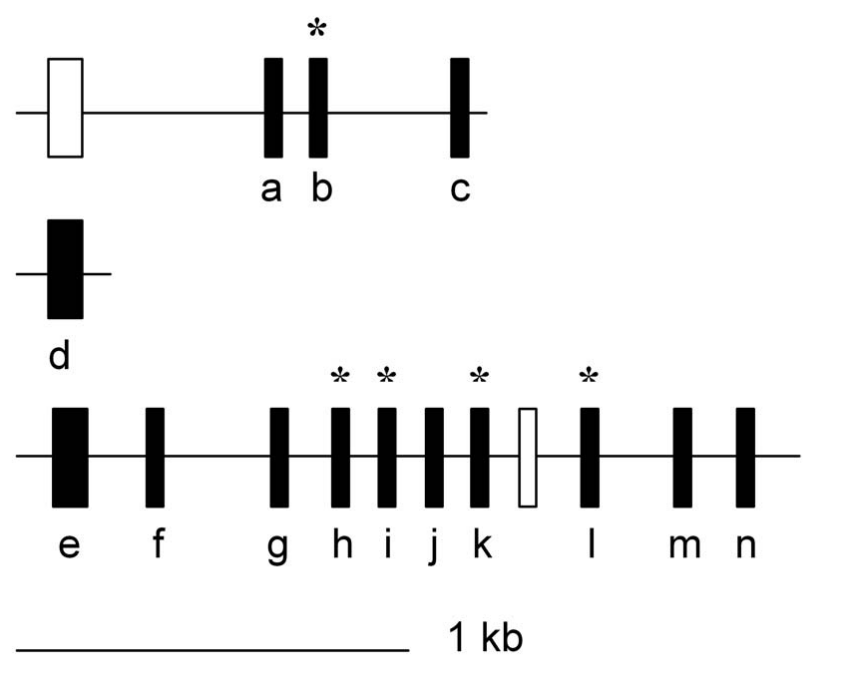
**Genomic organization of the miR395 family in *Brachypodium***. Thin black lines represent genomic DNA fragments. Small and big solid vertical bars represent type A and type B miR395 genes, respectively. Open vertical bars represent sequences showing similarity to miR395 genes, but containing no mature miR395 sequence. The location of miR395 genes are roughly in proportion to their real physical locations. Letters below the vertical bars indicate names of the genes. Asterisks denote miR395 genes whose miRNA* have been detected in our study. Scale bar represents 1 kb.

Among the 27 conserved *Bachypodium *miRNAs, 16 were encoded by a single copy in the *Brachypodium *genome, whereas the other 11 miRNAs had multiple loci (Table 2), which probably resulted from duplication events that were still active in the *Brachypodium *genome. For these 11 miRNAs, most of them only had 2-4 loci in the genome, only a few had more than 6 loci. For the predicted *Brachypodium *miRNAs, most of them had one single locus in the genome and only a few were represented by more than 2 loci (Table 3) (Additional file 1). According to the currently available gene annotation for the *Brachypodium *genome, most of these predicted miRNAs were mapped to the intergenic regions. Interestingly, for almost all the conserved miRNA families (except miR395) with multiple members, their family sizes in *Brachypodium *are much smaller than those in rice and *Populus*, and most of them have sizes similar to those in *Arabidopsis *(Table 4). For example, the number of family members for miR156, miR160, miR164, miR166, miR167, miR171 and miR396 in *Brachypodium *was similar to that in *Arabidopsis*, but much higher in rice and *Populus *(Table 4). For miR395, its size in *Brachypodium *was approximately half of that in rice (Figure 3, Table 4).

**Table 3:** Predicted *Brachypodium *miRNAs that are responsive to cold stress.

**Name**	**Sequence (5'-3')**	**L (nt)**	**Arm/nt**	**N**	**Location in the genome**	**AMFE** **(kcal mol^-1^)**	**CD** **NC/WC**
miR901T	UAUGCCAUGUCGUCACAUAUC	21	5'/104	1	super_0:10573853-10573873	-63.46	3/24
miR902T	UAGAUCUUUAAAUAAACGGAUG	22	5'/174	1	super_1:13020674-13020695	-49.71	4/22
miR903T	GGGGAAAAGAGAUUGAGGGAG	21	3'/92	1	super_0:36361618-36361638	-69.34	6/31
miR904T	UGUUCAUACGGUUGAUAGCAC	21	3'/144	1	super_11:5086279-5086299	-47.71	3/22
miR905T	UUCUUUGACCGAGCCUUUGAC	21	5'/140	1	super_4:26389353-26389373	-38.71	7/39
miR906T	UGGACUGCAGGUUUAUUUCGG	21	3'/116	1	super_10:314839-314859	-41.15	4/23
miR910T	UGUAGAUACUCUCUAAGGCUU	21	3'/87	1	super_1:8100215-8100235	-54.13	28/5
miR911T	AAGAAUUUAGGGACGGAGGGA	21	3'/86	2	super_277:408-428	-70.01	35/4
			3'/88		super_2:20949532-20949552	-45.00	
miR912T	ACUGGAUGGCACGGGAGCUAC	21	3'/103	1	super_4:17219372-17219392	-39.22	67/7
miR913T	UUUGAACUAAGAAGGGUCAAA	21	3'/128	3	super_1:16079568-16079588	-44.14	43/5
			3'/131		super_3:15856277-15856297	-50.91	
			3/132		super_7:14469193-14469213	-57.34	
miR914T	UUGAGCUAAGGAGGGUUGGAG	21	3'/100	1	super_11:2194743-2194763	-52.60	37/6
miR915T	UUGAACUAAGGAGGGUCAAUG	21	3'/103	1	super_0:16172946-16172966	-71.94	29/5
miR916T	AGGGCGAGGCAAAUGAUCAAA	21	3'/90	1	super_4:13613574-13613594	-66.22	33/6
miR917T	ACUUAUUUCGGGACGGAGGGC	21	3'/88	1	super_10: 6143242-6143262	-58.75	34/6
miR918T	AAAAUCGAGUAGCAGUCCGCG	21	3'/184	1	super_4:2249267-2249287	-40.59	41/5
miR919T	AAAAUCGAGUAGCAGCCCGUG	21	3'/185	1	super_1:24396584-24396604	-58.75	144/28
miR920T	AUCUUGGGCUCUAGGUAGGUU	21	5'/170	1	super_7:15735308-15735328	-58.58	25/5
miR921T	UUUCGGCUUCUAGGACCGGCU	21	5'/203	1	super_5:18782230-18782250	-54.33	15/3
miR922T	ACUUGUUUUGGGACGGAGGGA	21	3'/88	1	super_1: 22691473-22691493	-75.91	22/4
miR923T	UUAGGUGCUUUCGGCUUUGGC	21	5'/116	1	super_0:7108639-7108659	-57.16	32/6
miR924T	AGUAAUAUGUGUCGGAGGGGG	21	3'/100	1	super_5:7860249-7860269	-58.20	32/5
miR925T	UUACGUGAGUUAAAUCGUCGA	21	3'/100	1	super_0:9973530-9973550	-64.40	16/3
miR926T	AAGAAUUUAGGAAUGGAGGGA	21	3'/86	1	super_1:26695542-26695562	-45.58	36/6
miR927T	AGAAUUUAGGGAAGGAGGGAU	21	3'/88	1	super_0:27230875-27230895	-56.93	37/7
miR928T	ACUUAUUAUGGACCGGAGGGA	21	3'/71	1	super_3:10319482-10319502	-53.80	24/3

Name, the name of predicted cold-responsive *Brachypodium *miRNAs and underlines denote miRNAs whose miRNA* have been detected in Solexa sequencing analysis; Sequence, sequence cloned in small RNA libraries; L, the length of miRNA; Arm/nt, the miRNA location in the predicted hairpin structure (5' or 3' arm)/the length of the precursor; N, number of loci in the genome; Location, miRNA location in the genome; AMFE, the adjusted minimum free energy (MFE) representing the MFE of 100 nucleotides, it is calculated by (MFE ÷ length of miRNA precursor sequence)× 100; CD, the effect of cold-treatment on miRNA expression, NC/WC, normalized sequencing frequencies in the NC and WC library.

**Table 4:** Comparison of the number of miRNA family members in *Brachypodium*, rice, *Arabidopsis *and *Populus*.

**miRNA family**	***Brachypodium***	**Rice**	***Arabidopsis***	***Populus***
miR156	7	12	8	11
miR160	4	6	3	8
miR162	1	2	2	3
miR164	4	6	3	6
miR166	7	14	7	17
miR167	3	10	4	8
miR168	1	2	2	2
miR169	6	17	14	32
miR171	4	9	3	14
miR172	2	4	5	9
miR319	1	2	3	9
miR390	1	1	2	4
miR393	1	2	2	4
miR394	1	1	2	2
miR395	14	23	6	10
miR396	2	5	2	7
miR397	2	2	2	3
miR398	1	2	3	3
miR827	1	1	1	1

The number of miRNA family members in rice, *Arabidopsis *and *Populus *was obtained from the miRBase .

MiR395 belongs to conserved miRNAs that have been found across different plant species. Both miR395 and miR395* could be detected in the NC library, although their reads were not high. According to the detected miRNA and miRNA* sequences as well as results of BLASTn search based on sequences similarity, 14 precursors with reasonable minimum free energy value were identified for miR395 in the draft *Brachypodium *genome (Figure 3). They were named miR395a~miR395n according to the order of their genomic location. Additionally, two other sequences in the surrounding region showed high similarity to miR395 precursors, but they contained no mature miR395 sequence and were therefore not considered for further analysis in this study (Figure 3).

For all the miR395 precursors, the mature miRNAs are all located on the 3' arms. The precursors of miR395a~c are clustered together and so were the precursors of miR395e~m. (Table 2, Figure 3). It is interesting to note that miR395d in *Brachypodium *is not clustered with other family members (Figure 3) although the members of this family always form clusters in other plant species [49]. No miR395- precursor-like sequence was detected in its surrounding region. On the basis of size and sequence similarity, two types of miR395 precursors exist in *Brachypodium*. Type A includes miR395a~c and miR395f~n, and type B includes miR395d and miR395e. Both kinds of precursors show high sequence similarity to their homologs in rice (Additional file 4). The precursors of miR395a~c and miR395f~n show high sequence similarity, suggesting that they are derived from a series of gene duplication events. These precursors exhibit different levels of sequence similarity to one another, implying that the duplication events happened at different time points during the evolution history of the miR395 family. The precursors of miR395i and miR395k are nearly identical, implying a recent origin (Additional file 4).

### The impact of cold stress on conserved and species-specific miRNAs in *Brachypodium*

To detect the effect of cold stress on *Brachypodium *miRNAs, the expression of miRNAs in *Brachypodium *seedlings with (WC library) and without cold-treatment (NC library) were examined with Solexa technology. These two libraries were compared for their size distribution of small RNAs. In the NC library, the 21-24 nt RNAs represented the predominant species (about 80%), with 21-nt and 24-nt RNAs being the two most abundant classes (Figure 1a), consistent with the distribution patterns of small RNAs from other plant species [23,26,50]. However, in the WC library, about 25% of the total small RNAs were 19 nt in size, which was the most abundant class (Figure 1b). The high percentage of the 19-nt small RNAs in the WC library may result from the increased amount of RNA degradation products caused by the cold treatment.

Overall, approximately one-fourth of miRNAs showed altered expression (> 3 fold) in the cold-stressed *Brachypodium *seedlings, while the other miRNAs were expressed almost equally in the NC and WC libraries. In response to cold treatment, 3 known miRNAs and 25 predicted miRNAs showed significant changes (≥ 5 fold) in their expression levels. The most obvious change was observed for miR397, whose expression level increased about 15-fold in the WC library compared with that the NC library. The expression of miR169e and miR172b also showed more than 5-fold of increases in the WC library. The cold-induction of miR172a (about 3-fold) was not as significant as that of miR172b. Among the 25 predicted miRNAs with altered expression under cold stress, 6 were up-regulated and 19 were down-regulated (Table 3) (Additional file 5). Based on sequence similarity, these 25 predicted miRNAs were classified into 18 miRNA families. The sequences of miR913T, miR914T, miR915T showed high similarity and could be grouped as one family. It is the same case for miR911T, miR926T and miR927T as well as miR917T, miR922T and miR928T. The miR918T and miR919T could also be grouped as one family. For these miRNA families, all the members showed decreased expression in response to the cold treatment. Analysis of the isolation frequencies for these 25 cold-responsive predicted miRNAs indicated that most of them belonged to the moderately expressed miRNAs, with isolation frequencies in the range of 10 and 90 (Table 3, Additional file 1). The NC library was also searched for miRNA* sequences of these cold-responsive predicted miRNAs, and only the miRNA* for miR912T and miR918T were found. No miRNA* sequence was detected for other predicted miRNAs with cold response, probably because their isolation frequencies, about 10% of the frequencies of mature miRNAs [9], were too low.

To determine whether the 25 cold-related predicted miRNAs are conserved among other plant species, the nucleotide databases were searched with BLASTn [38] to identify their homologs and surrounding sequences. These cold-related predicted miRNAs were also aligned with the genome of the monocotyledonous model plant rice (*Oryza sativa*)  using PatScan [51]. Hairpin structures were predicted for these miRNA homologs with the RNAfold program  using the surrounding sequences. Some of these cold-related predicted miRNAs (miR911T-914T, miR918T, miR922T and miR928T) had homologs in the rice genome, some of them (miR911T and miR922T) also had homologs in the wheat and barley genome, but no homolog was found in the *Arabidopsis *or *Populus *genome, suggesting that these are monocot-specific miRNAs. For some of these cold-related predicted miRNAs, such as miR901T-906T and miR923T-927T, no homolog was found in other plant species, suggesting that they are *Brachypodium*-specific miRNAs (Additional file 6).

### Experimental validation of the cold-responsive expression of *Brachypodium *miRNAs

**Figure 4:**
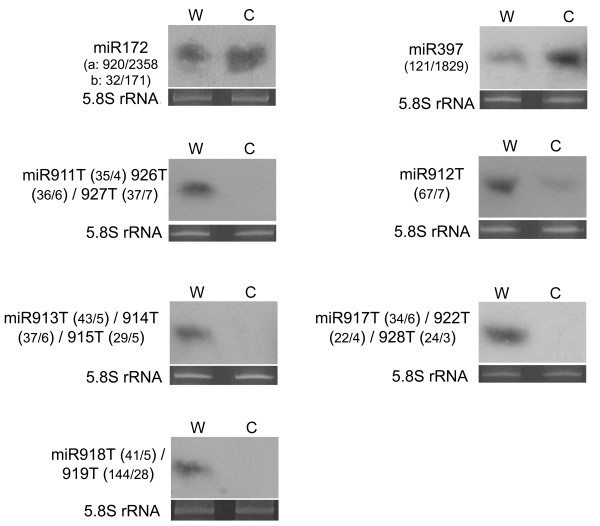
**Validation of cold-responsive expression of conserved miRNAs and predicted miRNAs in *Brachypodium***. Total RNA was isolated from the aerial parts of *Brachypodium *seedlings with (C) or without (W) cold-treatment and analyzed with RNA gel blots probed with endlabeled antisense oligonucleotides. The 5.8S rRNA bands were visualized by ethidium bromide staining of polyacrylamide gels and served as loading controls. Number in parentheses show the normalized sequencing frequencies of cold-responsive *Brachypodium *miRNAs in the NC/WC library. Note that the miR911T/926T/927T, miR913T/914T/915T, miR917T/922T/928T and miR918T/919T could not be distinguished because of high sequence similarity.

To confirm the expression of the identified miRNAs and their response to cold stress, the expression of all the known cold-responsive miRNAs and all the predicted miRNAs whose expression changed significantly (> 7 fold) after cold treatment were analyzed. Three known miRNAs and eight predicted miRNAs were selected for RNA gel blot analysis. The result showed that the expression of both miR172 and miR397 was up-regulated under the cold treatment (Figure 4). The expression of miR169e was difficult to be detected due to its sequence similarity to other members in the miR169 family and low expression level. Clear changes were observed for the expression of five analyzed predicted miRNAs (miR911T, miR912T, miR913T, miR917T, miR918T) after cold treatment (Figure 4). Two of the analyzed predicted miRNAs (miR901T and miR904T) did not show detectable hybridization signals, probably because their expression level was low (isolation frequency < 25). Their cold-responsive expression was then validated by Real-time PCR analysis (Additional file 7).

### Target predictions for cold-responsive *Brachypodium *miRNAs

It has been shown that plant miRNAs exhibit high degree of sequence complementarity to their targets, which allows for effective target prediction [52]. Target prediction analysis, therefore, was performed for the cold-related bdi-miRNAs. For *Brachypodium*, only draft genome sequence is now available, which has been annotated according to rice gene models as well as grass transcript assemblies and expressed sequence tags (ESTs). The accuracy and reliability of this notation needs further confirmation. The NCBI  and other database  contain *Brachypodium *ESTs, but the number is limited. Based on all the available data, the miRNA targets were predicted manually using the PatScan program [51] with penalty scores ≤ 3 for mismatched patterns in the miRNA/mRNA duplexes as described by Lu *et al*. [53] (Table 5) (Additional file 8).

**Table 5:** Predicted target genes of cold-responsive miRNAs identified in *Brachypodium *and their putative functions.

**miRNA**	**Putative function of target genes**	**Predicted target**
bdi-miR397	Laccase	Bradi1g24880.1 (2)
		Bradi2g55060.1 (2)
	Laccase precursor	Bradi4g44810.1 (3)
bdi-miR901T	LOB domain protein	Bradi1g13160.1 (2.5)

Number in parentheses show the scores of predicted miRNA targets based on rules described in [53].

For conserved miRNAs, their target sites should also be conserved across different plant species. As expected, the targets for conserved cold-related miRNAs in our system were similar or functionally related to previously validated plant miRNA targets. For example, bdi-miR397 could target laccase and laccase precursor (Table 5) (Additional file 8, Additional file 9). Laccase has also been predicted as the target of miR397 in *Arabidopsis *and rice [16,54]. Putative targets were also predicted for some newly identified cold-stress-related miRNAs (Table 5) (Additional file 8, Additional file 9). The target of this miR901T, a cold-induced predicted miRNA, is predicted to be a gene encoding LOB domain protein which is proved to be involved in various aspects of plant development [55-58].

## Discussion

The miRNAs are a group of small non-coding RNAs that play an important role in various developmental and stress response processes through negatively controlling of gene expression [59]. They have been identified in diverse plant species, and the large-scale miRNA identification in monocots with experimental approaches has mainly been performed in rice. Although miRNA identification has also been reported in wheat [25], extensive study was hampered by the unavailability of the whole genome sequence for wheat. *Brachypodium*, with close evolutionary relationship to wheat, has recently been proposed as a model temperate grass in monocots. The genome sequence of *Brachypodium distachyon *has been released ; . To our knowledge, few studies have been done on identifying and analyzing miRNAs in this plant species. In this paper, genome-wide analysis of *Brachypodium *miRNAs were performed with high-throughput sequencing technology and their response to cold stress was also analyzed, providing useful information to deepen our understanding of miRNAs in monocotyledonous plants.

### The miRNAs in *Brachypodium*

Solexa sequencing of the *Brachypodium *small RNA libraries revealed the existence of 27 conserved miRNAs as well as 129 predicted miRNAs (Table 2, Additional file 1), indicating that in *Brachypodium *only a small fraction of miRNAs are conserved miRNAs, the rest of them appear to be species-specific miRNAs. Most of the miRNA families conserved between *Arabidopsis*, *Populus *and rice were also identified in our dataset (Table 2) [1,60]. Sequence analysis indicated that conserved miRNAs in *Brachypoidium *showed higher similarity to their homologs in rice than in *Arabidopsis *and *Populus *(Table 2). MiR528, previously only identified in rice [61], was also found in *Brachypodium *(Table 2), indicating that it is not specific to rice. For predicted miRNAs, only these with cold stress response were further analyzed. Some of them had no homologs in other plant species and some appeared to be also conserved in wheat, rice and barley, but not in *Arabidopsis *and *Populus *(Additional file 6), indicating that they were either *Brachypodium*-specific or monocot-specific miRNAs. The predicted miRNAs exhibited relatively low expression levels (Additional file 1), in consistent with the notion that non-conserved miRNAs are often expressed at a lower level than conserved miRNAs. These miRNAs have not been reported in other plants before, possibly because their expression levels are low and need deeper sequencing to be discovered. High-throughput sequencing technology and whole-genome-scale data mining enable us to discover probably most of the miRNAs in *Brachypodium*, which is an important resource for miRNAs analysis. Deep sequencing of different species of *Brachypodium *or different kinds of samples may lead us to identify more novel bdi-miRNAs.

In monocotyledonous plants, the *Brachypodium *has close phylogenetic relationships with rice. Both of them belong to the Poaceae family, with rice in the Ehrhartoideae subfamily and *Brachypodium *in the Pooideae subfamily. The miRNAs in rice have been examined extensively. Therefore, deep sequencing analysis of *Brachypodium *provides a good chance to compare miRNAs at the whole genome level in these two closely related plant species. Interestingly, although the bdi-miRNAs and osa-miRNAs show high sequence similarity, their genome organization and family size are quite different.

Among the conserved *Brachypodium *miRNA families, miR395 is distinguished because its members are clustered in several plant genomes. This family, therefore, has been selected for genome organization analysis. According to the miRBase , the miR395 family has multiple clustered members in both eudicotyledonous and monocotyledonous plants. It also appears in *Physcomitrella patens*, the model moss species, but only has a single copy. In rice, this family is encoded by four clusters of 23 genome loci, including two 7-gene clusters, one 6-gene cluster and one 4-gene cluster. In *Brachypodium*, the miR395 family is encoded by 14 genome loci and two clusters are formed (Figure 3), suggesting that duplication evens in *Brachypodium *are not as active as that in rice. Although up to now, all the miR395 gene family members in other eudicotyledonous and monocotyledonous plant genomes are clustered, the *Brachypodium *miR395d was not clustered with other paralogs (Figure 3). Because miR395 only has one member in lower plant *Physcomitrella*, miR395d may be the ancient form of this family that is kept unchanged in *Brachypodium*, possibly under some kind of positive selection. It has been shown that the origin of the plant miRNAs is dependent on the occurrence of various duplications, probably followed by chromosomal rearrangements and loss of duplicated genes [24,49,62,63]. Thus, an alternative explanation is that the unclustered miR395d may result from the loss of miR395 genes happened after duplication events during the evolution of this family. If this is the case, the loss of miR395 genes probably occurred after the divergence of Ehrhartoideae (rice) and Pooideae (*Brachypodium*), because there is no unclustered miR395 gene in rice.

Comparative analysis showed that the sizes of conserved multiple-member miRNA families in *Brachypodium *were smaller than those in rice and *Populus*, most of them have similar sizes to those in *Arabidopsis *(Table 4). For example, seven families (miR156, miR160, miR164, miR166, miR167, miR171 and miR396) had similar number of members in *Brachypodium *and *Arabidopsis*, but their sizes were much larger in rice and *Populus *(Table 4). Considering the genome size of *Brachypodium *(about 300 Mb, The International *Brachypodium *Initiative, ), rice (389 Mb) [64], *Arabidopsis *(125 Mb) [65] and *Populus *(about 410 Mb) [66], it seems that the size of plant genomes has little effect on the number of miRNA family members. The genome size of *Brachypodium *is close to that of rice and *Populus*, compared with the size of the *Arabidopsis *genome. The miRNA families in *Brachypodium*, however, had much fewer members than those in rice and *Populus *(Table 4). One reasonable explanation is that the duplication events during the evolution of these miRNA families in *Brachypodium *are less active or the selection pressure on duplicated miRNAs is higher than that in rice and *Populus*.

The great difference in miRNA family size and genomic organization between *Brachypodium *and rice indicates that miRNA genes change their copy numbers and genome positions actively, probably to provide dosage effects or different regulatory patterns (the expression of miRNA genes is affected by surrounding sequences) in target gene regulation. In addition, these data show that the evolution of miRNA families in *Brachypodium *is not as active as that in rice, suggesting that *Brachypodium *could be used as a new platform for miRNA studies in monocots.

### The cold-responsive miRNAs in *Brachypodium*

Although miRNAs have been shown to play an important role in plant cold stress response, little information is available for monocotyledonous plants in this area [16-19]. *Brachypodium *is a potential model species for the cool season triticeae crops [28-31]. To study the cold-responsive miRNAs in this plant will provide useful information for improving the cold tolerance of economically important crops. In this study, the expression levels of miRNAs in *Brachypodium *with and without cold treatment were compared and the results indicated that the expression of about one-fourth of miRNAs was affected by cold stress. Among them, 3 conserved miRNAs (miR169e, miR172b and miR397) and 25 predicted miRNAs showed significant changes (Table 3, Figure 4, Additional file 7).

These 3 known cold-responsive miRNAs are conserved among dicotyledonous and monocotyledonous plants. MiR397 was shown to be cold responsive in *Arabidopsis *by sequencing of the stress-related small RNA library [16]. Then, miR169 and miR172 were found to be responsive to cold stress in *Arabidopsis *both through a computational, transcriptome-based approach and by microarray analysis almost simultaneously [17,18]. MiR169 and miR397 were also shown to be cold-upregulated in *Populus *[19]. The detection of the cold-induced expression for these miRNAs in *Brachypodium *indicates that they are also involved in cold stress response in monocotyledonous plants. Some previously identified cold-responsive conserved miRNAs, such as miR402 in *Arabidopsis *[16], were not found in our small RNA library, nor are they found in rice, suggesting that they probably are species-specific miRNAs and do not exist in *Brachypodium*. Some miRNAs, including miR166, miR319, miR393, and miR396, respond to cold stress in *Arabidopsis *[16,17], but in our experiment no obvious change was found for these miRNAs after the cold treatment. One explanation for these discrepancies is that the induction levels of these miRNAs are too low to be recognized as significant changes in our experimental system. It is also possible that these miRNAs show cold stress response only in specific tissues or at specific developmental stages in *Brachypodium*.

Previous studies put great emphasis on the cold-induced miRNAs, whereas the cold-suppressed miRNAs have received little research attention. Our study indicated that about one-third of cold-responsive miRNAs were up-regulated and two-thirds of them were down-regulated, indicating that both kinds of regulation for miRNAs were involved in cold response (Table 3, Figure 4). Interestingly, in our study all the cold-responsive miRNAs conserved in diverse plant species were up-regulated under cold stress condition. For the 19 cold-down-regulated predicted miRNAs, their expression was relatively low (Table 3, Additional file 1). These data suggest that *Brachypodium *responds to cold stress through both up- and down-regulation of miRNA expression, in which the up-regulation is conserved and probably more important in cold response.

## Conclusion

We have performed a genome-wide analysis of miRNAs in *Brachypodium*, providing a relatively complete view of bdi-miRNAs. Taking advantage of the good reproducibility of high-throughput sequencing, cold-responsive conserved as well as predicted *Brachypodium *miRNAs were identified, delivering new insights into the role of miRNAs in cold response of winter-habit monocotyledonous plants. Comparative studies of miRNAs in *Brachypodium *and rice suggest that the composition and location of miRNA families are different even in closely related plant species. Our study provides useful information for further analysis in this area. Characterization of miRNA targets and exploring miRNA regulation mechanism will deepen our understanding of plant miRNAs.

## Methods

### Plant material and growth conditions

Seeds of the *Brachypodium distachyon *(L.) Beauv. diploid line ABR5 were placed in petri dishes containing two layers of damp sterile filter paper. The seeds were first stratified at 4°C for one week to promote synchronous germination, then grown in a growth chamber at 24°C under a 16 hour/8 hour (light/dark) photoperiod with light intensity of approximately 5,000 lux.

### Small RNA library construction and Solexa sequencing

For small RNA library construction and deep sequencing, total RNA were isolated from 12-day-old seedlings using mirVana miRNA Isolation Kit (Ambion, Austin, TX, USA) following the manufacturer's protocol. The aerial part of seedlings treated or untreated with cold stress (4°C for 24 hour) were pooled and used for construction of the WC and NC small RNA libraries, respectively. For each library, approximately 20 μg of small RNA were subjected for sequencing using the Illumina-Solexa 1 G Genetic Analyzer in the Beijing Genomics Institute, according to the manufacturer's protocols. Briefly, the Solexa sequencing was performed as follows: RNA was purified by polyacrylamide gel electrophoresis to enrich for molecules in the range of 17-27 nt, then was ligated with 5' and 3' adapters. The resulted samples were used as templates for cDNA synthesis followed by PCR amplification. The obtained sequencing libraries were subjected to Solexa's sequencing-by-synthesis method. After the run, image analysis, sequencing quality evaluation and data production summarization were performed with Illumina/Solexa Pipeline.

### Bioinformatic analysis

High-quality small RNA reads larger than 18 nt were extracted from raw reads and mapped to the *Brachypodium *draft genome sequences obtained from the US Department of Energy Joint Genome Institute ;  using SOAP [35]. Matched sequences were then queried against non-coding RNAs from Rfam database  and NCBI Genbank database  by performing BLASTn search [38]. Any small RNAs having exact matches to these sequences were excluded from further analysis. MiRNAs were predicted with MIREAP . The secondary structures of the predicted miRNAs were confirmed by RNAfold .

The miRNA target candidates were predicted using the PatScan program [51] based on methods described in [53], with penalty scores ≤ 3 for mismatched patterns in the miRNA/mRNA duplexes. Putative target genes were manually selected from these candidates based on their localization in the *Brachypodium *genome . Functions of the predicted target genes were assigned manually according to the functions of the best hits from the BLAST search [38,67] against the NCBI database .

For predicted novel miRNA sequences, their conservation in other plant species was examined by searching the nucleotide databases with BLASTn [38] to identify their homologs and surrounding sequences. These cold-related miRNAs were also aligned with the rice genomes  using PatScan [51]. To analyze whether the matched sequence could form a suitable hairpin, sequences surrounding the matched sequence (100~200 nt to either side) were extracted and run through RNAfold .

### RNA gel blot analysis

Approximately 20 μg of total RNA (prepared as described above) was separated on 15% polyacrylamide denaturing gels. RNAs were then electrophoretically transferred to Hybond-N+ membrane (GE Healthcare UK Ltd., Little Chalfont, Buckinghamshire, UK). Membranes were chemically crosslinked mediated by 1-ethyl-3-(3-dimethylaminopropyl) carbodiimide (EDC) (Sigma-Aldrich, St. Louis, MO, USA) as described by [68] and hybridized with DNA oligonucleotides complementary to predicted miRNA sequences, which had been end-labelled with [γ-^32^P]ATP by T4 polynucleotide kinase (New England Biolabs, Ipswich, MA, USA). Non-incorporated nucleotides were removed using microspin G-25 column (GE Healthcare UK Ltd., Little Chalfont, Buckinghamshire, UK). Membranes were prehybridized for at least 3 hour and hybridized overnight at 37°C in ULTRAhyb-Oligo hybridization buffer (Ambion, Austin, TX, USA). The membranes were briefly air dried and then exposed to x-ray films for autography at -80°C. Images were acquired by scanning the films.

## Abbreviations

CT: cold-treatment; DCL1: Dicer-like 1; EDC: 1-ethyl-3-(3-dimethylaminopropyl) carbodiimide; ESTs: expressed sequence tags; miRNAs: microRNAs; NCBI: National Center for Biotechnology Information; nt: nucleotides; RISC: RNA-induced silencing complex; UTR: untranslated region; WT: wild type.

## Authors' contributions

KC JZ YX designed the experiments; JZ QH YX performed the experiments; JZ KC YX analyzed the data: JZ contributed reagents/materials/analysis tools; JZ KC wrote the paper. All authors read and approved the final manuscript.

## Supplementary Material

Additional file 1 **The predicted *Brachypodium miRNAs***. is a table listing all the predicted *Brachypodium *miRNAs. Click here for file

Additional file 2 **The secondary structure of predicted *Brachypodium *miRNAs**. is a figure showing the secondary structure of all predicted *Brachypodium *miRNAs. Click here for file

Additional file 3 **The secondary structures of conserved *Brachypodium *miRNAs**. is a figure showing the secondary structure of conserved *Brachypodium *miRNAs. Click here for file

Additional file 4 **Multiple sequence alignment of miR395 genes in *Brachypodium *and their homologs in rice**. is a figure showing multiple sequence alignment of *Brachypodium *miR395 genes as well as their homologs in rice, performed with the ClustalW 1.83 program. Click here for file

Additional file 5 **The secondary structures of cold-responsive predicted *Brachypodium *miRNAs**. is a figure showing the secondary structure of cold-responsive predicted *Brachypodium *miRNAs. Click here for file

Additional file 6 **The conservation of cold-responsive predicted *Brachypodium *miRNAs**. is a table listing the identified homologs of cold-responsive predicted *Brachypodium *miRNAs in other plant species. Click here for file

Additional file 7 **Real-time PCR validation of the cold-responsive expression of predicted miRNAs (miR901T and miR904T) in *Brachypodium***. is a figure showing the Real-time PCR analysis results of the levels of predicted *Brachypodium *miRNAs (miR901T and miR904T) in seedlings with and without cold-treatment. Click here for file

Additional file 8 **Alignment of cold-responsive miRNAs (bdi-miR901T and bdi-miR397) identified in *Brachypodium *to their predicted target mRNAs**. is a figure showing the alignment of cold-responsive miRNAs identified in *Brachypodium *to their predicted target mRNAs. Click here for file

Additional file 9 **Real-time PCR analysis for the expression of predicted miRNA target genes in *Brachypodium *after cold-treatment**. is a figure showing the results of the Real-time PCR analysis for the expression of predicted miRNA target genes in *Brachypodium *seedlings with and without cold-treatment. Click here for file
