# Supplementary material for: Deep sequencing of Brachypodium small RNAs at the global genome level identifies microRNAs involved in cold stress response
Source: BMC Genomics. 2009 Sep 23;10:449. doi: 10.1186/1471-2164-10-449 (PMC2759970; doi:10.1186/1471-2164-10-449)
Supplement: Additional file 2 — The secondary structure of predicted Brachypodium miRNAs. is a figure showing the secondary structure of all predicted Brachypodium miRNAs. [file 1471-2164-10-449-S2.pdf]

M1（ΔG = -82.20）

```
          10        20        30        40        50        60
    -         C                                      C  AA     U
AUAC UACUCCCUC GAUCCAUAAUAGGUGUCUUGGAUUUAGUACAAAGUUAU AU  UAAG \
UGUG AUGAGGGAG CUAGGUAUUAUUCACAGAACCUAAAUCAUGUUUCAAUG UG  GUUC G
    A         U                                      U  AG     U
    120       110       100        90        80        70
```

sRNA (5' to 3' orientation) mapped to this predicted precursor hairpin:

in the NC library:

CUUAUUAUGGAUCUGAGGGAG (sequencing frequency: 10)

in the WC library:

CUUAUUAUGGAUCUGAGGGAG (sequencing frequency: 6)

M2 （ΔG = -68.30）

```
           10         20        30        40
         A   C               -           G
 CCUCUAAUC CUU AAGGCAUUUUAUGAA GAAAAAACCCUG A
 GGAGAUUAG GAA UUUCGUAAAAUACUU CUUUUUUGGGAC U
         G   C               U           C
       80        70        60        50
```

sRNA (5' to 3' orientation) mapped to this predicted precursor hairpin:

in the NC library:

UAAUCACUUCAAGGCAUUUUA (sequencing frequency: 8)

in the WC library:

UAAUCACUUCAAGGCAUUUUA (sequencing frequency: 7)

M3 （ΔG = -49.80）

```
          10        20        30        40        50        60
                            AUUCAAAU   ACCAAAUA       A-    U
 UAAUAUUUUCUCUGUCCCAUAUUAAGUG        UUG        UGGAUGU  UGUA G
 AUUAUGAAAGAGGCAGGGUAUAAUUCAU        AAU        AUCUGCA  AUAU C
                            GUAAAGAU   GUACAUAG       AA    U
     120       110       100        90        80       70
```

sRNA (5' to 3' orientation) mapped to this predicted precursor hairpin:

in the NC library:

UUAAUAUGGGACGGAGAAAGU (sequencing frequency: 5)

in the WC library:

UUAAUAUGGGACGGAGAAAGU (sequencing frequency: 9)

M4 （ΔG = -66.00）

```
       10        20        30        40        50
      AUU-                                    AUAUUU
UCUCUC    UAUUCUAUGCCAUGUCGUCACAUAUCCUACAUGGCA      \
AGAGAG    GUAAGAUACGGUGUAGUAGUGUAUAGGAUGUAUCGU      A
      GAGC                                    ACAACC
    100        90        80        70        60
```

sRNA (5' to 3' orientation) mapped to this predicted precursor hairpin:

in the NC library:

UAUGCCAUGUCGUCACAUAUC (sequencing frequency: 3)

in the WC library:

UAUGCCAUGUCGUCACAUAUC (sequencing frequency: 24)

M5 （ΔG = -49.70）

```
       10        20        30        40
                           GAC            A
AUAUACUCCCUCUGUCUCAAAAUAAGU   GUGGAUUUGUAUAG U
UAUAUGAGGGAGGCGGGGUUUUAUUCA   CACCUAAACAUAUU U
                           AUA            U
 90        80        70        60        50
```

sRNA (5' to 3' orientation) mapped to this predicted precursor hairpin:

in the NC library:

ACUUAUUUUGGGGCGGAGGGA (sequencing frequency: 8)

in the WC library:

ACUUAUUUUGGGGCGGAGGGA (sequencing frequency: 7)

M6 （ΔG = -106.90）

```
        10        20        30        40        50          60          70        80          90
      CG  C                    A         A   UC          --              CA        UUA        GA
UCCCUC  UU CUAAUUAAUUGGUGCCGGCU UUUUGCAAA AAA  CUUGUAAAUU  CGAAAUAAACCU    UUUUCAA    AACGUG  \
AGGGAG  AA GAUUAAUUAACCACGGCCGA AAAACGUUU UUU  GGACAUUUAG  GUUUUAUUUGGA    AAAAGUU    UUGCGC  C
      AU  A                    C         C   GA          AG              --         UUA       GU
         170       160       150       140       130       120        110       100
```

sRNA (5' to 3' orientation) mapped to this predicted precursor hairpin:

in the NC library:

CCUAAUUAAUUGGUGCCGGCU (sequencing frequency: 8)

in the WC library:

CCUAAUUAAUUGGUGCCGGCU (sequencing frequency: 7)

M7 （ΔG = -90.60）

```
        10        20        30        40        50
   U               G                  G                 CAU
UUC GCUUCAGCUUUUGCU CUUCUAGCCCUCUAAAAA CACUUCUCCCGUUUACA   \
AAG CGAAGUCGAAAACGA GAAGAUCGGGAGAUUUUU GUGAAGAGGGCAAAUGU   G
   U               A                  G                 ACA
       110       100        90        80        70
```

sRNA (5' to 3' orientation) mapped to this predicted precursor hairpin:

in the NC library:

AGAAGUGGUUUUUAGAGGGCU (sequencing frequency: 8)

in the WC library:

AGAAGUGGUUUUUAGAGGGCU (sequencing frequency: 19)

M8 （ΔG = -58.00）

```
        10        20        30         40        50        60        70        80
       C  UC         A        A             C  C                AAAU   A   GAAA       U
AGUCAAA UU  UCAACUUUG CCGAGUUU UAGAAAAAAGUAC AA AUAUGUAACACCAAAU    GCA UAU    ACUAUU G
UCAGUUU AA  AGUUGAAAU GGUUUAAA AUCUUUUUUUGUG UU UAUAUGUUGUGGUUUG    CGU GUA    UGAUGA A
       C  CC         -        C             U  A                AU--   A   AUCC       U
 170       160        150       140       130       120       110         100         90
```

sRNA (5' to 3' orientation) mapped to this predicted precursor hairpin:

in the NC library:

UUUGACCGAGUUUAUAGAAAA (sequencing frequency: 4)

in the WC library:

UUUGACCGAGUUUAUAGAAAA (sequencing frequency: 8)

M9 （ΔG = -76.72）

```
        10         20        30        40        50
       AUU-                                    AUAUU
UUCUCUC    UAUUCUAUGCCAUGUCGUCACAUAUCCUACAUGGCA      U
GAGAGAG    GUAAGAUACGGUGUAGUAGUGUAUAGGAUGUAUCGU      A
       GAGC                                    ACAACC
    100        90        80        70        60
```

sRNA (5' to 3' orientation) mapped to this predicted precursor hairpin:

in the NC library:

UGUGAUGAUGUGGCAUAGAAU (sequencing frequency: 31)

in the WC library:

UGUGAUGAUGUGGCAUAGAAU (sequencing frequency: 45)

M10 （ΔG = -45.30）

```
        10        20        30        40        50        60
      GA  U    ACAUA  C     A   G        G   G  A         AA
GAUGGU  UU AGCA     CU CCUCC UCC GAAAUAAG GAC UG AUUUGUAUA  A
CUAUCG  AG UUGU     GA GGAGG AGG UUUUGUUC CUG AU UAAACAUAU  A
      G-  U    AACAG  A     C   G        A   A  C         CU
 120        110       100        90        80        70
```

sRNA (5' to 3' orientation) mapped to this predicted precursor hairpin:

in the NC library:

UCACUUGUUUUGGGACGGAGG (sequencing frequency: 6)

in the WC library:

UCACUUGUUUUGGGACGGAGG (sequencing frequency: 6)

M11 （ΔG = -48.69）

```
        10        20        30        40        50        60
UAC           G    U        UCUCAAAU    CC         UG   AA
   UACUCCCUCCG CUGG AUUACUUG        UUGC  AUGUAUCUA  UGU  \
   AUGAGGGAGGC GGCC UAAUGAAC        AAUG  UACAUAGAU  GCA  A
AUC           A    U        UACUUUAU    --         CU   AA
 120       110       100        90        80        70
```

sRNA (5' to 3' orientation) mapped to this predicted precursor hairpin:

in the NC library:

AGUAAUUCCGGACGGAGGGAG (sequencing frequency: 7)

in the WC library:

AGUAAUUCCGGACGGAGGGAG (sequencing frequency: 4)

M12 （ΔG = -54.19）

```
        10         20         30         40         50         60          70
        U          C    C   AAAAUAG    UA  AUAUAUAUAU    A      UG   UC      UU  U  UCG
UAUUGUUUU CUUUGACCGAGC UUUGA CAA      UCUAU  AU         UGUGA AUAAAUU  AUA  AUUGUG  CG GU   A
AUAACAAAA GAAACUGGCUUG AAACU GUU      AGAUA  UG         ACACU UGUUUAG  UAU  UGAUAU  GU CA   A
```

```
     -           C     A    ACUAAUA     GC  CACACUGAAU     A          --    --        UU   U   UGA
140         130          120              110          100          90                80
```

sRNA (5' to 3' orientation) mapped to this predicted precursor hairpin:
in the NC library:
UUCUUUGACCGAGCCUUUGAC (sequencing frequency: 7)
in the WC library:
UUCUUUGACCGAGCCUUUGAC (sequencing frequency: 39)

M13 （ΔG = -58.20）

```
                          10         20         30         40         50
                    --               C            C  GU             A
             AAAUCU  UACUCCCUCCGAC CAUAUUACUUG CG  GAUUUAGUGCA C
             UUUAGA  AUGGGGGAGGCUG GUAUAAUGAAC GC  CUAAAUCAUGU U
                    UC               U            A  GU             U
             100         90          80        70         60
```

sRNA (5' to 3' orientation) mapped to this predicted precursor hairpin:
in the NC library:
AGUAAUAUGUGUCGGAGGGGG (sequencing frequency: 32)
in the WC library:
AGUAAUAUGUGUCGGAGGGGG (sequencing frequency: 5)

M14 （ΔG = -47.56）

```
         10         20         30         40           50         60
      C                    CUCAAAU  CC       GA-       GUAUU   AA
AUACUC  CUCCGUCCCAUAAUUCUUGU       UG  AAAUAU   AUGUAU      CA   A
UAUGAG  GAGGCAGGGUAUUAAGAACA       AC  UUUAUA   UGCAUA      GU   A
      A                    AGACACA  --       AUG       AAUCU   AA
 120         110        100         90         80          70
```

sRNA (5' to 3' orientation) mapped to this predicted precursor hairpin:
in the NC library:
AACAAGAAUUAUGGGACGGAG (sequencing frequency: 5)
in the WC library:
AACAAGAAUUAUGGGACGGAG (sequencing frequency: 4)

M15 （ΔG = -125.30）

```
      10        20        30        40        50        60        70         80          90       100
       U          A            CC                A  U         A         -   AUA       CU   C      U
```

```
CCUAAUUAAU GGCGCCGGCU UUUUGCAAAGAAA  CUUGUAAAUUUCCGAA UA ACCUGCA UUCACAUCU UCA   AACGUGGA  GCA GUUGUUU G
GGAUUAAUUA CCGCGGCCGA AAAACGUUUUUUU  GGACAUUUAAAGGCUU AU UGGACGU AGGUGUAGA AGU   UUGCGCCU  UGU CAACAAA A
         U         C          UU               A  U      C        U  C--        UU   A     A
    200      190      180      170      160      150      140       130      120          110
```

sRNA (5' to 3' orientation) mapped to this predicted precursor hairpin:

in the NC library:

UUUUUUGCAAAACAGCCGGCG (sequencing frequency: 4)

in the WC library:

UUUUUUGCAAAACAGCCGGCG (sequencing frequency: 5)

M16 （ΔG = -57.80）

```
        10        20           30        40
      C   C                  --          AG
GUACUC CUC GUCCCAAAAUAAGUGACA  AUUUGUAUA  A
UAUGAG GAG CAGGGUUUUAUUCACUGU  UAAACAUGU  A
      U   A                  AC          CU
    80        70        60        50
```

sRNA (5' to 3' orientation) mapped to this predicted precursor hairpin:

in the NC library:

CUUAUUUUGGGACAGAGUGAG (sequencing frequency: 3)

in the WC library:

CUUAUUUUGGGACAGAGUGAG (sequencing frequency: 6)

M17 （ΔG = -101.70）

```
        10        20        30        40        50        60        70        80
      C      CCA  A          A        C   G                  A   CAA  AA  --  GAC
CUAAAUU UGAGA   GGC CAUUUGACGGC AAUCUGAC GGUU GGCCUACCGGUCAGUUGCCAC UGG   AU  AC  CAG  C
GGUUUGA ACUCU   CCG GUAAACUGUCG UUAGACUG CCAG CCGGGUGGUCAGUCAACGGUG ACC   UG  UG  GUC  A
      C      AGA  -          C        A   G                  G   AAG  GA  AA  AGG
       170       160        150       140       130       120        110        100        90
```

sRNA (5' to 3' orientation) mapped to this predicted precursor hairpin:

in the NC library:

UGAGACCAGGCACAUUUGAC (sequencing frequency: 6)

in the WC library:

UGAGACCAGGCACAUUUGAC (sequencing frequency: 5)

M18 （ΔG = -57.50）

```
       10        20        30        40        50        60       70
```

```
        UUA       UCAAAAAUA       A        UC            UG            CAAGU
GCAAUU    GUUAGC            CUACUC  CUCCGUU   UAAAUUCUUGU   UGGUUUUAGUU      \
UGUUGA    CGAUUG            GAUGAG  GAGGCAA   AUUUAAGAACA   AUCAAAGUCAA      U
        UAC       UG--ACU-        G        GA            GU            UAAAC
        130        120         110        100         90         80
```

sRNA (5' to 3' orientation) mapped to this predicted precursor hairpin:

in the NC library:

AAGAAUUUAAGAACGGAGGGA (sequencing frequency: 6)

in the WC library:

AAGAAUUUAAGAACGGAGGGA (sequencing frequency: 9)

M19 （ΔG =-58.80）

```
        10          20          30          40
       A     -           U              A
 UCCCUCC  UCCA  AAAUAAGUGAU  UGAAUUUGUAUAA  U
 AGGGAGG  AGGU  UUUAUUCACUG  ACUUAAACGUGUU  U
       C     C           U              C
 80         70          60          50
```

sRNA (5' to 3' orientation) mapped to this predicted precursor hairpin:

in the NC library:

CACUUAUUUCUGGACGGAGGG (sequencing frequency: 7)

in the WC library:

CACUUAUUUCUGGACGGAGGG (sequencing frequency: 13)

M20 （ΔG = -76.30）

```
        10         20         30          40         50         60
            A   C               ACUCC      CA      A  UCC
CUGUUUGUUUGG UUU UGCUUCAGCUUUU       GAAGCUG  GGAAGC CC    \
GACAAACAAACC GAA ACGAAGUCGAAAA       CUUCGAC  UUUUUG GG    A
            G   C               AAUGC      --      -  CAA
.       130        120                     80          70
                                               90
                                        AC     U     U
                                          UGUG  GCUUU  U
                                          ACAU  CGGAA  A
                                        CC     U     A
                                   110        100
```

sRNA (5' to 3' orientation) mapped to this predicted precursor hairpin:

in the NC library:

UGGAUUUCUGCUUCAGCUUUU (sequencing frequency: 4)
in the WC library:
UGGAUUUCUGCUUCAGCUUUU (sequencing frequency: 3)

M21 （ΔG =-71.10）

```
          10        20        30        40        50        60
       U--   G  UG UU                           C  AC         G
GAAUCUG   GCU GA  A  UACUCCCUCCGUUCCAUAAAGAUUGGC UG  UUUGAACUA C
CUUAGGU   CGG UU  U  AUGAGGGAGGCAAGGUGUUUCUAACCG AC  AAACUUGAU U
       UGC   A  GU U-                           U  CA         A
   120       110        100        90        80        70
```

sRNA (5' to 3' orientation) mapped to this predicted precursor hairpin:
in the NC library:
AAUCUUUGUGGAACGGAGGGA (sequencing frequency: 34)
in the WC library:
AAUCUUUGUGGAACGGAGGGA (sequencing frequency: 17)

M22 （ΔG =-84.76）

```
        10        20        30        40        50         60        70
              A  A     G   C      C                        -  AGA    GCACUUU
GAGUAAAAUACACC CU GUCCA AAA UCGGAG AAAUGAACACUUUAGUCCACGAAC UC   AAAC       \
CUUAUUUUAUGUGG GA CAGGU UUU AGUCUC UUUACUUGUGAAAUCAGGUGCUUG GG   UUUG       A
              G  C     A   A      C                        U  GA-    ACCCUUC
 .       140       130       120       110       100        90         80
```

sRNA (5' to 3' orientation) mapped to this predicted precursor hairpin:
in the NC library:
UGGACCAGGGGUGUAUUUUAU (sequencing frequency: 7)
in the WC library:
UGGACCAGGGGUGUAUUUUAU (sequencing frequency: 9)

M23 （ΔG = -118.90）

```
        10        20        30        40                50        60        70        80          90
     A             C     U  U           C       -UAGUCU-      U  C        U            C     UA    UGC
AGUUU GUCAAACCUGGUU UGACC GC GGAUGGUGGUU UAUUGCC        ACGUCA CU AUGGUGAG CGAUUUCGUGGA CCCAC   AGA   A
UCAAA CAGUUUGGACUAA ACUGG UG UCUACCACCAA GUAACGG        UGCAGU GA UGCCGCUC GCUAAGGUACCU GGGUG   UCU   U
     C             A     U  C           A       UACACAGG      C  C        U            A     --    CCC
  220       210       200       190       180       170       160       150        140              100
                                                                                         110        120
```

```
                                                                 AUUU    C
                                                                     GCUC C
                                                                     CGAG A
                                                                 UAAU    C
                                                                   130
```

sRNA (5' to 3' orientation) mapped to this predicted precursor hairpin:

in the NC library:

UGACCUGCUGGAUGGUGGUUCU (sequencing frequency: 4)

in the WC library:

UGACCUGCUGGAUGGUGGUUCU (sequencing frequency: 7)

M24 （ΔG =-61.50）

```
        10         20         30         40         50         60
   A  U   G      C           C           CAU  GC      CAAG   GA   A
GGA GC AGU GAGGA UCCAAAGGGAU GCAUUGAUCU   CA UGCAUC    CAU  AUC A
UCU CG UCA CUCUU AGGUUUCCCUA CGUGACUAGA   GU ACGUAG    GUA  UAG C
   C  C   G      A           A           AC-  AU      CUAG   GG   G
        120        110        100          90         80         70
```

sRNA (5' to 3' orientation) mapped to this predicted precursor hairpin:

in the NC library:

UCAGUGCAAUCCCUUUGGAAU (sequencing frequency: 55)

CAGUGCAAUCCCUUUGGAA (sequencing frequency: 1)

in the WC library:

UCAGUGCAAUCCCUUUGGAAU (sequencing frequency: 170)

CUCCAAAGGGAUCGCAUUGAU (sequencing frequency: 9)

CAGUGCAAUCCCUUUGGAA (sequencing frequency: 1)

M25 （ΔG = -97.60）

```
        10         20          30         40         50          60
               -   A           CUC   C          AC      C  CA
  GCGGGAGGAGUCGU CUC UCGCCGGUGC   CGC GCCGCCCCG  CUGCUA GU   \
  CGCCUUCUUCAGCA GAG AGCGGCCACG   GCG CGGUGGGGC  GACGAU CA   C
               C   C           CGC   U          --      U  AC
    160          150        140        130          120        110
```

```
                                                                  90
                                                                 A   G   G
                                                                   GC CCG C
                                                                   CG GGU C
                                                                 A   G   A
```

```
                                                            100
```

sRNA (5' to 3' orientation) mapped to this predicted precursor hairpin:

in the NC library:

ACCGGCGACGAGCACGACUUC (sequencing frequency: 4)

in the WC library:

ACCGGCGACGAGCACGACUUC (sequencing frequency: 4)

M26 （ΔG = -71.70）

```
      10         20         30         40           50         60
      C   -                                  UA         A     G
AUCCAC CAA CUGAAAUAUUUAAAAAUAAUUGGAGCAAC   AGCUAUCA GAUUG A
UAGGUG GUU GACUUUAUAGAUUUUUAUUGACUUUGUUG   UCGAUAGU UUGAC U
      U   G                                  --         A     C
      170        160        150        140                80         70
                                                          90         100
                                                     GCUAUACGGAGGGGAG   AAA
                                                                     GGA    G
                                                                     CCU    G
                                                     UGAAAGGAACACAACA    GAG
                                                   130        120        110
```

sRNA (5' to 3' orientation) mapped to this predicted precursor hairpin:

in the NC library:

UAGAUAUUUCAGGUUGUGUGGA (sequencing frequency: 210)

CACCCAA CUGAAAUAUUUAA (sequencing frequency: 26)

GAUAUUUCAGGUUGUGUGGA (sequencing frequency: 1)

UUAGAUAUUUCAGGUUGUGUGG (sequencing frequency: 1)

UUAGAUAUUUCAGGUUGUGUG (sequencing frequency: 1)

in the WC library:

UAGAUAUUUCAGGUUGUGUGGA (sequencing frequency: 358)

CACCCAA CUGAAAUAUUUAA (sequencing frequency: 25)

UAUUUCAGGUUGUGUGGAU (sequencing frequency: 2)

GAUAUUUCAGGUUGUGUGGA (sequencing frequency: 1)

M27 （ΔG = -55.40）

```
        10         20         30         40         50
        CU                     G  A    A  U  A
AUAUACUA  CCUCUGUUCCUAAAUUCUUGUC UU UUUU GU CA A
UAUAUGAU  GGAGACAAGGAUUUAAGAACAG GA AAAA CA GU U
        -U                     G  C    G  U  U
    90         80           70          60
```

sRNA (5' to 3' orientation) mapped to this predicted precursor hairpin:

in the NC library:

AGAAUUUAGGAACAGAGGUAG (sequencing frequency: 7)

in the WC library:

AGAAUUUAGGAACAGAGGUAG (sequencing frequency: 3)

M28 （ΔG = -82.00）

```
          20        30        40        50        60        70
             A  -   C  -                      C        A        A   G
    GCAAGCAGG UC CGC UA AUCCCGCUUCUAAGUCAAUCUC AAAUCCAC AGUCAAAU UAA \
    CGUUCGUCC AG GUG AU UAGGGCGAAGAUUCAGUUAGAG UUUAGGUG UCAGUUUA GUU C
             C  A   A  A                      A        C        A   A
    130       120       110       100       90        80
```

sRNA (5' to 3' orientation) mapped to this predicted precursor hairpin:

in the NC library:

AGAUUGACUUAGAAGCGGGAU (sequencing frequency: 5)

in the WC library:

AGAUUGACUUAGAAGCGGGAU (sequencing frequency: 12)

M29 （ΔG = -50.80）

```
         10        20         30        40         50         60
    -    U   G           CC-     CCACGU  CC   U  U  U  CA   A    U
GGCG CCGU UAU GUCCGCCAAGC   GUUUA      UC  CAG GC GU CA  AUC GCUG C
CUGC GGCA AUG CAGGCGGUUCG   CAAAU      AG  GUC CG CA GU  UAG CGGC A
    G    U   A           CAA     CAUCCU  AA   G  U  C  UU   U    C
  120       110       100              90        80        70
```

sRNA (5' to 3' orientation) mapped to this predicted precursor hairpin:

in the NC library:

UUGGCGGACAGUAUACGGGCGU (sequencing frequency: 8)

in the WC library:

UUGGCGGACAGUAUACGGGCGU (sequencing frequency: 5)

M30 （ΔG = -60.92）

```
          10         20         30         40
        U            A  A   C                   G
CAUGUAC CCCUCCGUCUC AA UAA UGACGUGGAUUUGUAUAA U
GUGUAUG GGGAGGCAGGG UU AUU ACUGCACUUAAACAUAUU U
```

```
          U                 C   A    C                         C
      90          80          70          60          50
```

sRNA (5' to 3' orientation) mapped to this predicted precursor hairpin:

in the NC library:

CACUUAAUUCGGGACGGAGGG (sequencing frequency: 5)

in the WC library:

CACUUAAUUCGGGACGGAGGG (sequencing frequency: 17)

M31 （ΔG = -120.74）

```
          10        20        30        40        50        60        70        80        90        100       110
      CA-                CU    C         GAA    C      C            G          CACCUAA      A     CUG     UUA     G
UAUGGGC   GUUCUUUUCGGCUUU  GGAC GGCUUUUCC   AAGC GCUCUC CCUAGCUUUUUG AGAAGCCGC       AUUUG UUAGG   CCAAA   GUUU C
AUAUCCG   CAAGAAAAGCCGAAG  UCUG CCGAAGAGG   UUCG CGGGGG GGAUCGAAGGAC UCUUCGGCG       UAAAU GAUCC   GGUUU   CAAA C
      ACG                AU    A         AUC    A      U            A          UAAAGUC      C     AAA     CAA     U
   220       210       200       190       180       170       160       150       140       130       120
```

sRNA (5' to 3' orientation) mapped to this predicted precursor hairpin:

in the NC library:

UUUCGGCUUUCUGGACCGGCU (sequencing frequency: 7)

in the WC library:

UUUCGGCUUUCUGGACCGGCU (sequencing frequency: 19)

M32 （ΔG = -64.40）

```
           10        20        30        40        50
                                        AAAAA    UA
GUCGACGAUUUAACUCACGUAACAUAAGUUUGAACUCAC      GUA  U
UAGCUGCUAAAUUGAGUGCAUUGUAUUCAAACUUGAGUG      UAU  C
                                        CAUUG    UA
100      90        80        70        60
```

sRNA (5' to 3' orientation) mapped to this predicted precursor hairpin:

in the NC library:

UUACGUGAGUUAAAUCGUCGA (sequencing frequency: 16)

in the WC library:

UUACGUGAGUUAAAUCGUCGA (sequencing frequency: 3)

M33 （ΔG = -74. 10）

```
         10        20         30        40        50
   -                     C            UU     CA     UG
```

```
 CC  CAUUGACUCUCCUUAGUUCAA  CAAAUGAACUGG   UUUU    AAAGU   U
 GG  GUAACUGGGAGGAAUCAAGUU  GUUUACUUGAUU   AAAA    UUUUA   G
   A                       A              UU      --       CU
     100        90          80         70         60
```

sRNA (5' to 3' orientation) mapped to this predicted precursor hairpin:

in the NC library:

sRNA (5' to 3' orientation) mapped to this predicted precursor hairpin:

in the NC library:

UUGAACUAAGGAGGGUCAAUG (sequencing frequency: 29)

in the WC library:

UUGAACUAAGGAGGGUCAAUG (sequencing frequency: 5)

M34 （ΔG = -65.70）

```
                 10         20         30          40         50
                GCU   -          A      U           GA     G   GA    GC
   UUUAAGAGGUG    GAC  AGAGUCAA  UUUUG  CUCGUGAGUU   UGAA  UC   UGG   C
   AAAUUCUCCAC    CUG  UCUCAGUU  AAAAC  GAGUACUCAG   ACUU  AG   ACC   U
                AGU   G          C      C           --     G  --     AC
        110           100        90         80         70         60
```

sRNA (5' to 3' orientation) mapped to this predicted precursor hairpin:

in the NC library:

AGAGGUGGCUGACAGAGUCAA (sequencing frequency: 11)

in the WC library:

AGAGGUGGCUGACAGAGUCAA (sequencing frequency: 23)

M35 （ΔG = -48.89）

```
            10              20           30          40
          UU  C                      CG     AU       AC
  ACUACU    C  CCGAUCCAUAAUAAGUGU   GUG   CUAGU    \
  UGAUGA    G  GGCUAGGUGUUAUUCACA   CAU   GAUUA    A
          GG  A                      --     --       AA
     80          70           60           50
```

sRNA (5' to 3' orientation) mapped to this predicted precursor hairpin:

in the NC library:

CACUUAUUGUGGAUCGGAGGG (sequencing frequency: 5)

in the WC library:

CACUUAUUGUGGAUCGGAGGG (sequencing frequency: 4)

M36 （ΔG = -75.79）

```
        10        20        30        40        50        60        70        80        90
            UGUCCAAA               G     G                    CAA         CACUA  UUCAUA   G
CACGCAUUUCGA        UUUGAUCAUCAAUUA ACUAA AAAAUGUGAAUUAUGUGUUA   AAAUUAUAC     GA      UUU A
GUGCGUAAAGUU        AAACUGGUAGUUAAU UGGUU UUUUACACUUAAUACAUAAU   UUUAAUAUG     CU      AAG A
            UUAAAAUG               G     G                    AUC         ACAGC  UUG---   A
        170       160       150       140       130       120       110       100
```

sRNA (5' to 3' orientation) mapped to this predicted precursor hairpin:

in the NC library:

UUUUGUUGGUGUAAUUGAUGG (sequencing frequency: 12)

in the WC library:

UUUUGUUGGUGUAAUUGAUGG (sequencing frequency: 23)

M37 （ΔG = -72.20）

```
       10        20        30        40        50
        CU                A       A     C    U      UUUGG
CCCAGCUU  UGGAGAAGCCGCAUCC AAAUUUG UUAGA UUCC AAAUAG     \
GGGUCGAA  ACCUCUUCGGUGUAGG UUUAAAU GAUCU AAGG UUUAUC     C
        AC                G       C     A    U      UAAUC
    110       100        90        80        70        60
```

sRNA (5' to 3' orientation) mapped to this predicted precursor hairpin:

in the NC library:

UAGCUAAAUUUGGGAUGUGGC (sequencing frequency: 7)

in the WC library:

UAGCUAAAUUUGGGAUGUGGC (sequencing frequency: 6)

M38 （ΔG = -52.50）

```
        10        20        30        40        50        60
        CG AG    CUU       C          AA    A      UU   CAA     UA
GUGACCCU  G  UAUC   UGACCCU CUUAGUUCAA  AAAU AAUUAG  UUU   AAAGU  U
CACUGGGA  C  AUGG   ACUGGGA GAAUCAAGUU  UUUA UUGAUU  AAA   UUUUA  A
        AU CU    CGU       A          GG    C      UU   A--     UU
130       120       110       100        90        80          70
```

sRNA (5' to 3' orientation) mapped to this predicted precursor hairpin:

in the NC library:

UUUGGUUGAACUAAGAAGGGU (sequencing frequency: 9)

in the WC library:

UUUGGUUGAACUAAGAAGGGU (sequencing frequency: 5)

M39 （ΔG = -122.90）

```
        10        20        30        40         50        60        70        80          90
                  C             A  UG   --          A  A         C    U  CUG           --    UUUC
 UGCAUGCGUAAAGAUCGG AAUUGCCCAUGUA AA  ACC  UCAUUCGUGGAU GC UCGCCCAUU AAAC GA   GAUUCAGUUUU   GAAU     \
 ACGUACGCAUUUCUAGCC UUGACGGGUACAU UU  UGG  AGUAAGCGCCUA CG AGCGGGUAA UUUG CU   CUAAGUCAAAG   UUUG     U
                  U             G  GU   AG          C  C         C    C  AAG           GU    UUUU
      190       180       170       160       150       140       130       120       110       100
```

sRNA (5' to 3' orientation) mapped to this predicted precursor hairpin:

in the NC library:

UACAUGGGCAGUUUCCGAUCU (sequencing frequency: 3)

in the WC library:

UACAUGGGCAGUUUCCGAUCU (sequencing frequency: 9)

M40 （ΔG = -50.89）

```
    10        20        30        40
                             C    AAAAUCC
 CGUCCUAAAAUAAGUGACGUGGAUUUG AUG        A
 GCAGGGUUUUAUUCACUGCACCUAAAC UAU        U
                             A    CUAAACA
    80          70        60          50
```

sRNA (5' to 3' orientation) mapped to this predicted precursor hairpin:

in the NC library:

CACGUCACUUAUUUUGGGACG (sequencing frequency: 4)

in the WC library:

CACGUCACUUAUUUUGGGACG (sequencing frequency: 3)

M41 （ΔG = -129.30）

```
                  10        20        30        40        50        60        70        80        90       100       110       120
                                          AAC  U   AC      A      A    U    U       A       AGA     CU   -     AU
 UACUCCCUCUGUUCCAAAAUGUAGGGCGUAUAACUUUUGUU   UG CAAA  UUUUAAA UUUGACC AGCUU  UAGC AAAAAUAUC AUAUUUAC  GUCAAA  AAGA UAUUA  UGAAA  \
 AUGAGGGAGGCAAGGUUUUACAUCCCGCGUAUUGAAAACAA   AC GUUU  AAGAUUU AAACUGG UUGAA  AUUG UUUUUAUAG UGUAGAUG  CGGUUU  UUCU GUAAU  ACUUU  A
                                          CUA  U   GA      C      -    C    U       C       CCA     AC   A     AU
 .    250       240       230       220       210       200       190       180       170       160       150       140       130
```

sRNA (5' to 3' orientation) mapped to this predicted precursor hairpin:

in the NC library:

UCUGUUCCAAAAUGUAGGGCG (sequencing frequency: 4)

in the WC library:

UCUGUUCCAAAAUGUAGGGCG (sequencing frequency: 15)

M42 （$\Delta G = -68.60$）

```
          10        20        30        40        50        60
      C-      C   C              C         CA  A  CU    A    G   CU
UUGGUG  UUCUAG UCU UAAAAAGCACUUCU CCGUUUACA  UG AG  GAGA GUAC UCC   \
AACCAC  AAGAUC AGA AUUUUUCGUGAAGA GGCAAAUGU  AC UC  UUCU CGUG AGG   C
      AA      A   A              A         CC  G  CU    -    G   AG
 130       120       110       100        90        80         70
```

sRNA (5' to 3' orientation) mapped to this predicted precursor hairpin:

in the NC library:

GUAAACGGAAGAAGUGCUUUU (sequencing frequency: 21)

in the WC library:

GUAAACGGAAGAAGUGCUUUU (sequencing frequency: 9)

M43 （$\Delta G = -43.90$）

```
          10          20          30            40
        CU  A           A  .UA     GG  AAAAU
CUCUAGC  AG AUACCCAUAU UG   UUGGG  GC      A
GAGGUCG  UC UAUGGGUAUA AC   AACCC  CG      U
        -G  G           A  \--     AU  AUGGG
   120         110          100          90      70
                                                       50
                                                         A
                                                     UUGG U
                                                     AACC A
                                                         U
```

sRNA (5' to 3' orientation) mapped to this predicted precursor hairpin:

in the NC library:

AAUAUGGGUAUGCUGGCUGGA (sequencing frequency: 6)

in the WC library:

AAUAUGGGUAUGCUGGCUGGA (sequencing frequency: 7)

M44 （$\Delta G = -51.30$）

```
          10        20        30        40
   G          A            A        --     UAA
AGC UACCUAGCCU AAAUAUUUAAAA UAAUUGGA  GCAAC   \
UCG GUGGAUUGGA UUUAUGGAUUUU AUUAACUU  UGUUG   A
   -          C            C        GU     UUG
```

```
          90          80          70          60          50
```

sRNA (5' to 3' orientation) mapped to this predicted precursor hairpin:

in the NC library:

UUAGGUAUUUCAGGUUAGGUG (sequencing frequency: 103)

UAGCCUAAAAUAUUUAAAAAUAA (sequencing frequency: 14)

UUAGGUAUUUCAGGUUAGGU (sequencing frequency: 1)

UAGGUAUUUCAGGUUAG (sequencing frequency: 1)

in the WC library:

UUAGGUAUUUCAGGUUAGGUG (sequencing frequency: 131)

UUAGGUAUUUCAGGUUAGGU (sequencing frequency: 1)

M45 （ΔG = -120.78）

```
       10        20        30        40        50         60        70        80        90        100       110       120
          C                         CC   G   UCGAACU     C        C           U        AA         C        CUA  UU    G
AGUACUCCCUC AUUCCAAAAUAUAACGUGCCUACG  UUUC AGG       UUGAC AUCAAUUA ACCAACAAAAU UGAAUUAU  GUUAUAAAA UUAUACCA  GA   UUU A
UCAUGAGGGAG UAAGGUUUUAUAUUGCACGGGUGC  AAAG UCC       AACUG UAGUUAAU UGGUUGUUUUA ACUUAAUA  UAAUAUUUU AAUAUGGU  CU   AAG A
          A                         AU   -   UAAAACG     U        C           C        CA         C        AAC  UUG   A
   240       230       220       210        200       190       180       170       160       150       140       130
```

sRNA (5' to 3' orientation) mapped to this predicted precursor hairpin:

in the NC library:

AUUUUGGAAUAGAGGGAGUAC (sequencing frequency: 4)

in the WC library:

AUUUUGGAAUAGAGGGAGUAC (sequencing frequency: 7)

M46 （ΔG = -146.60）

```
        10        20        30        40        50            60        70        80        90        100
             U    CA             A         A      GU  --     GUUCGAGUAGA     CC        CGA   A   AUU  C   GAC
 CAAAUGGCCAGA UCGGG  AAAUUCGGUCAUG UGGCAUGUG CGUGG  C   GGGCC           CGCCG  GAACCCAU  CGG GCC  CA AUG   \
 GUUUACCGGUUU AGUCU  UUUAAGCCGGUGC ACCGUACAC GCACC  G   CCUGG           GCGGC  CUUGGGUG  GCC CGG  GU UGC   A
             U    AC             G         A      UG  UG     UAGCGUAGCGU      CA        AAA   A  CC-  A   ACC
    210        200       190       180       170       160       150        140       130        120       110
```

sRNA (5' to 3' orientation) mapped to this predicted precursor hairpin:

in the NC library:

UCGGUCAUGAUGGCAUGUGAC (sequencing frequency: 26)

in the WC library:

UCGGUCAUGAUGGCAUGUGAC (sequencing frequency: 31)

M47 （ΔG = -39.20）

```
          10          20
        GA             AC     AU
 UGCAUGU  UCUCUCCCAAC.    CGG   \
 AUGUACG  GGAGAGGGUUG\     GUC   U
        G-             --      AA
    90         80          30
                                 40          50
                       UAUUCA   -        A   AU
                             UG  AGUACU  CU   U
                             AC  UCAUGA  GA   A
                       CUAUCA   A        -   AU
                              70        60
```

sRNA (5' to 3' orientation) mapped to this predicted precursor hairpin:

in the NC library:

UGCAUGUGAUCUCUCCCAAC (sequencing frequency: 19)

in the WC library:

UGCAUGUGAUCUCUCCCAAC (sequencing frequency: 40)

M48 （ΔG = -98.40）

```
                10              20             30
                                G          C          AG
     UCAUCAGUGACGGGCGUG  UACGUCCG  CACUGAUG   G
     AGUAGUCACUGCCCGCAU  GUGCGGGC  GUGACUAC   G
                                G          A          UU
   70           60           50           40
```

sRNA (5' to 3' orientation) mapped to this predicted precursor hairpin:

in the NC library:

UCAUCAGUGACGGGCGUGGUA (sequencing frequency: 5)

in the WC library:

UCAUCAGUGACGGGCGUGGUA (sequencing frequency: 9)

M49 （ΔG = -112.00）

```
        10          20          30          40            50          60
                                            A                 CAGGCAG
GGGCCCACAUGUCAGGCGAGGACGUGGUGUUGCAAUCU  ACAGAAAUCUUGCA         A
CCCGGGUGUGCAGUCCGCUCCUGCACCGCAACGUUAGA  UGUCUUUAGGACGU         G
                                            A                 AGGAGAG
        180         170         160         150               70
```

```
                          80       90       100      110
                                 GAG      GC          AA   CAAG
                                    AGGAUG  UGGACCGUGA  UG     \
                                    UCUUGU  AUUUGGCGCU  AC     A
                                 UCA      --          AC   UCAG
                                  140        130      120
```

sRNA (5' to 3' orientation) mapped to this predicted precursor hairpin:

in the NC library:

UGUAAGAUUGCAACGCCACGU (sequencing frequency: 11)

in the WC library:

UGUAAGAUUGCAACGCCACGU (sequencing frequency: 4)

M50 （ΔG = -66.80）

```
         10        20        30        40
                              C     U       AG
GUACUCCCUCCGUCCCAAAAUAAGUGA GUGGA UUGUAUA   A
CAUGAGGGAGGCAGGGUUUUGUUCACU CACCU AACAUAU   A
                              A     U       CU
        80        70        60        50
```

sRNA (5' to 3' orientation) mapped to this predicted precursor hairpin:

in the NC library:

ACUUGUUUUGGGACGGAGGGA (sequencing frequency: 22)

in the WC library:

ACUUGUUUUGGGACGGAGGGA (sequencing frequency: 4)

M51 （ΔG = -37.50）

```
         10        20        30           40
          C      U          CGCUCGUGGG  A  UAG-      G
GUACUUCUU CGAUCC UAAAAAAUGUC          UC CU    UACAA \
CAUGAGGGA GUUAGG AUUUUUUACAG          AG GA    AUGUU A
          A      U          CAAAUAAAAU  -  UACA      U
100        90        80        70         60
```

sRNA (5' to 3' orientation) mapped to this predicted precursor hairpin:

in the NC library:

UUUAUGGAUUGAAGGGAGUAC (sequencing frequency: 11)

in the WC library:

UUUAUGGAUUGAAGGGAGUAC (sequencing frequency: 24)

M52 （ΔG = -61.90）

```
          10        20        30        40
       U     A                 C       G
ACUACG AUAAG UGAUCAUACAGUCUGUUC ACUUUUU A
UGAUGC UAUUC ACUAGUAUGUCAGACAAG UGAAAGG A
       C     G                 C       G
      80        70        60        50
```

sRNA (5' to 3' orientation) mapped to this predicted precursor hairpin:

in the NC library:

UGUAUGAUCAGCUUAUCCGUA (sequencing frequency: 8)

in the WC library:

UGUAUGAUCAGCUUAUCCGUA (sequencing frequency: 5)

M53 （ΔG = -100.10）

```
          10         20         30         40
   -   U                       AA    A    A
AC CCC CCGUUCCAUAUUAAUUGGCGCG  UUUA UACA A
UG GGG GGCAAGGUAUAAUUAACCGUGC  AAAU AUGU U
   A   U                       CA    C    U
         70          60            50
```

sRNA (5' to 3' orientation) mapped to this predicted precursor hairpin:

in the NC library:

UUAAUAUGGAACGGUGGGAGU (sequencing frequency: 4)

in the WC library:

UUAAUAUGGAACGGUGGGAGU (sequencing frequency: 5)

M54 （ΔG = -138.6）

```
        10       20       30       40               50
             UA     U      UA   A           AACA   CC
GCUGGUUCGGGCCA  GAGAU GAGGG  UG  GCGCGCCCGC      GC  C
CGACUAAGCCCGGU  CUCUA CUCCC  GC  CGCGCGGGCG      CG  U
             UC     C      CC   C           UACA   --
 270     260      250      240      230       200     190
                                                            70       80        90      100
```

```
CA  A  UCCU     U    U   AGA
  GCG GC   CGUCGC  GGCCUC GCC   A
  CGC CG   GCAGCG  UCGGAG CGG   U
AA  G  CGCC     G    G  CCU
       180            110
```

sRNA (5' to 3' orientation) mapped to this predicted precursor hairpin:

in the NC library:

CGGGCCAUAGAGAUUGAGGGU (sequencing frequency: 3)

in the WC library:

CGGGCCAUAGAGAUUGAGGGU (sequencing frequency: 7)

M55 （ΔG = -119.30）

```
       10        20        30        40        50        60        70        80
      AA       C                      G       A                G    ACACAUGAA   G
UAAAAA  CACUUCU CCGUUUACGCAUGAAGCUGAAAA UAUGUCC GAGGUGUUUCCUGCAG UUU          GCU A
GUUUUU  GUGAAGA GGCAAAUGUGUACUUCGACUUUU GUGCAGG CUCCACGAAGGGCGUC AAA          CGA U
      C-       A                      G       C                G    UAGCUGAUC   A
     160        150       140       130       120       110       100       90
```

sRNA (5' to 3' orientation) mapped to this predicted precursor hairpin:

in the NC library:

UGUGUAAACGGAAGAAGUGCU (sequencing frequency: 43)

in the WC library:

UGUGUAAACGGAAGAAGUGCU (sequencing frequency: 21)

M56 （ΔG = −68.70）

```
          10        20        30        40        50          60        70
    U    ACGC            A                U           U  C--   UCUA   CAU
AUAC UAAA    UUAGAGAUGGGC AAGUGCUAUCAAUUGU UGAACAAACUU GC   CAG    CGA    \
UAUG AUUU    AAUCUCUACCCG UUCACGAUAGUUGGCA ACUUGUUUGAG CG   GUC    GCU    U
    C    GACA            A                U           U  UUA   CAA-   CAG
    140       130       120       110       100         90        80
```

sRNA (5' to 3' orientation) mapped to this predicted precursor hairpin:

in the NC library:

UGUUCAUACGGUUGAUAGCAC (sequencing frequency: 3)

in the WC library:

UGUUCAUACGGUUGAUAGCAC (sequencing frequency: 22)

M57 （ΔG＝−52. 60）

```
          10          20         30         40         50
          10          20         30         40         50
    G                                     AAA     AU     UCA
 CC  UCCAACCCUUCUUAGUUCAAGCAAAUGGAUUA    UUUUA   CACA     \
 GG  AGGUUGGGAGGAAUCGAGUUUGUUUACUUGAU    AAAAU   GUGU     G
   -                                      C--     --     UUU
 100        90         80         70          60
```

sRNA (5' to 3' orientation) mapped to this predicted precursor hairpin:

in the NC library:

UUGAGCUAAGGAGGGUUGGAG (sequencing frequency: 37)

in the WC library:

UUGAGCUAAGGAGGGUUGGAG (sequencing frequency: 6)

M58 （ΔG = -58.80）

```
              10         20          30         40
      C   ACC        -                          C    U
  UACU  CC    GAUCCAU  AUAAGUGUCGGGGAUUUAGUACAAA UUG \
  AUGA  GG    CUAGGUA  UAUUUACAGCCCCUAAAUCAUGUUU AAU  A
      U   ACA        U^                         C    C
          90         80          70         60          50
```

sRNA (5' to 3' orientation) mapped to this predicted precursor hairpin:

in the NC library:

GAUCCAUAUAAGUGUCGGGGA (sequencing frequency: 7)

in Uhe WC library:

GAUCCAUAUAAGUGUCGGGGA (sequencing frequency: 6)

M59 （ΔG = -68.90）

```
          10         20          30         40
              A  A            GGGG-      A   GUG
 CUGAUACGUGC  UU  AAGACCACUUU      AGAUAC UUA    \
 GACUAUGUACG  AA  UUCUGGUGAAA      UCUAUG GGU    A
              G  C            ACGCG      A   ACA
          80         70         60        50
```

sRNA (5' to 3' orientation) mapped to this predicted precursor hairpin:

in the NC library:

UGGUCUUCAAGGCAUGUAUCA (sequencing frequency: 13)

in the WC library:

UGGUCUUCAAGGCAUGUAUCA (sequencing frequency: 9)

M60（ΔG = -57.50）

```
         10         20             30        40
                      G        A   A--      AA
  UUAUGCCCAUAAGAUUGUU UAUGUCGC GGA    UUUG   A
  AAUACGGGUAUUCUGACAA AUACAGCG CCU    AAAC   A
                      A        A   CAA      CC
    80        70        60         50
```

sRNA (5' to 3' orientation) mapped to this predicted precursor hairpin:

in the NC library:

UUAUGCCCAUAAGAUUGUUGUA (sequencing frequency: 10)

in Uhe WC library:

UUAUGCCCAUAAGAUUGUUGUA (sequencing frequency: 34)

UAAAACAGUCUUAUGGGCAUA (sequencing frequency: 4)

M61（ΔG = -44.50）

```
         10        20          30        40        50
     A   C          G      A       UAC         GACG   U
UACUC CUC GAUCCUAAAUU UUGUC  AAAUAU  AUGUAUCUA    CUU \
AUGAG GAG CUAGGAUUUAA AACAG\ UUUAUA  UACAUAGAU    GAA U
     G   U          G      G       CC-         AA--   U
     110       100                   70        60
                             80
                              GGA  A
                                CA A
                                GU C
                              AGA  U
                             90
```

sRNA (5' to 3' orientation) mapped to this predicted precursor hairpin:

in the NC library:

AGAAUUUAGGAUCUGAGGGAG (sequencing frequency: 7)

in the WC library:

AGAAUUUAGGAUCUGAGGGAG (sequencing frequency: 5)

M62（ΔG = -79.30）

```
          10         20        30            40             50
    U                                  --            C--        GGU
GAAUU UGUGAUCUUGAAGGGCAAUGUGGUUGGA  UGGUGCAGAAG.     GUAAGAU    G
CUUAA ACACUAGAACUUCCUGUUAUACCAACCU  ACCACGUCUUU\     UAUUUUA    A
```

```
      U                            AC             UAG         AGG
160         150         140         130         120                 70          60
                                                               80          90
                                                              CUUUGA                GG
                                                                     AAUAUUUGAAU   C
                                                                     UUAUAAACUUA   A
                                                              UUGUGG                AA
                                                                   110         100
```

sRNA (5' to 3' orientation) mapped to this predicted precursor hairpin:

in the NC library:

UUUGUGAUCUUGAAGGGCAAU (sequencing frequency: 4)

in the WC library:

UUUGUGAUCUUGAAGGGCAAU (sequencing frequency: 7)

M63（ΔG = -54.40）

```
                         10             20             30             40
                      U    UU                   GGA              ACU     GUA
               GUUAAG  UA   UACUCCCUCCGGCC     AUUACUUGUC     GAU      \
               CGGUUU  AU   AUGAGGGAGGCUGG     UAAUGAAUAG     CUA      C
                      U    UC                   GGA              GGU     AAU
                   90             80             70             60          50
```

sRNA (5' to 3' orientation) mapped to this predicted precursor hairpin:

in the NC library:

AGUAAUAGGGGUCGGAGGGAG (sequencing frequency: 16)

in Uhe WC library:

AGUAAUAGGGGUCGGAGGGAG (sequencing frequency: 9)

M64（ΔG = -51.40）

```
              10              20              30              40              50              60
   A     A     AUAU                    A    G                             C    G       A    A    UG
GU  AUA  AAU       GUACUUCCUCUG  UC  AUAAAGAGUGUUGUCCA  UU  GUAC  AA  UU    U
CG  UGU  UUG       UAUGAGGGAGGC  AG  UAUUUUUCACAACGGGU  AA  CAUG  UU  AG    A
   G     A     CUU-                    C    G                             A    A       A    C    UU
          120              110             100              90              80              70
```

sRNA (5' to 3' orientation) mapped to this predicted precursor hairpin:

in the NC library:

AACACUUUUUAUGGACCGGAG (sequencing frequency: 4)

in the WC library:

AACACUUUUUAUGGACCGGAG (sequencing frequency: 8)

M65（ΔG＝−39. 20）

```
         10        20        30        40
                    A   AUC  CA          A
UACUCCCUCUGUUUCUAAGU CUU   GU  UUUUAGUUCA A
AUGAGGGAGGUAAGGAUUUA GAA   CA  AGAAUCAAGU U
                    A   CGA  CC          U
       80        70        60        50
```

sRNA (5' to 3' orientation) mapped to this predicted precursor hairpin:

in the NC library:

AAGAAUUUAGGAAUGGAGGGA (sequencing frequency: 36)

in the WC library:

AAGAAUUUAGGAAUGGAGGGA (sequencing frequency: 6)

M66（ΔG＝-75.44）

```
         10         20        30        40        50
   C     C                                C   AACUUU
 UA UACUC CUCCGAUCCAUAACAAGUGUCGCUGGUUUAGUA GAA      G
 AU AUGAG GAGGCUAGGUAUUGUUCACAGCGACUAAAUCAU CUU      U
   C     A                                U   CAAUCA
        100       90        80       70      60
```

sRNA (5' to 3' orientation) mapped to this predicted precursor hairpin:

in the NC library:

AGCGACACUUGUUAUGGAUCG (sequencing frequency: 3)

in the WC library:

AGCGACACUUGUUAUGGAUCG (sequencing frequency: 6)

M67（ΔG＝-79.00）

```
         10         20        30        40        50
     G         G       GGCG             ACUC    G  AC
 AUGU CGCCCGUUA UGAUGA    UCAUCAGUGACAGG    AUCA UG  \
 UACG GCGGGCAGU ACUACU    AGUAGUCACUGUCC    UGGU GC  G
     A         G       GGGG             GCA-    -  UG
         100       90        80       70         60
```

sRNA (5' to 3' orientation) mapped to this predicted precursor hairpin:

in the NC library:

GUCAUCAGUGACGGGCGAGCA (sequencing frequency: 5)

in the WC library:

GUCAUCAGUGACGGGCGAGCA (sequencing frequency: 7)

M68（ΔG = -69.60）

```
         10        20        30        40        50            60               70
    C                        A    U   U  GG   GUGU     -----       AGAUAAG    GGG
UGGA CAUUGGAUCUAAAACGUGUGGUGCA AUUA AGG UG  AA      UAUC     AAGCAUA       UACA   G
ACUU GUAACCUAGAUUUUGCACACCACGU UAGU UCC AC  UU      GUGG     UUUGUGU       GUGU   G
    A                        C    U   -  UU   AGUU    GUGAA       AAAAA--    AUU
    150       140       130        120       110       100          90         80
```

sRNA (5' to 3' orientation) mapped to this predicted precursor hairpin:

in the NC library:

UGGAUCUAAAACGUGUGGUGC (sequencing frequency: 12)

in the WC library:

UGGAUCUAAAACGUGUGGUGC (sequencing frequency: 8)

M69（ΔG = -126.60）

```
       10        20        30        40        50        60        70        80         90          100
                  G                       ACA     A    G     C           UUCAU-                 --      AG
CCCUCCGUCCCAUUAUAU AGGCACGCACGCGUCCCAAGAUC   AAUUU ACUA CAAAA AUAAAUUAUAU      AAAAAUAUAUCAUUAGA   UUCGU  U
GGGAGGCAGGGUGAUAUG UCCGUGCGUGCGCAGGGUUCUAG   UUAAA UGAU GUUUU UAUUUAAUAUA      UUUUUAUAUAGUAAUUU   AAGUA  C
                  A                       CAG     C    G     A           CUGCAU                 UU      AA
     210       200       190       180       170       160       150       140       130       120       110
```

sRNA (5' to 3' orientation) mapped to this predicted precursor hairpin:

in the NC library:

UGCCUAGUAUAGUGGGACGGA (sequencing frequency: 29)

in the WC library:

UGCCUAGUAUAGUGGGACGGA (sequencing frequency: 23)

M70（ΔG = -57.10）

```
          10        20        30         40
        U  C           C      U      GC
CUCUUUGC AU UUCCACAGCUUU UUGAAC GCAUCU  \
GAGAGACG UA AGGGUGUCGAAA AACUUG CGUAGG  A
        U  A           U      -      UA
120      110       100       90          50
                                         60        70
```

```
UACUU     A    UU
     GCUU UGC   A
     UAGG ACG   A
UCG--     A    UC
          80
```

sRNA (5' to 3' orientation) mapped to this predicted precursor hairpin:

in the NC library:

GUUCAAUAAAGCUGUGGGAAA (sequencing frequency: 10)

in the WC library:

GUUCAAUAAAGCUGUGGGAAA (sequencing frequency: 5)

M71（ΔG = −86. 50）

```
        10        20        30        40        50        60             70        80
        U             G   UA        GA                UG          --AA            UAGUAA
CAUUAGAUC UUAAAUAAACGGAU AGA  AAAGGUG  ACCUCUAAUCACUUAAA  CAUUUUAUGA     AAAAAAACAC      \
GUAAUCUAG AGUUUAUUUGCCUA UCU  UUUCCAC  UGGAGAUUAGUGAAUUU  GUAAAAUACU     UUUUUUUGUG      A
        -             A   UC        UC                UC          CUUC           CACCCU
    170        160       150       140       130       120       110       100       90
```

sRNA (5' to 3' orientation) mapped to this predicted precursor hairpin:

in the NC library:

UAGAUCUUUAAAUAAACGGAUG (sequencing frequency: 4)

in the WC library:

UAGAUCUUUAAAUAAACGGAUG (sequencing frequency: 22)

M72（ΔG = -67.30）

```
         10        20        30        40        50        60
      C    C     C          U    A      UC   GCA       G
 AUCCCG GAAA UCUCG UUUUAGUUCA UUUU AAAUCU  UAU   AAAUUUU U
 UAGGGC CUUU AGGGC AAGAUCAAGU AAGA UUUAGA  AUA   UUUAAGA U
      A    C     A          U    G      GA   AG-       C
         110        100      90       80        70
```

sRNA (5' to 3' orientation) mapped to this predicted precursor hairpin:

in the NC library:

UGAGAAUUGAACUAGAAACGG (sequencing frequency: 5)

in the WC library:

UGAGAAUUGAACUAGAAACGG (sequencing frequency: 4)

M73（ΔG = -58.50）

```
           10                20             30             40
                A                C      -         C      UUC
 UAAAGCCUUAG GAGUAUCUACA UGGU AGUAUU UUCU    U
 AUUUCGGAAUC CUCAUAGAUGU ACCG UCAUGA AAGG    U
                C                C      U         A      UGG
                  80             70             60           50
```

sRNA (5' to 3' orientation) mapped to this predicted precursor hairpin:

in the NC library:

UGUAGAUACUCCCUAAGGCUU (sequencing frequency: 677)

CUUAGAGAGUAUCUACACUGGU (sequencing frequency: 36)

AGAUACUCCCUAAGGCUU (sequencing frequency: 1)

in the WC library:

UGUAGAUACUCCCUAAGGCUU (sequencing frequency: 456)

GUAGAUACUCCCUAAGGCU (sequencing frequency: 1)

M74（ΔG = -59.04）

```
         10          20          30          40          50          60
                           A       C UG C   C    CCAU         -   GACUAG
 CUUCUCGUAUAUGCGGAUGUACC UAUGGA C   A UU AAG     GGUUCGG UC        A
 GAAGAGCAUAUAUGUCUACGUGG AUACUU G   U AA UUC     CCGAGCC GG        C
                           C       A GU U   A    UU--         C   ACUACG
      120         110         100        90         80            70
```

sRNA (5' to 3' orientation) mapped to this predicted precursor hairpin:

in the NC library:

UCUCGUAUAUGCGGAUGUACC (sequencing frequency: 13)

in the WC library:

UCUCGUAUAUGCGGAUGUACC (sequencing frequency: 19)

M75（ΔG = -111.50）

```
         10          20          30          40          50          60              70
     C       U        A          GU                C    C         C  U--      ACC
GUCCG CCCCGU UCCGGUC GCCCCCUUC  CGGUCCUCCUCCGU GCC GUCCGACU UG    CGUA    G
CAGGC GGGGUA AGGUCAG CGGGGGAAG  GCCAGGAGGAGGCA CGG CAGGCUGA GC    GUAU    G
     C       C        G          UG                U    C         C  CCC      CCC
    140         130         120        110         100          90         80
```

sRNA (5' to 3' orientation) mapped to this predicted precursor hairpin:

in the NC library:

AGGACCGGUGAAGGGGGCGGA (sequencing frequency: 30)

in the WC library:

AGGACCGGUGAAGGGGGCGGA (sequencing frequency: 57)

M76（ΔG = -68.20）

```
          10         20         30         40
                      A          C               AG
  UACUCUCUCCGUCCCAA AUAAGUGA GUGGAUUUGUAUA   A
  AUGAGAGAGGCAGGGUU UAUUCACU CACCUAAACAUAU   A
                      A          U               CU
           80         70         60         50
```

sRNA (5' to 3' orientation) mapped to this predicted precursor hairpin:

in the NC library:

ACUUAUAUUGGGACGGAGAGA (sequencing frequency: 5)

in the WC library:

ACUUAUAUUGGGACGGAGAGA (sequencing frequency: 6)

M77（ΔG = -59.60）

```
         10                 20         30         40
     C             C          G         C    UCC
 CACU UGAUCAUUUGCC CGUCUUGUA CACUGACA GUGG   A
 GUGA ACUAGUAAACGG GCGGGACAU GUGACUGU CACC   U
     A             A          A         A    CCG
  90        80         70         60         50
```

sRNA (5' to 3' orientation) mapped to this predicted precursor hairpin:

in the NC library:

AGGGCGAGGCAAAUGAUCAAA (sequencing frequency: 33)

in the WC library:

AGGGCGAGGCAAAUGAUCAAA (sequencing frequency: 6)

M78（ΔG = -69.10）

```
          10          20         30         40           50
             A     U   A                 A  AG
 CUCUAAAAAG AUUUC CCC UUUACACAUGAAG.CUG GA  C
 GAGAUUUUUC UGAAG GGG AAAUGUGUACUUC GGC CU  A
```

```
          G      U   C                 \   -  GC
      130         120         110         100
                                              60          70
                                          AAA        CC    A
                                             UGCUUCC   UAG \
                                             ACGAAGG   GUC A
                                          C--        AC    G
                                               90          80
```

sRNA (5' to 3' orientation) mapped to this predicted precursor hairpin:

in the NC library:

UGUGUAAACGGGUGAAGUGCU (sequencing frequency: 17)

in the WC library:

UGUGUAAACGGGUGAAGUGCU (sequencing frequency: 13)

M79（ΔG = −110. 30）

```
        10        20        30        40        50        60        70        80         90        100
                   A             CUAUAAA         C   C   C            -    A      A     A  -      G  C   AG
  UUUUUUUCGGCUUCU GGACCGGCUUCUC       CUGCUCUCAC UAG UUC UGGAGAAGCCGC AUC  AGAUUU GUUAG  CU CCAAA UA UUU   \
  AAAAAAAGCCGAAGG CCUGGCCGAAGAG       GACGGGAGUG GUC AAG ACCUCUUCGGCG UGG  UUUAAA UAAUC  GA GGUUU AU AAA   A
                   A             ACUCUUC         A   A   U            G    A      C     C  C      G  U   CC
     200        190       180       170       160       150       140       130       120        110
```

sRNA (5' to 3' orientation) mapped to this predicted precursor hairpin:

in the NC library:

UUUCGGCUUCUAGGACCGGCU (sequencing frequency: 15)

in the WC library:

UUUCGGCUUCUAGGACCGGCU (sequencing frequency: 3)

M80（ΔG = -82.20）

```
             10         20         30         40         50         60
                                AA         U            AAA      C   U
  ACCAUUAGGGUACCCUCCGGCCCU  UUAGUUCAA  CAAAUGAACUA   UUCCAA  AAA  C
  UGGUAAUCCCAUGGGAGGUUGGGA  AAUCAAGUU  GUUUACUUGAU   AAGGUU  UUU  A
                                GA         U            CAA      -   U
        120          110        100      90       80        70
```

sRNA (5' to 3' orientation) mapped to this predicted precursor hairpin:

in the NC library:

UUGAACUAAAGAGGGUUGGAG (sequencing frequency: 8)

in the WC library:

UUGAACUAAAGAGGGUUGGAG (sequencing frequency: 5)

M81

super_1:16079568-16079588 **(ΔG = -56.50)**

```
           10        20        30        40        50        60
          -    CG  G       C          CA    A     AG-        UGAC
UGACCCUCA GGUA  CU UGACCCU CUUAGUUCAA  AAAU AACUA    UUUUAAAA    A
ACUGGGAGU CCAU  GA ACUGGGA GAAUCAAGUU  UUUA UUGAU    GAAAUUUU    C
          C    AG  A       A          UG    C     CAA        UCAA
        120       110       100        90        80        70
```

sRNA (5' to 3' orientation) mapped to this predicted precursor hairpin:

in the NC library:

UUUGAACUAAGAAGGGUCAAA (sequencing frequency: 43)

in the WC library:

UUUGAACUAAGAAGGGUCAAA (sequencing frequency: 5)

super_3:15856277-15856297 **(ΔG = -66.70)**

```
         10          20        30        40        50        60
   AC         -  C         C          C           AAA        UGAC
GUG  CCUCAGGGU AC UUUGACCCU CUUAGUUCAA CAAAUGAACUA   UUUUAAAA    A
CAC  GGAGUCCCA UG AAACUGGGA GAAUCAAGUU GUUUACUUGAU   AAAGUUUU    U
   CA         U  U         A          U           CAA        UCAA
  130         120       110      100      90      80        70
```

sRNA (5' to 3' orientation) mapped to this predicted precursor hairpin:

in the NC library:

UUUGAACUAAGAAGGGUCAAA (sequencing frequency: 43)

in the WC library:

UUUGAACUAAGAAGGGUCAAA (sequencing frequency: 5)

super_7:14469193-14469213 **(ΔG = -75.70)**

```
         10        20        30        40        50        60
                   C          CCA     C           AAG        UGAC
GUGACCCUCAGGGUACC UUUGACCCU    UAGUUCAA CAAAUGAACUA   UUUUAAAA    A
CACUGGGAGUUCCAUGG AAACUGGGA    AUCAAGUU GUUUACUUGAU   AAAGUUUU    C
                   A          AGA     U           CAA        UUAA
  130        120        110       100        90        80        70
```

sRNA (5' to 3' orientation) mapped to this predicted precursor hairpin:

in the NC library:

UUUGAACUAAGAAGGGUCAAA (sequencing frequency: 43)

in the WC library:

UUUGAACUAAGAAGGGUCAAA (sequencing frequency: 5)

M82（ΔG = -44.10）

```
        10        20        30        40        50
            AUC          C        C  AAA        UGGCA
    CUUUGACC   UUUAGUUCAA CAAAUGAA UA   UUUCAAAA     \
    GAAACUGG   GAAUCAAGUU GUUUACUU AU   AAAGUUUU     C
            GAU          U        A  CUA        UUUAA
     100        90        80        70        60
```

sRNA (5' to 3' orientation) mapped to this predicted precursor hairpin:

in the NC library:

UUGAACUAAGUAGGGUCAAAG (sequencing frequency: 9)

in the WC library:

UUGAACUAAGUAGGGUCAAAG (sequencing frequency: 8)

M83（ΔG = -79.53）

```
         10        20        30        40        50        60
         A                            G  A    U     UGUGACUU
 GGGUCGCC UUAGGGUACCCUCCAACACUCUUUAGUU AA CAAA GAACU        \
 CCCAGUGG AAUCCCAUGGGAGGUUGUGAGAAAUCAA UU GUUU CUUGA        U
         C                            G  A    U     UUUUAAGG
     130         120       110       100        90        80
```

sRNA (5' to 3' orientation) mapped to this predicted precursor hairpin:

in the NC library:

CUAAAGAGUGUUGGAGGGUAC (sequencing frequency: 10)

in the WC library:

CUAAAGAGUGUUGGAGGGUAC (sequencing frequency: 4)

M84（ΔG = -60.30）

```
         10        20        30        40
    A                 C     A           CAA
  AC ACCUCUGUUCCCAUAUA AAGAC UUCUGGUAGUU   \
  UG UGGAGACAAGGGUAUAU UUCUG AGGACCGUCAA   A
    A                 A     C           AUU
      80        70        60        50
```

sRNA (5' to 3' orientation) mapped to this predicted precursor hairpin:

in the NC library:

UAUAUAUGGGAACAGAGGUAG (sequencing frequency: 5)

in the WC library:

UAUAUAUGGGAACAGAGGUAG (sequencing frequency: 6)

M85（ΔG = -53.90）

```
       10        20        30        40        50         60
    UU  UC          A   C             CC     A  AC-     UG
GAUU  UU  UACUCCCUCC AUC AUAAUAAGUGUCU  GAUUU GU   AACUU  U
UUGA  AA  AUGAGGGAGG UAG UAUUAUUCACAGA  CUAAA CA   UUGAA  A
    UU  UU          C   C             AU     A  CGA     UC
         110       100        90        80        70
```

sRNA (5' to 3' orientation) mapped to this predicted precursor hairpin:

in the NC library:

CACUUAUUAUCGAUCGGAGGG (sequencing frequency: 4)

in the WC library:

CACUUAUUAUCGAUCGGAGGG (sequencing frequency: 7)

M86（ΔG = -108.70）

```
        10        20        30        40        50        60         70         80         90
     A   G        AC                    A         G     CGA   G  U        U      UU       CU
GGCUU GGC AGGGCCGC  GCUGCUAUUCGAUUUUCUCA GGGUUUUUU UGCAA   CGU GU CUCGGGCC CCCCGU  GGGGCAG  \
UCGAA CCG UCCUGGUG  CGACGAUGAGCUAAAAGAGU CUUAAAAAA ACGUU   GCG UA GGGCUCGG GGGGCG  CCCCGUU  G
     G   G        CC                    -         -     UUA   G  C        C      CU       CC
     180      170       160       150        140        130       120        110        100
```

sRNA (5' to 3' orientation) mapped to this predicted precursor hairpin:

in the NC library:

AAAAUCGAGUAGCAGCCCGUG (sequencing frequency: 144)

AAAAUCGAGUAGCAGCCCGU (sequencing frequency: 1)

in the WC library:

AAAAUCGAGUAGCAGCCCGUG (sequencing frequency: 28)

M87（ΔG = -60.90）

```
         10        20        30        40        50
             A        AAA    UCA      AG         G    GA
```

```
 UCUGUCCAACAAA  GAUGUCUC     UUUG     AAAUUU   AUGUAUUUA  ACAU   C
 AGGCAGGUUGUUU  CUACAGAG     AAAC     UUUAAA   UACGUAGAU  UGUG   U
               -         GUA     UGG        CU          A      AU
     110         100        90         80         70        60
```

sRNA (5' to 3' orientation) mapped to this predicted precursor hairpin:

in the NC library:

AGACAUCUUUGUUGGACGGA (sequencing frequency: 3)

in the WC library:

AGACAUCUUUGUUGGACGGA (sequencing frequency: 8)

M88（ΔG = -47.36）

```
        10          20         30          40
  UU        A--                      U      GAC
CG   UGUAAAU   GUACUCCCUCCGUCCCAUAU  UAAGU     \
GU   GUAUUUA   CAUGAGGGAGGCAGGGUAUG  GUUUA     U
  UU        CAA                      -      AAC
     120       110        100        50
                                             60         70
                                     CCCAAA         AUCUAUU
                                            UGGAUGU         \
                                            AUCUGCG         C
                                     UACAUA         AAAAACC
                                       90           80
```

sRNA (5' to 3' orientation) mapped to this predicted precursor hairpin:

in the NC library:

UGGGACGGAGGGAGUACAACA (sequencing frequency: 8)

in the WC library:

UGGGACGGAGGGAGUACAACA (sequencing frequency: 5)

M89（ΔG = -46.60）

```
         10          20           30          40
           C           AAAG-    C   CU      UA
 GUACUUCCUC  GAUCCAUAG      UGU  GC   AUUU    U
 CAUGAGGGAG  CUAGGUAUU      ACA  UG   UGAA    A
           A           GUUAA    -   AU      UC
 90         80          70         60          50
```

sRNA (5' to 3' orientation) mapped to this predicted precursor hairpin:

in the NC library:

GUUAUGGAUCAGAGGGAGUAC (sequencing frequency: 4)

in the WC library:

GUUAUGGAUCAGAGGGAGUAC (sequencing frequency: 4)

M90（ΔG＝--55. 24）

```
          10        20        30        40
 -         C            U    CCAA         A
  UACUCCCUC GACCCGUAUUAC UGUC    AUUUAGUACA A
  AUGAGGGAG CUGGGUAUAAUG ACAG    UAAAUCAUGU G
 U         A            C    CGAC         U
        80        70        60        50
```

sRNA (5' to 3' orientation) mapped to this predicted precursor hairpin:

in the NC library:

AAUAUGGGUCAGAGGGAGUAU (sequencing frequency: 5)

in the WC library:

AAUAUGGGUCAGAGGGAGUAU (sequencing frequency: 11)

M91（ΔG＝-38.20）

```
            10        20          30
    A                    --   U    G
UUU  CUCCCUCCGGUCCAUAAUAA  AUA GUAC \
AAA  GAGGGAGGCCAGGUAUUAUU  UAU CAUG G
    C                    CA   U    A
  70        60        50        40
```

sRNA (5' to 3' orientation) mapped to this predicted precursor hairpin:

in the NC library:

ACUUAUUAUGGACCGGAGGGA (sequencing frequency: 24)

in the WC library:

ACUUAUUAUGGACCGGAGGGA (sequencing frequency: 3)

M92（ΔG＝-55. 50）

```
          10        20        30        40         50        60
    C            U     C    G      C   -      A   ACCC   CAAU
ACUC CUCUGAUCCAUAU ACUUG CACA CUUUGU UAG AUAUGC UGU    ACA    G
UGAG GAGGCUAGGUAUA UGAAC GUGU GAAACA AUC UGUACG AUA    UGU    A
    C            -     A    G      C   A      C   GAUC   CCAA
   120        110       100       90        80        70
```

sRNA (5' to 3' orientation) mapped to this predicted precursor hairpin:

in the NC library:

UGUGACAAGUAUAUGGAUCGG (sequencing frequency: 6)

in the WC library:

UGUGACAAGUAUAUGGAUCGG (sequencing frequency: 7)

M93（ΔG＝−74. 70）

```
        10         20         30           40         50                   60         70         80
  C        U   A  G  G             A-   C--         AA---------   CCAU    G  CCG    ACGAGA   AA  G
GC AGGACCGC GAC UG UA UCGAUUUUCUCA  GAU   UUUUUGCAA           UCA    GCCC AG   CCCC      GGC  GC G
CG UCCUGGCG CUG AC AU AGCUAAAAGAGU  CUA   AAAAACGUU           AGU    CGGG UC   GGGG      CCG  CG C
  U        C   -  G  G             CC   AAA         GCCGCACCAAG   CC--    G  CUA    CAGAC-   --  C
    180      170        160       150        140        130        120         110        100        90
```

sRNA (5' to 3' orientation) mapped to this predicted precursor hairpin:

in the NC library:

AAAAUCGAGUAGCAGUCCGCG (sequencing frequency: 41)

CUGACAUGGUAGUCGAUUUUC (sequencing frequency: 3)

AUCGAGUAGCAGUCCGCGGUC (sequencing frequency: 1)

in the WC library:

AAAAUCGAGUAGCAGUCCGCG (sequencing frequency: 5)

M94（ΔG＝−47. 10）

```
          10          20          30          40
                           G    CA     C      CACC
 UAAAGCCUUAGGGAGUAUCUACA  UGG   AGUA  UUUUC      A
 AUUUCGGAAUCUCUCAUAGAUGU  ACC   UCAU  AGAAG      A
                           G    A-     A      AAAG
         80          70          60          50
```

sRNA (5' to 3' orientation) mapped to this predicted precursor hairpin:

in the NC library:

UGUAGAUACUCUCUAAGGCUU (sequencing frequency: 28)

in the WC library:

UGUAGAUACUCUCUAAGGCUU (sequencing frequency: 5)

M95（ΔG＝−99. 60）

```
         10         20         30         40         50         60         70         80         90
           G     U         CA             UGA             CCC         C    U         AA
 GCCAUAUCUU GGCUC AGGUAGGUU  AUCCUCAAUUGAA  CGAAGGAAUUAAA  AGCAGCAAA AAAC GAAAUUACC  \
 UGGUAUAGAA CCGAG UCCAUCCAA  UAGGAGUUAACUU  GCUUCCUUAAUUU  UCGUCGUUU UUUG CUUUAAUGG  A
           A     U         UA             U--             AGC         -    U         UU
```

```
   170       160       150       140           130       120        110        100
```

sRNA (5' to 3' orientation) mapped to this predicted precursor hairpin:

in the NC library:

AUCUUGGGCUCUAGGUAGGUU (sequencing frequency: 25)

in the WC library:

AUCUUGGGCUCUAGGUAGGUU (sequencing frequency: 5)

M96

super_277:408-428 **(ΔG = -60.20)**

```
        10        20        30        40
                           A  A            A
UACUCCCUCCGUUCCUAAAUUCUUGUC UG UUUUAGUUCA A
AUGAGGGAGGCAGGGAUUUAAGAACAG AC AAAAUCAAGU U
                           C  C            U
      80        70        60        50
```

sRNA (5' to 3' orientation) mapped to this predicted precursor hairpin:

in the NC library:

AAGAAUUUAGGGACGGAGGGA (sequencing frequency: 35)

in the WC library:

AAGAAUUUAGGGACGGAGGGA (sequencing frequency: 4)

super_2:20949532-20949552 **(ΔG = -39.60)**

```
        10        20        30        40
          G          C     CG GA   U   U  A
GUAUUCCCUC GUUCCUAAAU CUUGU  U  UUU AGU CA A
CAUGAGGGAG CAGGGAUUUA GAACA  G  AAA UCA GU U
          G          A     AU GC   U   U  U
       80        70        60        50
```

sRNA (5' to 3' orientation) mapped to this predicted precursor hairpin:

in the NC library:

AAGAAUUUAGGGACGGAGGGA (sequencing frequency: 35)

in the WC library:

AAGAAUUUAGGGACGGAGGGA (sequencing frequency: 4)

M97（ΔG = -64.70）

```
        10         20        30           40
              -         U  A  UC        U    U
UUGUGAAGUGUUUG GGGGAACUC UG UG  ACCAAGC  UCUA U
AGCACUUCACGAAC UCCCUUGAG AC AU  UGGUUUG  AGAU A
```

```
                 G          U  C  UA          -    G
130         120        110         100         50
                                                      60          70
                                                U C  ACAAG  AAAA  GA
                                                 G UC       CC     GA   U
                                                 C AG       GG     CU   A
                                                A C  GCGAA  GCGA  AC
                                                       90          80
```

sRNA (5' to 3' orientation) mapped to this predicted precursor hairpin:

in the NC library:

GUUCCCUGCAAGCACUUCACG (sequencing frequency: 16)

UGAAGUGUUUGGGGGAACUC (sequencing frequency: 2)

in the WC library:

GUUCCCUGCAAGCACUUCACG (sequencing frequency: 7)

M98（ΔG = -158.30）

```
      10        20        30        40        50        60        70        80         90        100        110
   CCG        U            C   CA  UC   AAA  A                  AAC     GU   -  GC     A             U       UUU
GACUG   UGUCACUUUA UCCAAAAAUGGU UGG  AG  UUA   AC CCAUGUGGCUGCCAUGUC   UUCGG  AGGA CC  GGACU CUGCUCGAUUUUC CAG GC   U
CUGAU   ACAGUGAAAU AGGUUUUUGCCA GCC  UC  AAU   UG GGUGCACCGGCGGUGCAG   AAGCC  UCCU GG  CCUGA GAUGAGCUAAAGG GUC  CG   U
   UAA        C            A   AC  CA   GUG  C                  GCU     GU   A  A-     A             -       UUU
   260       250       240       230       220       210        200       190        180        170              120
```

sRNA (5' to 3' orientation) mapped to this predicted precursor hairpin:

in the NC library:

UCACCGAACCGUUUUUGGACUA (sequencing frequency: 10)

in the WC library:

UCACCGAACCGUUUUUGGACUA (sequencing frequency: 9)

M99（ΔG = -110.10）

```
        10          20          30          40          50          60          70
    G  AA         AC    CU              -    CA   AU    CC          U    U     GUU
GGCA  AG  UGAACAUGU  AUG  GGCCAACCCAA  CCC  CG  CCU  AUCGGCAUGA  CA  GAUGU   C
UCGU  UU  ACUUGUACA  UAC  CCGGUUGGGUU  GGG  GU  GGA  UGGUCGUGCU  GU  CUAUA   U
    G  CC         GU    AC              C    AG   C-    AA          C    -     AAC
   180        170        160        150        140        130        120              90
                                                                                       100
                                                                                   A  CCAGU
                                                                                    GG      \
```

```
CC      A
A   ACUAA
110
```

sRNA (5' to 3' orientation) mapped to this predicted precursor hairpin:

in the NC library:

UGAACAUGUACAUGCUGGCCA (sequencing frequency: 5)

in the WC library:

UGAACAUGUACAUGCUGGCCA (sequencing frequency: 9)

M100（ΔG＝−47. 73）

```
         10         20            30          40         50
              C          G     --        ACUU       ACCCCCC    UUGA
UCCGAAAUAAACC GCAGUCCAC UUCU   AUUGGA     UUGCG            UCC      \
AGGCUUUAUUUGG CGUCAGGUG AAGA   UAAUUU     GACGC            AGG      U
              A          -     GC        GCAC       GCAAUUA    CUUU
        110        100           90         80         70          60
```

sRNA (5' to 3' orientation) mapped to this predicted precursor hairpin:

in the NC library:

UGGACUGCAGGUUUAUUUCGG (sequencing frequency: 4)

in the WC library:

UGGACUGCAGGUUUAUUUCGG (sequencing frequency: 23)

M101（ΔG＝-109.5）

```
       10        20        30        40         50        60        70        80        90
    U     C               C     U  C  A-      GGC     G    ACG                C    C    CUUU
UAUAA AGGGC UGCCGUUUUGCUUCU GGGAU UG GG  AAUUGU   CGGGC UGAC   GGCCAAACCCUUCCAA GAUG GUC     \
AUAUU UCCCG AUGGCAGAGCGAGGA CCCUA AC CC  UUGACA   GUCCG ACUG   CCGGUUUGGGGAGGUU CUAC CAG     C
    C     A               A     C  -  AC      GUA     A    AAA                A    A    AUGA
 180       170       160       150        140       130       120       110       100
```

sRNA (5' to 3' orientation) mapped to this predicted precursor hairpin:

in the NC library:

AGGAGCGAGACGGUAAGCCCU (sequencing frequency: 17)

in the WC library:

AGGAGCGAGACGGUAAGCCCU (sequencing frequency: 31)

M102（ΔG = --50.10）

```
              10         20         30         40
       C       G               UG  G       U  A
    GUA UCCCUCC UUCCUAAAUUCUUGU  UG UUUUAGU CA A
    CAU AGGGAGG AGGGAUUUAAGAACA  AC AAAAUCA GU U
       U       A               GU  G       U  U
           80          70         60        50
```

sRNA (5' to 3' orientation) mapped to this predicted precursor hairpin:
in the NC library:
AGAAUUUAGGGAAGGAGGGAU (sequencing frequency: 37)
in the WC library:
AGAAUUUAGGGAAGGAGGGAU (sequencing frequency: 7)

M103（ΔG = -62.60）

```
             10         20         30         40
              A           A      G             C  AG
  ACUCCCUCUG UCCAUAAUAA UGUCUU GAUUUAGUACAAA UU  \
  UGAGGGAGAC AGGUAUUAUU ACAGAA CUAAAUCAUGUUU AA  U
              C           C      G             C  CA
.         90          80         70        60       50
```

sRNA (5' to 3' orientation) mapped to this predicted precursor hairpin:
in the NC library:
UUAUUAUGGACCAGAGGGAGU (sequencing frequency: 6)
in the WC library:
UUAUUAUGGACCAGAGGGAGU (sequencing frequency: 7)

M104（ΔG = -89.70）

```
         10         20         30         40         50          60         70
    A   CAU    ACA                       AUA      -      AA  A     AA-  CG
CUUC GC   GACC   GGUUGCUACUCGAUUUUCUCAGG   UUUUU UGCAA  CC UGCCC   GC   U
GAAG CG   CUGG   CCGACGAUGAGCUAAAAGAGUCC   AAAAA ACGUU  GG ACGGG   CG   C
    C   CUC    CGC                       CCA      C      --  A     GAG  UC
      180        170        160        150        140                   80

                                                         90        100        110
                                                              CACGCCCAGAA    A  C
                                                         CGGC              GGGG UC U
                                                         GCCG              UCCC GG G
                                                               CACCAAAAG     -  G
```

```
                                 130        120
```

sRNA (5' to 3' orientation) mapped to this predicted precursor hairpin:
in the NC library:
UCGAGUAGCAGCCCGCGGUCC (sequencing frequency: 13)
in the WC library:
UCGAGUAGCAGCCCGCGGUCC (sequencing frequency: 4)

M105（ΔG = -94.50）

```
           10          20          30          40          50
                        A               UG             AU
UUGCAGGAAGAGGAGGAGAAA UGGAGGGUUCUC  GAGAAGAUGAG  \
AACGUCCUUCUCCUCUUUUUU ACUUUCCAAGAG  UUCUUCUACUC  G
                        G               GU             CA
           90          80          70          60
```

sRNA (5' to 3' orientation) mapped to this predicted precursor hairpin:
in the NC library:
GGAGGAGAAAAUGGAGGGUUC (sequencing frequency: 8)
in the WC library:
GGAGGAGAAAAUGGAGGGUUC (sequencing frequency: 5)

M106（ΔG = -85.82）

```
          10        20        30        40        50        60        70        80        90         100
     -                ACAGUU    UG   C      C   CCUU        AUAUA     AGU  G   CAA   -     AAGGAA
AUGUU UUGCUCAAGACCGCGCA     CCAUA   CA CGCAUCG UGU   GCCUAUC       CAUGA   UC AC     UG GAUCU      G
UGCAA AACGAGUUCUGGCGUGU     GGUAU   GU GCGUAGC GCA   UGGAUAG       GUACU   AG UG     AC UUAGA      A
     U                CGGACU    UG   C      U   AGUC        GAAAU     ---  G   UAC  G     AAAAGA
    190       180       170        160       150         140         130       120       110
```

sRNA (5' to 3' orientation) mapped to this predicted precursor hairpin:
in the NC library:
UUUUGCUCAAGACCGCGCAAC (sequencing frequency: 66)
UUUUGCUCAAGACCGCGCAA (sequencing frequency: 1)
in the WC library:
UUUUGCUCAAGACCGCGCAAC (sequencing frequency: 44)
UGCUCAAGACCGCGCAAC (sequencing frequency: 1)

M107（ΔG = -57.60）

```
          10        20            30             40
        G    G              G     ACU  AG      U   G
```

```
 UGAAU GCAG GCAGUUCUCCCUUG CAGG   AC  AAGCA GAU \
 ACUUA UGUC CGUCAAGAGGGGAC GUCC   UG  UUCGU CUA U
      A    A              A    CUC  G-     U   C
       90        80        70        60         50
```

sRNA (5' to 3' orientation) mapped to this predicted precursor hairpin:

in the NC library:

UGACAGGGGAGAACUGCACUG   (sequencing frequency: 6)

in the WC library:

UGACAGGGGAGAACUGCACUG   (sequencing frequency: 4)

M108（ΔG = -88.50）

```
            10          20          30          40
      -   UG        C    C      C          AC     C
 CGGCG CGG  GCCGCG UCG CGGAG AGCGUGCG  GGGC \
 GCCGC GCC  CGGUGC AGC GCCUC UCGCGCGC  CCCG A
      G   GU        A    U      C          --     A
     80          70          60          50
```

sRNA (5' to 3' orientation) mapped to this predicted precursor hairpin:

in the NC library:

CGCGCUCCUCCGUCGAACGUG   (sequencing frequency: 6)

in the WC library:

CGCGCUCCUCCGUCGAACGUG   (sequencing frequency: 6)

M109（ΔG = -60.20）

```
          10          20          30            40
    AU          C                   G     A     AAA
 UUA  CCCUCCGAU CAUAAUAAGUGUCGGG AUUU GUAC    \
 AAU  GGGAGGCUA GUAUUAUUCACAGCCC UAAA CAUG    G
    GG          A                   A     A     AUU
          80         70         70          50
```

sRNA (5' to 3' orientation) mapped to this predicted precursor hairpin:

in the NC library:

ACUUAUUAUGAAUCGGAGGGG   (sequencing frequency: 5)

in the WC library:

ACUUAUUAUGAAUCGGAGGGG   (sequencing frequency: 8)

M110（ΔG = -47.04）

```
          10          20          30             40
```

```
UC        U   A      C         CA       A   AU
  GCUGAAAA UGC AAUAGG CCAAUCUUU  AUUUU   CUC  \
  CGACUUUU ACG UUAUCC GGUUGGAAA  UAAAA   GAG  C
AA        U   -      A         AA       -   UC
   120        110       100        90   50
                                                60        70
                                        GUCUUUU   U  UCCC
                                               CGA UG     \
                                               GUU AC     C
                                        CGUUAAA   U  CCCA
                                                  80
```

sRNA (5' to 3' orientation) mapped to this predicted precursor hairpin:

in the NC library:

UGAAAAUUGCAAAUAGGCCCA (sequencing frequency: 6)

in the WC library:

UGAAAAUUGCAAAUAGGCCCA (sequencing frequency: 9)

M111（ΔG = -89.00）

```
        10        20        30        40        50
                    C                     U  C   CC    UGCC
GCCUAGUCUAUUUUCCUCUC UCCCGGUACCCUUCUUCCGGU UC AUC GGU      A
CGGGUCAGAUAAAAGGAGAG AGGGCUAUGGGAAGAAGGCCG AG UAG CCG      C
                    U                     C  A   --    CAGC
    110       100        90        80        70          60
```

sRNA (5' to 3' orientation) mapped to this predicted precursor hairpin:

in the NC library:

UGAGAGGAAAAUAGACUGGGC (sequencing frequency: 4)

in the WC library:

UGAGAGGAAAAUAGACUGGGC (sequencing frequency: 10)

M112（ΔG = -89.80）

```
        10        20        30        40        50        60          70          80
      CUC  G                     C       CUA            A   UG      AAGCC     UAU
UUGCUA   GA UUUCUCGGGGGUUUCUUGCAA AAUUUUG   AGUGUGCUAACU CU   UGAUC       CAUGC   U
AACGAU   CU AAAGAGUCCCCAAAGAACGUU UUAAAAC   UCAUACGAUUGA GA   ACUAG       GUACG   U
      AAA  G                     -       UC-            A   CA      GGUUC      UUC
        170       160         150        140         130                  90
                                                                              100       110
```

```
                                                                  AGAC    A
                                                                      UCA G
                                                                      AGU C
                                                                  AGAC    C
                                                                               120
```

sRNA (5' to 3' orientation) mapped to this predicted precursor hairpin:

in the NC library:

UUGCAAGAAACCCCUGAGAA (sequencing frequency: 12)

in the WC library:

UUGCAAGAAACCCCUGAGAA (sequencing frequency: 3)

M113（ΔG = -113.40）

```
       10        20        30        40        50        60        70        80         90       100       110
      A                       U         C          A            C    GCU      AAAAAA       AGCUAACA      A   GUC
AUUUGU CCACAGCACCAUCACAAUAGUAUA GAUGAUCCA CGGUGCGAUUG AAAAAUAUGAUUGU UGUG   CGGU        AAACAAG         CAAU UAU   C
UAAACA GGUGUUGUGGUAGUGUUAUCAUAU CUACUAGGU GCUACGCUAGC UUUUUAUACUGGCA ACGC   GCCA        UUUGUUC         GUUA GUA   A
      G                       U         U          C            U    A--      CUGCUU       CUUAAA--      A   AUC
      220       210       200       190       180       170       160         150       140       130         120
```

sRNA (5' to 3' orientation) mapped to this predicted precursor hairpin:

in the NC library:

UUGUGAUGGUGUUGUGGGACA (sequencing frequency: 4)

in the WC library:

UUGUGAUGGUGUUGUGGGACA (sequencing frequency: 10)

M114（ΔG = -85.50）

```
       10        20        30        40         50        60        70         80         90
                 C    A          CAGAUAUGUG   C   GA   AG--          -    A UG     AC        AUAGGAU
ACUACUCCCUUCAUUCC CAAA CACCUCGUA           CA UUG  UCA    ACACUCUUG UUUCU G  CCUG    CACUCAU       U
UGAUGAGGGAGGUAAGG GUUU GUGGAGUAU           GU AAC  GGU    UGUGAGAAC GAAGA C  GGGC    GUGGGUA       A
                 U    C          AUAAAAAAAGA  A   AA   GUUA          C    - GU     GG        AAUGAAA
           200       190       180       170       160       150       140                 110       100
                                                                                                   120
                                                                                                   UGA    U
                                                                                                      GCCA U
                                                                                                      CGGU A
                                                                                                   GGG    U
                                                                                                      130
```

sRNA (5' to 3' orientation) mapped to this predicted precursor hairpin:

in the NC library:

UUUGUGGAAUGGAGGGAGUAG (sequencing frequency: 9)

in the WC library:

UUUGUGGAAUGGAGGGAGUAG (sequencing frequency: 5)

M115（ΔG = -122.60）

```
         10         20         30         40          50         60           70            80         90        100        110
         AGA        UG     UG         UG          CCUG-      GC          -  -       A--      AA       A      G  C   U   U GG U     C
UGAGAUCU    GCCUU  ACAG  AAACUGAC  GUUGGGGCCA      CAGUU  CACGUGG CA AAAAAC   GACC   AGCUGA CCUGG CU GAC GGU G  G CGGG A
ACUCUAGA    CGGAG  UGUC  UUUGACUG  CAACCCUGGU      GUCAG  GUGUACC GU UUUUUG   CUGG   UCGGCU GGGCU GG CUG CCG C  U GUCU A
         CCC        CA     GU         GU          AACCA      UC          U  C       GAC      GA       A      -  U   -   - UU -     G
      220        210        200        190        180        170        160        150        140         130          120
```

sRNA (5' to 3' orientation) mapped to this predicted precursor hairpin:

in the NC library:

GUGAAACUGACUGGUUGGGGC (sequencing frequency: 4)

in the WC library:

GUGAAACUGACUGGUUGGGGC (sequencing frequency: 11)

M116（ΔG = -74.90）

```
           10          20         30         40         50
                G          A          UU       CA     -  A
UUUGUUAAGGGUAUGU AGGGUGGUA CUGAAUGUGU  GGGUG  AUCC UC C
GAACAAUUCCCAUACA UCCCACCAU GGCUUACACA  CCCAC  UGGG AG A
                A          G          CU       UG     C  C
         100         90         80         70        60
```

sRNA (5' to 3' orientation) mapped to this predicted precursor hairpin:

in the NC library:

UAUGUGAGGGUGGUAACUGAA (sequencing frequency: 285)

GUAUGUGAGGGUGGUAACUG (sequencing frequency: 2)

UGAGGGUGGUAACUGAAUGUG (sequencing frequency: 1)

AGGGUGGUAACUGAAUGUGUUUGG (sequencing frequency: 1)

UGUGAGGGUGGUAACUGAA (sequencing frequency: 1)

in the WC library:

UAUGUGAGGGUGGUAACUGAA (sequencing frequency: 103)

GGGUGGUAACUGAAUGUGU (sequencing frequency: 2)

GUAUGUGAGGGUGGUAACUGA (sequencing frequency: 1)

M117（ΔG = -67.50）

```
        10         20         30         40          50         60
```

```
        CAUCU  G  C      AA                      AU-  CA UG   UUCCC
  CCCCAG     GC GC UCUCC  GUCCGACAUUCAGGUAUACC  GC  U  CCA      C
  GGGGUU     CG CG AGAGG  CGGGCUGUAAGUCCGUAUGG  CG  A  GGU      A
        AAUCC  A  A      C-                      AUC  AG GU   UCUUC
       120       110        100        90        80        70
```

sRNA (5' to 3' orientation) mapped to this predicted precursor hairpin:

in the NC library:

UCUCCAAGUCCGACAUUCAGGU (sequencing frequency: 5)

in the WC library:

UCUCCAAGUCCGACAUUCAGGU (sequencing frequency: 14)

M118（ΔG = -56.80）

```
         10        20        30        40        50
    UGAU          CG                C   A       C  G
 AUC    GUACUCCCUC  UUCCAUAAAGAUUGG ACG AUUUGAA UA A
 UAG    UAUGAGGGAG  AAGGUAUUUCUAACC UGC UAAACUU AU G
    UAU-          AU                A   C       A  C
      100        90        80        70        60
```

sRNA (5' to 3' orientation) mapped to this predicted precursor hairpin:

in the NC library:

UUUAUGGAAUAGAGGGAGUAU (sequencing frequency: 6)

in the WC library:

UUUAUGGAAUAGAGGGAGUAU (sequencing frequency: 8)

M119（ΔG = -69.60）

```
           10        20        30        40          50        60
     C     A      CUC    G        G    --   G  U     GUGG     U
 GGUG AUUGC GGGCAA   CUCC UUGGCAGA GGAC  GAC GA GGAU     GGGG \
 CCAC UAGCG CCCGUU   GAGG AACCGUCU CCUG  CUG UU CCUA     CCCU U
     U     G      UA-    A        G    UC   G  U     AGUU     C
      130       120       110        100        90         80           70
```

sRNA (5' to 3' orientation) mapped to this predicted precursor hairpin:

in the NC library:

GGGCAACUCCUCCGUUGGCAGA (sequencing frequency: 6)

in the WC library:

GGGCAACUCCUCCGUUGGCAGA (sequencing frequency: 9)

M120（ΔG = -110.40）

```
     10     20     30     40     50       60     70     80     90       100     110
```

```
    AC         A                CUU        AC      CCAG     CA      UA        G          A     AC    UC   A   CCUUUCCCU
AGCC  UGCUGAUCG CCAGGUGGCAAGUUGA   CCACGCUG  UAAUUG     GCUG  GAAAAU  CAAAUAGG CCAAUCUUUC AUUUU   UCA   CG GGU         \
UCGG  ACGACUGGC GGUCCACCGUUCAACU   GGUGCGAC  AUUGAC     CGAC  CUUUUA  GUUUAUCC GGUUAGAAAG UAAAA   AGU   GC CCA         G
    CA         C                AAC        CU      ACAG     --      C-        G          G     CG    UU   C   CCCCUCUUA
. 230       220       210       200       190       180         170        160       150       140        130       120
```

sRNA (5' to 3' orientation) mapped to this predicted precursor hairpin:

in the NC library:

UGGCAAGUUGACUUCCACGCU (sequencing frequency: 3)

in the WC library:

UGGCAAGUUGACUUCCACGCU (sequencing frequency: 7)

M121（ΔG = -115.20）

```
       10        20        30         40        50          60        70        80        90
            -        A      A  A    UGC      CUU  GC                                  -  U       G
UAAUGUGUCGGG AAUGGUAA AUAUG AU GAU     UGCAA   GG  UGCAAUCUUGAUGUGCGGGGAUAGAUAUUC CA  UUGAGA \
AUUACACAGCCC UUGUCAUU UAUAC UA CUA     ACGUU   CC  ACGUUAGAACUGCAUGCCCUUAUCUAUAAG GU  AAUUUU G
            U        C      C  C    UCC      AGU  UA                                  A  C       A
.         170        160        150       140        130        120       110        100
```

sRNA (5' to 3' orientation) mapped to this predicted precursor hairpin:

in the NC library:

UAUCUUACUGUUUCCCGACACA (sequencing frequency: 8)

in the WC library:

UAUCUUACUGUUUCCCGACACA (sequencing frequency: 5)

M122（ΔG = -67.40）

```
        10        20        30        40         50
    CUU  GC                                  -  UC      G
GCAA   GG  UGCAAUCUUGAUGUGCGGGGAUAGAUAUUC CA  UUGAGA \
CGUU   CC  ACGUUAGAACUGCAUGCCCUUAUCUAUAAG GU  AAUUUU G
    AGU  UA                                  A  C-      A
     100        90        80        70         60
```

sRNA (5' to 3' orientation) mapped to this predicted precursor hairpin:

in the NC library:

UUCCCGUACGUCAAGAUUGCA (sequencing frequency: 56)

UAUUCCCGUACGUCAAGAUUG (sequencing frequency: 1)

in the WC library:

UUCCCGUACGUCAAGAUUGCA (sequencing frequency: 57)

UAUUCCCGUACGUCAAGAUUG (sequencing frequency: 2)

M123（ΔG＝−61.80）

```
                  10          20          30          40          50
        G      CAAG      U          C          A      A        C    U
      GAG UGCAA     GUGAC CUCAGGGUA CUUUUGACC UUUUU GUUCAAC AAA G
      CUC ACGUU     CACUG GAGUCCCAU GGAAACUGG GGAAA CAAGUUG UUU A
        G      UUAA      -          A          G      C        -    A
            110        100         90        80         70         60
```

sRNA (5' to 3' orientation) mapped to this predicted precursor hairpin:

in the NC library:

UCAAAGGAUACCCUGAGGUCA (sequencing frequency: 6)

in the WC library:

UCAAAGGAUACCCUGAGGUCA (sequencing frequency: 11)

M124（ΔG＝-60.50）

```
               10          20          30          40
                 A              C          GC   A
   UACUCCUUCCG UCCUAAAUUCUUGU GUGGUUUUA  UCA A
   AUGAGGGAGGC AGGAUUUAAGAACA CACCAAAAU  AGU U
                 C              A          AA   U
             80          70          60          50
```

sRNA (5' to 3' orientation) mapped to this predicted precursor hairpin:

in the NC library:

AAGAAUUUAGGACCGGAGGGA (sequencing frequency: 4)

in the WC library:

AAGAAUUUAGGACCGGAGGGA (sequencing frequency: 5)

M125（ΔG＝-52.90）

```
        10          20            30          40
C       U                 --            C     GC
 UCCUUUC AUCCAUAAUAAGUGUU  GGAUUUAGUACA AAUU  \
 AGGGAGG UAGGUAUUAUUCGCAG  UUUAAAUCAUGU UUAA  A
C       C                 AA            U     AC
90          80          70          60          50
```

sRNA (5' to 3' orientation) mapped to this predicted precursor hairpin:

in the NC library:

CGCUUAUUAUGGAUCGGAGGG (sequencing frequency: 6)

in the WC library:

CGCUUAUUAUGGAUCGGAGGG (sequencing frequency: 4)

M126（ΔG = -66.30）

```
         10         20         30         40           50          60
                  A  U   G      C                GA          UUG
 UAAGCCACAAAAGCACC AA UAG UGCUUU GGCUUUGGCUUUU    CAUUUGAC    A
 AUUCGGUGUUUUCGUGG UU AUC ACGAAA CUGAAACCGAAAA    GUAGAUUG    U
                  A  U   A      A                 --         UUA
       110        100        90          80         70
```

sRNA (5' to 3' orientation) mapped to this predicted precursor hairpin:

in the NC library:

UUAGGUGCUUUCGGCUUUGGC (sequencing frequency: 32)

in the WC library:

UUAGGUGCUUUCGGCUUUGGC (sequencing frequency: 6)

M127（ΔG = −63. 80）

```
          10         20         30         40
                            G          -- G   GG  AG
  CCGAGCUCCCUCAAUCUCUUUUCC CUUUCUGCU  U GCU  AA  \
  GGCUCGAGGGAGUUAGAGAAAAGG GAGAGACGA  G UGG  UU  G
                            G          UA G   GG  GA
.      90       80        70          60        50
```

sRNA (5' to 3' orientation) mapped to this predicted precursor hairpin:

in the NC library:

GGGGAAAAGAGAUUGAGGGAG (sequencing frequency: 6)

in the WC library:

GGGGAAAAGAGAUUGAGGGAG (sequencing frequency: 31)

M128（ΔG = −40. 40）

```
          10          20          30          40          50
        AU-     G    AU        U   AC          AU    AGG
 AGGGUUUU   UAGCU CCG  UCAUCCA UC  CUACCAAGA  CAUG   G
 UUUCGAAA    AUCGA GGC  GGUAGGU AG  GAUGGUUCU  GUAU   C
        AAC     G    AC        C   CC          --    AUG
     100        90         80         70          60
```

sRNA (5' to 3' orientation) mapped to this predicted precursor hairpin:

in the NC library:

ACUGGAUGGCACGGGAGCUAC (sequencing frequency: 67)

AGCUGCCGAUUCAUCCAUUCA (sequencing frequency: 4)

UGGAUGGCACGGGAGCUAC (sequencing frequency: 1)

in the WC library:

ACUGGAUGGCACGGGAGCUAC (sequencing frequency: 7)

M129（ΔG = -51.70）

```
          10         20           30         40
      U            A           CA       -      AA
  AUAC CCCUCCGUCCC AAAUAAGUGA  UGGAUU GUAUA  A
  UAUG GGGAGGCAGGG UUUAUUCACU  ACCUAA CAUAU  A
      C            C           AC       A      CU
         80         70         60        50
```

sRNA (5' to 3' orientation) mapped to this predicted precursor hairpin:

in the NC library:

ACUUAUUUCGGGACGGAGGGC (sequencing frequency: 34)

in the WC library:

ACUUAUUUCGGGACGGAGGGC (sequencing frequency: 6)

Additional data file 2. The secondary structures of predicted *Brachypodium* miRNAs. Sequences indicated in red and green color correspond to identified miRNAs and miRNA* sequences respectively.
