# Supplementary material for: Deep sequencing of Brachypodium small RNAs at the global genome level identifies microRNAs involved in cold stress response
Source: BMC Genomics. 2009 Sep 23;10:449. doi: 10.1186/1471-2164-10-449 (PMC2759970; doi:10.1186/1471-2164-10-449)
Supplement: Additional file 3 — The secondary structures of conserved Brachypodium miRNAs. is a figure showing the secondary structure of conserved Brachypodium miRNAs. [file 1471-2164-10-449-S3.pdf]

bdi-miR156a (ΔG = -67.10)

```
          10         20         30        40        50        60
-  A  -   GUCUU    -    U      -    A        A      GGUU          - A
 GG GC UGG     UGGA GGUU GACAGA AGAG GUGAGCAC CGCGGU    UCCUAGCAUG C G
 CC CG ACC     ACUU CCGA CUGUCU UCUC CACUCGUG GCGUCG    AGGGUCGUAC G G
C  -  U   -----    G    -      C    C        C      ----          C A
   120            110        100        90        80            70
```

bdi-miR156b (ΔG = -66.10)

```
        10         20          30        40        50        60
   G    G     AA-         -    -         C      GUU  G  U    CAA
GGU UCGU AGUGG   GCUGACAGA AGAG AGUGAGCAC CAUGGU   UC UU GCAU   \
UCG AGUA UCACC   CGACUGUCU UCUC UCACUCGUG GUAUCG   AG GA CGUA   G
   G    G     GAC         A    G         C      ---  G  C    CAG
 120       110       100        90        80           70
```

bdi-miR156c (ΔG = -74.60)

```
        10         20         30         40          50        60
      U    A  -   A          -    -                .-G        GC  GG
AGGGAG GAUG GA UGG GGCUGACAGA AGAG AGUGAGCACACAUGGU   CCUUUCUU  AU  \
UCUCUU CUAC UU AUU CCGACUGUCU UCUC UCACUCGUGUGUAUCG   GGAGAGAG  UA  U
      U    A  C   A          C    U                \ -        AC  AG
150       140       130       120       110                  70
                                                     80        90
                                                     GAGAAA     U
                                                           GAGCG U
                                                           UUCGU C
                                                     AAG---     A
                                                    100
```

bdi-miR156d (ΔG = -64.30)

```
        10        20          30        40        50
   C    U  GC        -     -         A      GU  UC      A  G
GGG GGCU GG  GGUGACAG AAGAG AGUGAGCAC CGGCCG  CG  ACACG CG C
CUC UCGA UC  CUACUGUC UUCUC UCACUCGUG GCCGGC  GC  UGUGC GC G
   -    -  AC        C     G         C      --  --      C  C
110         100        90        80         70          60
```

bdi-miR156e (ΔG = -64.70)

```
        10        20          30        40          50
      GCCUC     AU        -   -         A     -   UGUC      A
GAGAGG     GAGAG UGACAGAA GAG AGUGAGCAC CGGCG UGA    CGGCAU U
CUCUCU     UUCUC ACUGUCUU CUC UCACUCGUG GCCGC GCU    GCCGUA G
      -----     CG        U   C         C     U   ----      U
110            100        90        80        70            60
```

bdi-miR156f (ΔG = -96.60)

```
        10         20        30          40        50        60          70          80          90
    AG     -  A  GCUAU          -     -        A       C  ACU     U    C--    GCG--  GGA--  C      GC
UGGC  GUGUG GC AG     GGGUGACAGA AGAGA GUGAGCAC CAGCGGC AG   GCAUC AUUC   UUCG     GC     CA GGCCGG  A
AUCG  CACAC CG UC     UCCACUGUCU UCUCU CACUCGUG GUCGUCG UC   CGUAG UAAG   AAGC     CG     GU CCGGCC  U
    G-     A  C  -----          C     U        C       -  GU-     -    CAA    ACAUA  GUACG  A      GG
     180            170       160       150       140         130        120       110       100
```

bdi-miR156g (ΔG = -74.60)

```
        10        20          30        40        50        60
    AUG  U   CGC         -    -         A      GGCGG         CGGC
GGUC   UG GCG   GGUGACAGA AGAG AGUGAGCAC CGGCCG     GACGGCACC    \
CCGG   AC CGU   CCACUGUCU UCUC UCACUCGUG GCCGGC     CUGCCGUGG    G
    AAG  C   UU-         G    G         C      G----         UUAG
  120       110        100        90        80            70
```

bdi-miR160a (ΔG = -57.10)

```
                  10        20        30         40         50
    C -  ---------       C       C          A   -   GC      -  U
UCAU G GC         UGCGUGC UGGCUCC UGUAUGCCAC CAU GUA  CCGAUC CG \
AGUA C CG         ACGUACG ACCGAGG ACGUGCGGUG GUG CGU  GGUUGG GC G
    C U  AACAUACAU       A       A          G   U   A-      A  C
    110       100        90        80        70         60
```

bdi-miR160b (ΔG = -67.20)

```
        10        20        30         40        50        60
AUG  A     C       C         CU    -    CU      AACUCA     A  UCC
   GG UAUGC UGGCUCC UGUAUGCCA  CGC GUAG  GCCAAC      GUUUG GC   \
   CC GUACG ACCGAGG GCGUGCGGU  GCG CAUC  CGGUUG      CAAAC CG   C
UG-  A     A       A         AG    C    U-      CAAACG     C  GCC
     120       110       100        90         80        70
```

bdi-miR160c (ΔG = -62.30)

```
            10        20        30        40        50
           C       C         CU      AUA      AU--   CU
  GGGAUGUGC UGGCUCC UGUAUGCCA  CAUCU   GCAAC    UC  U
  CCCUAUACG ACCGAGG ACGUGCGGU  GUAGG   CGUUG    AG  C
           A       C         AG      AUC      GAGU   UC
     100        90        80        70        60
```

bdi-miR160d (ΔG = -56.80)

```
        10             20        30        40         50
     A  CU----    U    C       C          A   -    CC   C    G
UGAUCA GA      GUGC GUGC UGGCUCC UGUAUGCCAC CAU GUAG  CAG CCAU A
ACUAGU UU      UACG UACG ACCGAGG ACGUGCGGUG GUG CGUC  GUU GGUG A
     A  ACGCAU    -    A       A          G   U    C-   U    U
 .       110        100        90        80        70         60
```

bdi-miR162 (ΔG = -54.20)

```
        10        20        30        40          50
GC    GC    U            CU     U  CU  U   .-ACUGAU    G
  CUGG  GCAG GGUUUAUCGAUC  UUCCC GC  UG GCU         CCUG \
  GGCC  CGUC CCAAAUAGCUAG  AAGGG CG  AU CGA         GGGC G
UU    UA    U            CU     -  U-  U   \ ------    C
 130       120       110        100                60
```

bdi-miR164a (ΔG = -78.20)

```
                10        20        30        40          50
      ------      CGU     A  C                U  .-A|     AGC
AGCGAGA      AGGACCG   UGGAG AG AGGGCACGUGCAUGCA GC   ACUAG    \
UCGCUCU      UUCUGGC   ACCUC UC UCUUGUGUACGUACGU CG   UGAUC    U
      CUUUAC      ACU     C  U                -  \ -^     GAU
    180       170       160       150       140         60
                                                          70        80             90        100
                                                      GUCAAUUCU    UCUCUU    U-----    -    GAUCGAU
                                                               UCCA     GUGCC     GGC CGG        \
                                                               AGGU     CACGG     UCG GCC        C
                                                      U--------    UAUC--    UUUAAU    A    AUAUAUU
                                                                 130         120       110
```

bdi-miR164b (ΔG = -65.70)

```
        10        20        30        40          50        60
         U   CC  U        CA    A      AA--|  GGA      UU     GU
UGGUGAGAA GCC  UG UGGAGAAG  GGGC CGUGCA    GAUC   GCACGU  CGUAU  \
ACCAUUCUU CGG  AC ACCUCUUC  CUCG GCACGU    CUAG   CGUGUA  GCAUG  G
         C   U-  C        CC    C      GUCG^  GUA      U-     UA
    120       110       100        90        80         70
```

bdi-miR164c (ΔG = -109.20)

```
        10        20        30        40        50           60        70        80
         GC   A          C             UCGUUCCA  U   -|  -  -   U    C     GGCC      - G
AGGCGAGGC  GCG GGUGGAGAAG AGGGCACGUGCAU        GC CGC CGC CG GUG GCCG GCCGC    UGGGCG C C
UCCGCUCCG  CGC CCACCUCUUC UCCCGUGUACGUA        CG GUG GUG GC UAC CGGC CGGCG    GCCCGC G G
         AA   G          U             --------  U   U^  C  G   -    -     A---      U U
 .       150       140       130              120       110         100           90
```

bdi-miR164d (ΔG = -101.30)

```
           10        20        30        40         50              60           70
            U    C          A       A     GCACA- CA-  -    AUG---    -     --  -     ACA
   AGGCGAGGC UGCG GGUGGAGAAG AGGGCAC UGCAU      CA   CG CGC      GCCG GCCGC  UC GGGCG   \
   UCCGCUCCG GCGC CCACCUCUUC UCCCGUG ACGUA      GU   GC GCG      CGGC CGGCG  GG CCCGC   C
            C    U          G       C     AGCAAG CGA  G    GCCACA    G     CC  A     GCG
         160       150       140       130       120       110       100         90        80
```

bdi-miR166a (ΔG = -104.20)

```
      10        20        30        40        50           60        70        80        90        100       110
          UUCGU     A         UU       CU        CUCUU---   U     UGC       C        CCUCCUCAA    UCU       GA          GA   AGA
GGGAGCCUUU     UUUG GGGGAAUG  GUCUGG  CGGGGUU        UAU UCUA   CUCUCU UAUCCCCC         UCUC   AGCUUU  UUGGAUCGA  GA    \
CCCUCGGGAA     AAAC CCCCUUAC  CGGACC  GCUCUAG        AUG AGAU   GAGAGA GUAGGGGG         AGAG   UUGAGA  AACCUAGCU  CU    U
          CAU--     C         UU       AG        AUGUGUGU   C     ---       A        A--------    UC-       AG          AG   AGC
220       210         200       190       180       170          160       150                 140       130       120
```

bdi-miR166b (ΔG = -68.60)

```
          10        20        30        40        50
U        --   A          C   G          AAGA      A   CGGGA
 UCAUGGUU  GUCG GGGGAAUGA GCC GGUCUGAAAGA   AGGGGGC CGC     \
 GGUACCAG  CAGU CCCCUUACU CGG CCAGGCUUUCU   UCCCUUG GCG     A
G        CU   A          U   A          CGA-      -   ACAGU
       110       100        90        80         70
```

bdi-miR166c (ΔG = -56.10)

```
        10        20          30          40         50
A        UU        G      UA--  -  -     UAGCA       -   C
 GGGGAAUG  GUCUGGUU GAGACC    GC UA GCAGC     CGGAUC AUC A
 CCCCUUAC  CGGACCAG CUUUGG    CG AU CGUCG     GCUUAG UAG U
A        UU        G      CGCG  U  A     CA---       C   A
      100        90        80        70          60
```

bdi-miR166d (ΔG = -66.60)

```
        10        20        30        40            50        60
        UUU     U          UU      CU      G---  -          AGAAC
GGGAGCUU   UACUU GAGGGGAAUG  GUCUGG  CGAGGU    CU GAGAUCGAG     \
CCCUCGAA   AUGAA CUCCCCUUAC  CGGACC  GCUCUA    GA CUCUAGCUU     U
        ---     -          UU      AG      GUUA  U          AAAGU
 .          110        100        90        80        70
```

bdi-miR166e (ΔG = -71.10)

```
        10        20        30        40        50        60         70
          UUGCU      U      UU         A         CAUGU  UU     -    C    U   C
UGAAGCUAUU     UCUGGG GGAAUG  GUCUGGUUC AGGUCUUGC     GAU  GAGGA UGAA GGUU GUG A
ACUUCGAUAG     AGACUC CCUUAC  CGGACCAGG UCUAGGGCG     UUG  CUCUU ACUU UCAA UAC U
          -----      C      UU         C         U----  UU     U    -    -   G
      140            130        120        110           100        90          80
```

bdi-miR166f (ΔG = -67.60)

```
         10        20        30        40           50         60
   U--   C      A    C   UC  G   C        G      G---  -     A    U    U
UCC   CGU UUGUUG GGGG AUG  GU UGG CCGAGACC GUCAU    GC CGUCG UCGG CGGC A
AGG   GCA GGCAAC CCCC UAC  CG ACC GGCUCUGG UAGGUG   CG GCGGC GGCC GCUG A
   CGU   C      C    U   UU  G   A        -      ACUG  C     -    -    U
     130        120        110        100         90        80          70
```

bdi-miR166g (ΔG = -64.30)

```
        10        20        30        40        50
     A      CU         G   A   A            CCG      UGUG
GGAAGC UGGUGU  GGAAUGGAG CUG UCC AGAUCCUUGGU   AAAUAU    \
CCUUCG GCCACA  CCUUACUUC GAC AGG UCUAGGAACCG   UUUGUG    U
     A      CU         G   C   C            UUG      UCUU
   110       100        90        80        70        60
```

bdi-miR167a (ΔG = -61.40)

```
        10          20          30         40
     C         C--            -U     C-    G     A
GAAAG GUGAAGCUG   CAGCAUGAUCUA   CUGA  UUGU GUGGC A
CUUUC CACUUUGAC   GUCGUACUAGAU   GACU  AACG CACCG G
     U         AGU             -     UA    A     U
    120       110       100        60         50
                                     70
                                CA-    C  -    U
                                   ACGC GU GGCU C
                                   UGUG CG CCGG G
                                UGG    A  G    G
                                     90        80
```

bdi-miR167b (ΔG = -90.70)

```
             10        20          30        40        50        60
       -----     C      A      --              UCA        AAG              AU
UGCCCGA     GGGAA GAGUGA GCUGCC  AGCAUGAUCUAGC   GCGUGAUC   CAAGAUU--CACAC  A
ACGGGUU     CCCUU UUCACU CGACGG  UCGUACUGGAUCG   CGUACUGG   GUUCUAG  GUGUG  C
       UGCCG     C      C      CG              UUC        A--       \      CA
   160       150       140       130       120       110                  70
                                                                       80         90
                                                                     GUUUUU       U
                                                                           UUGAGC A
                                                                           AGCUCG U
                                                                     UU----       A
                                                                      100
```

bdi-miR167c (ΔG = -58.30)

```
              10        20          30        40          50
       ------     AG     A    C  -            UUU        -     UG
AUGCUCA      UGUGAA  AGUGA GCUG CA GCAUGAUCUAGC   GAUCCA AUGUA  U
UACGGGU      ACGCUU  UUACU CGAC GU CGUACUAGAUUG   CUAGGU UGCAU  C
       UUGCAA     CU     C    A  C            UUC        G     CU
120       110       100        90        80        70        60
```

bdi-miR168 (ΔG = -62.80)

```
          10        20        30        40         50
   --     C   G GC              AU     CCC    -     CCC
CGC  CGCCG CUC G  UCGCUUGGUGCAG  CGGGA   UCCG CCCG   \
GCG  GCGGC GAG C  AGUGAACCACGUU  GCCCU   AGGC GGGC   C
   AC     C   G UA              CC     ---    C     CGC
          90        80        70           60
```

bdi-miR169a (ΔG = -71.40)

```
        10          20        30        40          50          60
  U      UG A   -          UG          U-    --     UA--  CU       AUUA
GA AGAGAG  G AUG UAGCCAAGGA  ACUUGCCGGG  AUGU  GCAUG    CG  UCAGGGA    U
CU UCUCUC  U UAC AUCGGUUCUU  UGGACGGCCC  UAUA  CGUAC    GU  GGUCCCU    A
  U      GU C   A          GU          UU    GU     CUAA  CU       ACGU
      130       120       110       100        90        80        70
```

bdi-miR169b (ΔG = -48.00)

```
        10        20           30         40         50
GACA  UCU              --- UG          -   C  CAUAUA-   CUCC
    GC   UCGUGUAGCCAAGG   A  ACUUGCCGGCC GGC UG        UGU    A
    CG   AGUACAUCGGUUUC   U  UGAACGGCUGG UCG AC        ACA    A
CUC-  UC-              GUU GU          A   A  AAUUCGA   AUUA
       110       100        90        80        70        60
```

bdi-miR169c (ΔG = -61.90)

```
        10          20        30        40        50          60
       G  UAU   UGA-          U    U       UCA-   UU   -      -   AC
GCAAGGG CU   CUC    UAGCCAAGGA GACU GCCUGUG    UAC  CAA GGCUUG GCA  A
CGUUCUC GG   GAG    AUCGGUUCCU CUGA CGGAUAC    AUG  GUU CCGAGU CGU  U
       -  U--   UCCG          -    -       UCUG   UU   U      A   AG
        120         110        100         90        80        70
```

bdi-miR169d (ΔG = -71.10)

```
        10        20          30        40       50        60        70
          UUG    UGA-          U    U       C   UCC  CCUU      U      AUU
GAUAAGGGGU   GCCC    UAGCCAAGGA GACU GCCUGUG AUG   UC    GAUCGC UGCAUA    U
CUAUUCUCCG   UGGG    AUCGGUUCCU CUGA CGGACAC UAU   AG    CUAGUG ACGUAU    C
          ---    UCCG          -    -       -   UU-  CU--      U      AUA
      130          120        110        100            90        80
```

bdi-miR169e (ΔG = -69.10)

```
             10        20         30         40          50
   C         A       C      C--     U  GU          CU      AAU
     AGCCAAGA UGGCUUG CUAUGC   CACGU CU  UUCAUCACCA  GGGCUU   \
     UCGGUUCU ACCGAAC GGUAUG   GUGUA GA  AGGUAGUGGU  CCCGAG   C
    A        -       U      UUA     -  GU          --      GUU
     110        100       90        80        70          60
```

bdi-miR169f (ΔG = -64.10)

```
         10         20         30        40                    50
     C      G-   A  C        UG          ----  CU   --------    G GG
AGAGG AGAGAA  GGG UG AGCCAAGGA  ACUUGCCGGC    UC  GGU         GUUG G  \
UCUUC UCUCUU  UCC AC UCGGUUCCU  UGGACGGCCG    AG  CCG         CGAC U  A
     U      GA   -  A        GU          AUUG  UU   AGUUGUUU    G UA
    120        110        100        90        80        70        60
```

bdi-miR171a (ΔG = -49.30)

```
         10          20         30        40         50
AA         C--         GA         C     U         -  U
  UGGAAGGAG   GAUAUUGGU  GGUUCAAUC GAUGC AGAUUUUA CA C
  AUCUUCUUC   CUAUAACCG  CCGAGUUAG CUAUG UCUAGAAU GU A
--         UCU         UG         U     -         U  G
  100        90         80         70         60
```

bdi-miR171b (ΔG = -73.70)

```
          10         20         30         40         50         60
   U-     AG                       A  C      UCUCUG   -   CUG     C
GGC  GGGAG  UGCGAUGUUGGCACGGUUCAAUCA AU GGGUGG      CAU GCA   GCUUG G
CCG  UCUUC  ACGCUAUAACCGUGCCGAGUUAGU UA CUCGCU      GUA CGU   CGAAC A
   UU     GA                       C  C      UUGUA-   G   ---     G
130       120        110        100        90         80           70
```

bdi-miR171c (ΔG = -62.20)

```
          10         20         30         40         50          60
   -      U   C    U         U  A    C     C      CG    --    GAU    U
AUG AUGAAA GGC ACUA GAUGUUGGC CG CUCA UCAGA GACAC  GCCG  GAGC   GCG G
UAC UACUUU UCG UGAU CUAUAACCG GC GAGU AGUCU UUGUG  CGGC  CUCG   CGU C
   G      C   -    U         U  C    U     -      --    AG    AU-    G
.        120        110        100        90           80         70
```

bdi-miR171d (ΔG = -53.40)

```
        10        20        30        40        50        60
     AUUAAUGUAUUAU     C        UG             A  CU  C     GGAC
UCAAG             AGACG GGUAUUGG  CGGUUCAAUCAGA AG  GG GCUCC    G
AGUUC             UUUGC CUAUAACC  GCCGAGUUAGUUU UC  CC UGGGG    C
     GU-----------     A        GU             C  U-  U     AAAA
                 110       100        90        80         70
```

bdi-miR172a (ΔG = -60.90)

```
         10        20        30        40           50        60
     -   C    A         C  C        A    CG    ---    AG    AAUU
AGUCG GCG UUGC GGUGCAGCA CA CAAGAUUC CAUC  GGUU   CUCC  UCGU    \
UCAGC CGC AACG CUACGUCGU GU GUUCUAAG GUAG  CCAA   GAGG  AGCA    A
     U   C    G         A  A        A    UA    CAU    --    AUUA
 120       110       100        90        80        70
```

bdi-miR172b (ΔG = -106.30)

```
        10        20        30        40          50        60        70           80         90        100       110
     G    U    C  UG               A    AAU--  A  C     UC     U  CA         ---     U       -     U     CUA    AAG     AU
AGUCG UGAU UCUG UG   GCAUCAUCAAGAUUC CACA     GC UG UAGCU  GUCUA GC  UCCUUUCU   CAUCU UCGAUCU CGAUC CGGCC   AUU    UAAUU  A
UCAGU ACUA AGGC AC   CGUAGUAGUUCUAAG GUGU     CG AU AUCGA  UAGAU CG  AGGAGAGG   GUAGG AGCUAGA GUUAG GCCGG   UAG    GUUAA  C
     G    C    U  GU               G    GUAUU  -  -     U-     -  --         UGC     U       G     -     C--    GU-     CC
    220       210       200       190       180         170           160       150       140        130          120
```

bdi-miR319 (ΔG = -84.90)

```
           10        20        30        40        50         60        70        80        90
A--    --    A    U            CU U    UG  UGU        AU-     G    AU       U  AC         AU    AGG
   UGUA  UAAG GAGC CUCUUCAGUCCA  C CAGA  AC   AGGGUUUU   UAGCU CCG  UCAUCCA UC  CUACCAAGA  CAUG    G
   ACAU  GUUC CUCG GGGAAGUCAGGU  G GUUU  UG   UUUCGAAA   AUCGA GGC  GGUAGGU AG  GAUGGUUCU  GUAU    C
AUA    UC    C    U            UG U    GU  CU-        AAC     G    AC       C  CC         --    AUG
.       180       170       160       150        140       130       120       110         100
```

bdi-miR390 (ΔG = -78.00)

```
           10         20         30           40         50         60             70         80
     ---     AAU  U  A          G          C       C  U C     A      .-ACCGGCCAUC          C
GGUAAG   GAAC    CC UG AGCUCAGGA GGAUAGCGC  UAGGUU AC G AACCA CAAGAGA             GAUAUAUA A
CCAUUC   CUUG    GG AC UCGAGUCCU UCUAUCGCG  GUCUAG UG C UUGGU GUUCUCU             CUGUAUAU U
     UUG     CUU  C  C          A          -       C  - C     C      \ ----------          A
   180        170        160        150           130        120                         90
                                                                               100
                                                                            C      G
                                                                             UCGAUC A
                                                                             AGCUAG C
                                                                            -      C
```

bdi-miR393 (ΔG = -62.80)

```
        10        20        30        40            50          60
    U  U        A                U      U---     UC-  U  -      UU
AAUG CU GGGGAAGC UCCAAAGGGAUCGCA UGAUCC    UCGAC   CA UA AUGGUG  \
UUAC GA CUCCUUCG AGGUUUUCCUAGCGU ACUAGG    AGCUG   GU GU UGCUAC  C
    C  U        G                -      CAGC     UUA  C  C      UG
   120       110       100         90        80        70
```

bdi-miR394 (ΔG = -99.10)

```
        10        20        30        40        50        60                70          80
             GC        C      CGAG      -      UUC           -------       U    -   G     U
GUCAUGUGGGUUU  CAAAGGGG GCUUAC    AGCUCU UUGGCA   UGUCCACCUCC       UUGCGAG CUCG CGG AGAUC C
CGGUAUACCCAAA  GUUUCCCU CGGAUG    UCGAGG AACCGU   ACGGGUGGAGG       GACGUUC GAGU GUU UCUAG A
             AU        U      UG--      U      CAU           UUCGAUA       -    U   G     U
170       160       150         140       130       120       110       100         90
```

bdi-miR395a (ΔG = -44.60)

```
        10        20        30        40
    AU     U  A      A             GA    AUU
UGGU  UAUCA GA UUCCCU CAAGCACUUCACAA  AGCU   C
ACCA  GUAGU CU AAGGGG GUUUGUGAAGUGUU  UCGG   C
    CU     U  C      -             A-    GAU
         80        70         60         50
```

bdi-miR395b (ΔG = -48.60)

```
        10        20        30         40
U   AU     U          G           A-    CAAU
 GGU  UACCA GAGUUCCCU CAAGCACUUCACG  GGCC    U
 CCA  GUGGU CUCAAGGGG GUUUGUGAAGUGU  UCGG    A
-   CU     U          -           CA    UAGU
         80        70         60        50
```

bdi-miR395c (ΔG = -42.70)

```
        10        20        30        40        50
U   AU     U         UG           UACGA   A  AU
 GGU  UACCA GAGUUCCU   CAAGCAUUUCACG     GGC CU  C
 CCA  GUGGU CUCAAGGGG  GUUUGUGAAGUGU     UCG GA  C
A   CU     U         --           UA---   G  AC
```

```
   90         80           70         60
```

bdi-miR395d (ΔG = -69.60)

```
        10         20         30           40
      A          U        U       .-GU|     C
UUGUCA CUGGAGUUC CCUCAAA CACUUCA    AGCUAG U
AACGGU GGCCUCAAG GGGGUUU GUGAAGU    UCGAUC A
      G          -        -       \ --^     G
 140        130          120         50
                                     60         70
                                  -     ------   AUU       U  -    GUU
                                   UGUGC      CUC   GUUUCAU GC CGCU   \
                                   ACACG      GAG   CGAGGUA CG GUGA   U
                                  G     UGCCGA   GU-       -  U    AUA
                                   110        100          90          80
```

bdi-miR395e (ΔG = -78.50)

```
        10         20         30           40          50         60         70         80
             UG      U       U       --  A  -      AG U  U   U  UUGUU   UUGC    GUU
GUUGGUUGUCACC  GAGUUC CCUCAAA CACUUCA  GU GC UAGCU  C UG GCC CC     GCA    CACU   U
CGACCAACGGUGG  CUCAAG GGGGUUU GUGAAGU  CA CG GUCGA  G AC CGG GG     CGU    GUGA   U
             GU      -       -       GA  -  U      GA -  -   -  UU---   ----    AUA
.       140        130          120         110         100               90
```

bdi-miR395f (ΔG = -56.50)

```
        10         20         30         40
        UCC             U               GCA    U
GUUGGUG   CCGGGAGUUCCCU CAAACACUUUACGAA   UCUU \
CGAACCAC   GGCUCUCAAGGGG GUUUGUGAAGUGUUU   AGAA C
        UGU             -               AUC    U
     90         80          70         60         50
```

bdi-miR395g (ΔG = -50.00)

```
        10         20         30         40
    U  C            G              GU      U
GGUG UA CAGGAGUUUCCU CAAACACUUCACGA  GCCUAU U
CCAC GU GUUCUCAAGGGG GUUUGUGAAGUGUU  UGGGUA U
    U  A            -              GU      A
```

```
        80        70         60        50
```

bdi-miR395h (ΔG = -46.50)

```
        10        20         30
    U         G             A-    G  U
ACCA GAGUUCCCU CAAGCACUUCACG  GGCC UU U
UGGU CUCAAGGGG GUUUGUGAAGUGU  UCGG AG C
    U         -             CA    G  U
     70         60        50         40
```

bdi-miR395i (ΔG = -51.10)

```
        10        20        30         40
         U         G            GA-     AAU
GUAAUACCA GAGUUCCCU CAAGCACUUCAU   GGCCU   U
CAUUGUGGU CUCAAGGGG GUUUGUGAAGUG   UCGGG   C
         U         -            UCA     AAU
     80        70         60        50
```

bdi-miR395j (ΔG = -44.90)

```
        10        20        30         40
   AUUC   U         G             A-    G  U
GGU    UCA GAGUUUCCC CAAGCACUUCACG  GGCC UU U
CCA    GGU CUCAAGGGG GUUUGUGAAGUGU  UCGG AG C
   CUCU   U         -             CA    G  U
        80        70          60          50
```

bdi-miR395k (ΔG = -49.30)

```
        10        20        30         40
   C     U         G            GA-     AAU
GUA UACCA GAGUUCCCU CAAGCACUUCAU   GGCCC   U
CAU GUGGU CUCAAGGGG GUUUGUGAAGUG   UCGGG   C
   U     U         -            UCA     AGU
     80        70         60        50
```

bdi-miR395l (ΔG = -51.30)

```
        10        20        30         40
       AU     U         G            A-   A  AU
GUUUGGU  UAUCA GAGUUCCCU CAAGCACUUCACG  GGC CU  C
CGAACCA  GUGGU CUCAAGGGG GUUUGUGAAGUGU  UCG GA  C
       CU     U         -            CA   G  AC
     90        80         70        60        50
```

bdi-miR395m (ΔG = -42.70)

```
        10         20         30         40
      AAU     U          G    A         A  UC  AUU
GUUUGG   UAUCA GAGUUCCUU CAAA ACUUCACG GG  CA   C
CGAACC   AUAGU CUCAAGGGG GUUU UGAAGUGC CC  GU   U
      ACU     U          -    G         -  UC  GAA
     90         80          70         60          50
```

bdi-miR395n (ΔG = -48.00)

```
        10         20         30          40
U   AU     U          G            C  -  A  AC
 GGU  UACCA GAGUUCCCU CAAGCACUUCAC AU GC CU  C
 CCA  GUGGU CUCAAGGGG GUUUGUGAAGUG UA CG GA  C
A   CU     U          -            U  U  G  AC
         80         70          60         50
```

bdi-miR396a (ΔG = -75.50)

```
        10         20         30         40         50          60
      C  G      C      CA                 G   UC       .-U    UC
GAGAUG GC GGCAUG UUUCCA  GGCUUUCUUGAACUGU AAC  GUGGGGG   GUUC  C
UUCUAC CG CCGUAC AAAGGU  CCGAAAGAACUUGGUA UUG  CGCCUCU   UAAG  U
      -  -      C      AC                 G   UC       \ -    UC
 .         150        140        130        120        110
                                                                            70          80         90
                                                                             UGU    -    AUUGC   C
                                                                                GUCG UGGA      GGG U
                                                                                UAGC GCCU      CUU U
                                                                             C--    U    AUUU-   G
                                                                                        100
```

bdi-miR396b (ΔG = -73.50)

```
        10         20         30         40           50
    U          CUC    CA                 G   .-UCGU   C    C
AGGU GCGGCCAUG   UCCA  GGCUUUCUUGAACUGU AAC      GCG CUAG C
UCCG CGCCGGUAC   AGGU  CUGAAAGAACUUGGUA UUG      CGU GAUC U
    C          AAA    UC                 G   \ ----   C    G
    170        160        150        140                 60
                                                  70          80
```

```
.-U      -    U-   AUCCU
   CAAUUC GAUC  GUC     \
   GUUAAG CUAG  UAG     C
\ -      U    CU   CUUGG
          100        90
```

bdi-miR397a (ΔG = -76.00)

```
          10        20        30        40         50
     C--        U      A      C     U       U     C-   -  C
AGAGG   GCAGAGGC UGAUUG GUGCAG GUUGA GAACCCC CUGCG  CCA GG C
UCUCC   CGUCUCCG ACUAAC CACGUC CAGCU CUUGGGG GGCGC  GGU CC G
     UUU        C      C      C     U       -     AA   A  G
   110       100        90        80         70        60
```

bdi-miR397b (ΔG = -63.90)

```
         10        20        30        40        50
     C-    A   A      A           A     AG     AGGC      C
AGAGG  GCAA GGC UCAUUG GUGCAGCGUUG UGAAC  GGGCC    GACCGG \
UCUCU  CGUU CCG AGUGAC CACGUCGCGGC ACUUG  CUUGG    CUGGCC G
     UA    C   C      A           C     G-     C---      G
110       100        90        80         70           60
```

bdi-miR398 (ΔG = -67.90)

```
         10        20        30        40          50
   CA   -   A     UU  U      U   A             .-AGAU  AU
CAG  CAU GCG GGAAG  CC GCAGGG CGA UUGGGAACACAUG      GG  \
GUU  GUA CGU CCUUC  GG UGUCCC GCU GACUCUUGUGUAC      CC  G
   AG   C   G     UG  U      C   G             \ ----  UG
         120       110       100        90
                                                60        70
                                                          UGA
                                                 GCUGAGCUC   \
                                                 CGGCUCGAG   G
                                                          CUC
                                                    80
```

bdi-miR528 (ΔG = -74.30)

```
         10        20        30        40        50        60
   U  -        CG            U   G     CAGG     U      UUGUACU
GGC GG AGCAGCAG  GUGGAAGGGGCA GCA AGGAG    GAUGA GGGGGG       \
CCG CC UCGUCGUC  UACCUUCUCCGU CGU UCCUC    UUGCU UCCCCU       C
   -  A        CU            C   G     ----     C      UCUUCGU
        110       100        90            80        70
```

bdi-miR529 (ΔG = -52.70)

```
          10        20        30        40         50
       --     AG             A          A  -    CUC  U
CGUCAAG  GUUAA  GGAGAAGAGAGAG GUACAGCCUU AU UAAG   GA C
GUAGUUC  CGAUU  CUUCUUCUCUCUC CAUGUCGGAA UA GUUC   CU G
       UU     CU             C          C  U    UGU  A
     100        90        80        70        60
```

bdi-miR827 (ΔG = -60.80)

```
           10         20        30        40        50        60         70
   -   G--   -     U  U         U               A     CC   CUUC     UU-  GC
CAU GCA   CUC UGAAC UG UUUGUUGGU GUCAUCUAACCAUCG UCGGU  GUG    GGUGC   GU  C
GUG CGU   GAG GCUUG AC AAACGACUA CAGUAGAUUGGUAGC GGCCA  CAC    CUACG   UA  A
   C   ACA   U     U  -         C               -     --   UA--     UUU  GU
 .       130       120        110       100         90            80
```

Additional data file 3. The secondary structure of conserved *Brachypodium* miRNAs. Sequences indicated in red and green color correspond to identified miRNAs and miRNA* sequences respectively.
