# Supplementary material for: Deep sequencing of Brachypodium small RNAs at the global genome level identifies microRNAs involved in cold stress response
Source: BMC Genomics. 2009 Sep 23;10:449. doi: 10.1186/1471-2164-10-449 (PMC2759970; doi:10.1186/1471-2164-10-449)
Supplement: Additional file 4 — Multiple sequence alignment of miR395 genes in Brachypodium and their homologs in rice. is a figure showing multiple sequence alignment of Brachypodium miR395 genes as well as their homologs in rice, performed with the ClustalW 1.83 program. [file 1471-2164-10-449-S4.doc]

**(a)**

bdi-miR395c ----------------------------------------------------TGGTATTA

bdi-miR395n ----------------------------------------------------TGGTATTA

bdi-miR395a ----------------------------------------------------TGGTATTA

bdi-miR395l -------------------------------------------------GTTTGGTATTA

bdi-miR395i ------------------------------------------------------GTAATA

bdi-miR395k ------------------------------------------------------GTACTA

bdi-miR395b ----------------------------------------------------TGGTATTA

bdi-miR395h -----------------------------------------------------------A

bdi-miR395j -----------------------------------------------------GGTATTC

bdi-miR395m -------------------------------------------------GTTTGGAATTA

bdi-miR395g -----------------------------------------------------GGTGTTA

bdi-miR395f -------------------------------------------------GTTTGGTGTCC

bdi-miR395d -----TTGTCAACTGGAGTTCTCCTCAAATCACTTCAGTAGCTAGCTAGCTAGCTTGTGC

bdi-miR395e GTTGGTTGTCACCTGGAGTTCTCCTCAAATCACTTCAGTAGCTAGCTA----GCTTGTGC

bdi-miR395c CCATGAGTTCCTTTGCAAGCATTTCACGTACGAGGCACTATCCCAA-GGGCT----ATTG

bdi-miR395n CCATGAGTTCCCT-GCAAGCACTTCACC----ATGCACTACCCCAA-GGGCT----ATTG

bdi-miR395a TCATGAATTCCCT-ACAAGCACTTCACA----AGAAGCTATTCCTA-GGGCT----ATTG

bdi-miR395l TCATGAGTTCCCT-GCAAGCACTTCACG----AGGCACTATCCCAA-GGGCT----ACTG

bdi-miR395i CCATGAGTTCCCT-GCAAGCACTTCATG----AGGCCTAATTCTAA-GGGCT----ACTG

bdi-miR395k CCATGAGTTCCCT-GCAAGCACTTCATG----AGGCCCAATTCTGA-GGGCT----ACTG

bdi-miR395b CCATGAGTTCCCT-GCAAGCACTTCACG----AGGCCCAATTATGA-TGGCT----ACTG

bdi-miR395h CCATGAGTTCCCT-GCAAGCACTTCACG----AGGCCGTTTTCTGA-GGGCT----ACTG

bdi-miR395j TCATGAGTTTCCC-GCAAGCACTTCACG----AGGCCGTTTTCTGA-GGGCT----ACTG

bdi-miR395m TCATGAGTTCCTT-GCAAAAACTTCACG----AGGTCCAATTCTAA-GTGCT----CCCG

bdi-miR395g CCAGGAGTTTCCT-GCAAACACTTCACG----AGTGCCTATTTTAATGGGTT----GTTG

bdi-miR395f CCGGGAGTTCCCT-TCAAACACTTTACG----AAGCATCTTTCTAA-GACTA----TTTG

bdi-miR395d CTCATTGTTTCATTGCCGCTGTTT-ATA-AGTGTGCATGGAGCTGGAGAGCCGTGCACAG

bdi-miR395e CTCCTTGTTGCATTGCCACTGTTTTATA-AGTGTGCTTGGGGCCAGAGAGCTGTGCACAG

** * * ** * *

bdi-miR395c TGAAGTGTTTGGGGGAACTCTTGGTGTCACCA--------

bdi-miR395n TGAAGTGTTTGGGGGAACTCTTGGTGTCACCA--------

bdi-miR395a TGAAGTGTTTGGGGGAACTCTTGATGTCACCA--------

bdi-miR395l TGAAGTGTTTGGGGGAACTCTTGGTGTCACCAAGC-----

bdi-miR395i TGAAGTGTTTGGGGGAACTCTTGGTGTTAC----------

bdi-miR395k TGAAGTGTTTGGGGGAACTCTTGGTGTTAC----------

bdi-miR395b TGAAGTGTTTGGGGGAACTCTTGGTGTCACC---------

bdi-miR395h TGAAGTGTTTGGGGGAACTCTTGGT---------------

bdi-miR395j TGAAGTGTTTGGGGGAACTCTTGGTCTCACC---------

bdi-miR395m TGAAGTGTTTGGGGGAACTCTTGATATCACCAAGC-----

bdi-miR395g TGAAGTGTTTGGGGGAACTCTTGATGTCACC---------

bdi-miR395f TGAAGTGTTTGGGGGAACTCTCGGTGTCACCAAGC-----

bdi-miR395d TGAAGTGTTTGGGGGAACTCCGGGTGGCAACCAACAATAC

bdi-miR395e TGAAGTGTTTGGGGGAACTCTGGGTGGCAACCAGC-----

******************** * *

**(b)**

bdi-miR395c --------------------------TGGTATTACCATGAGTTCCTTT----GCAAGCAT

bdi-miR395n --------------------------TGGTATTACCATGAGTTCCCT-----GCAAGCAC

bdi-miR395a --------------------------TGGTATTATCATGAATTCCCT-----ACAAGCAC

bdi-miR395l -----------------------GTTTGGTATTATCATGAGTTCCCT-----GCAAGCAC

bdi-miR395i ----------------------------GTAATACCATGAGTTCCCT-----GCAAGCAC

bdi-miR395k ----------------------------GTACTACCATGAGTTCCCT-----GCAAGCAC

bdi-miR395b --------------------------TGGTATTACCATGAGTTCCCT-----GCAAGCAC

bdi-miR395h ---------------------------------ACCATGAGTTCCCT-----GCAAGCAC

bdi-miR395j ---------------------------GGTATTCTCATGAGTTTCCC-----GCAAGCAC

bdi-miR395m -----------------------GTTTGGAATTATCATGAGTTCCTT-----GCAAAAAC

bdi-miR395g ---------------------------GGTGTTACCAGGAGTTTCCT-----GCAAACAC

osa-miR395d ----------------------------GTATTGTCGTGAGTTCCCT-----TCAAGCAC

osa-miR395e ----------------------------GTATTATCGAGAGTTCCCT-----TCAACCAC

osa-miR395f -------------------------------TTATCGCGGGTTCCCT-----TCAATCAC

osa-miR395w --------------------------------------GAGTTCTCT-----TTAATCAT

osa-miR395r --------------------------------------GAGTTCCCT-----TCAACCAC

osa-miR395g ----------------------------GTATCACCGTGAGTTCCCT-----TCGAACAC

osa-miR395s ----------------------------GTATCACCGTGAGTTCCCT-----TCAAGCAC

bdi-miR395f -----------------------GTTTGGTGTCCCCGGGAGTTCCCT-----TCAAACAC

osa-miR395b ----------------------------GAGTCCCTAGGAGTTCCTT-----TCAAGCAC

osa-miR395u ----------------------------ACACTGCCAGGAATTCCCT-----TCAAGCAA

osa-miR395n --------------------------------------GAGTTCCCT-----TCAAGCAC

osa-miR395c ----------------------------GTATTATCAAGAGTTCTCT-----TTAAGCAC

osa-miR395o --------------------------------------GAGTTCTCT-----TTAAGCAC

osa-miR395v --------------------------------------GAATTCTCT-----TTAAGCAC

osa-miR395h ----------------------------TTGTTACCTGGAGTTTCCT-----CAACACAC

osa-miR395q --------------------------------------GAGTTCCCT-----TCAAGCAC

osa-MIR395p --------------------------------------GAGTTCCCT-----TCAAGCAC

osa-miR395j ----------------------------GTGTTCCCAAGAGTTCCTT-----GCAAGCAC

osa-miR395l --------------------------------------GCGTTCCTT-----CCAAGCAC

osa-miR395i ----------------------------GTTTTACCGGGAGTTCTCT-----TCAAGCAC

osa-miR395k ----------------------------GTTTTATCGGGAGTTTCCT-----TCAAGCAC

bdi-miR395d -----TTGTCAACTGGAGTTCTCCTCAAATCACTTCAGTAGCTAGCTAGCTAGCTTGTGC

bdi-miR395e GTTGGTTGTCACCTGGAGTTCTCCTCAAATCACTTCAGTAGCTAGCTA----GCTTGTGC

osa-miR395a -----TTGTCCACTGGAGTTCTCCTCAATCCACTTCAGTAGATAGCTAT--GGCTAG-GC

osa-miR395m ---------------GAGTTCTCCTCAAATCACTTCAGTAGATAGCTA----GCTAG-GC

osa-miR395t -----TTATCCACTGGAGTTCTCCTCAAACCACTTCAGCAGATAGCTA----GCTAG-GC

*

bdi-miR395c TTCACGTACG--A-GGCACTATCCCAA---------------GGGCTATT----------

bdi-miR395n TTCACC------A-TGCACTACCCCAA---------------GGGCTATT----------

bdi-miR395a TTCACA------A-GAAGCTATTCCTA---------------GGGCTATT----------

bdi-miR395l TTCACG------A-GGCACTATCCCAA---------------GGGCTACT----------

bdi-miR395i TTCATG------A-GGCCTAATTCTAA---------------GGGCTACT----------

bdi-miR395k TTCATG------A-GGCCCAATTCTGA---------------GGGCTACT----------

bdi-miR395b TTCACG------A-GGCCCAATTATGA---------------TGGCTACT----------

bdi-miR395h TTCACG------A-GGCCGTTTTCTGA---------------GGGCTACT----------

bdi-miR395j TTCACG------A-GGCCGTTTTCTGA---------------GGGCTACT----------

bdi-miR395m TTCACG------A-GGTCCAATTCTAA---------------GTGCTCCC----------

bdi-miR395g TTCACG------A-GTGCCTATTTTAAT--------------GGGTTGTT----------

osa-miR395d TTCACG------T-GGCACTATCTCAA---------------TGCCTACTA---------

osa-miR395e TTCACG------T-GGCACTGTTTCAA---------------GGCCTATTG---------

osa-miR395f TTCACA------T-GGTACTATTTCAA---------------GGCCTACTA---------

osa-miR395w TTCACA------T-GGCACTATTTTAA---------------GGCCTACTG---------

osa-miR395r TTCACG------T-GGCACTATTTTAG---------------GACCTACTA---------

osa-miR395g TTCACG------T-GGCACTATTTCAA---------------TGCCTATT----------

osa-miR395s TTCACG------T-GGCACTATTTCAA---------------TGCCTATT----------

bdi-miR395f TTTACG------A-AGCATCTTTCTAA---------------GAC-TATT----------

osa-miR395b TTTACG-ACA--C-AC---TGTATTGA---------------GAGTTGTC----------

osa-miR395u TTCATG-AAA--C-AA---TATTTTGA---------------GAGTTGTT----------

osa-miR395n TTCACG-ACG--C-AC---TATTTAGA---------------GAGTTGTT----------

osa-miR395c TTCATA-CGA--C-ACCATTATTTATA---------------GGGTTGTT----------

osa-miR395o TTCATA-CGA--C-ACCATTATTGTTA---------------GGGTTGTT----------

osa-miR395v TTCATA-CGA--C-ACAATTATTTCAA---------------GGGTTGTT----------

osa-miR395h TTCACATCTGCTA-GGCCCTATTACAATTGCGCAATGT--GGAGTCTGC--------AAT

osa-miR395q TTCACAT-------GACACTATTTCAAT---------------GTCTAT--------TAT

osa-MIR395p TTCACGT-------GGCACTATTTCAAT---------------GCGTAC--------CGT

osa-miR395j TTCACATAG---A-ACTTCTGTTACTCTCATGTAACATTGGGAACTTGA-------GAAG

osa-miR395l TTCACACAG---A-GCTTTTATTTCTCTCACATCG-ATTGAGAACTTAATTA----GAAG

osa-miR395i TTCACGTAG---A-GCTTTC----TATTGACAT-------GGAGCTTTA-------GAA-

osa-miR395k TTCACGTAG---A-GCTTTC----TATTGATAT-------GGAGCTTTG-------GAA-

bdi-miR395d CTCATTGTTTCATTGCCGCTGTTT-ATAAGTGTG----CATGGAGCTGGAG---AGCCGT

bdi-miR395e CTCCTTGTTGCATTGCCACTGTTTTATAAGTGTG----CTTGGGGCCAGAG---AGCTGT

osa-miR395a CTCATTGCATTGC-AC---TGTTACATAACTGTGA--TCATGGGGCCAAAAGCTAGCTAT

osa-miR395m TTCATTGCATTAC-AC---TGTTACAAAACTGTGA--GCATGGGGCCAAAAGCTAGTTGT

osa-miR395t CTCATTGCATTGC-ACCACTGTTGCATAACTATGA--GCATGGGGCCAAAAGTTAGCTGC

*

bdi-miR395c -----GTGAAGTGTTTGGGGGAACTCTTGGTGTCACCA--------

bdi-miR395n -----GTGAAGTGTTTGGGGGAACTCTTGGTGTCACCA--------

bdi-miR395a -----GTGAAGTGTTTGGGGGAACTCTTGATGTCACCA--------

bdi-miR395l -----GTGAAGTGTTTGGGGGAACTCTTGGTGTCACCAAGC-----

bdi-miR395i -----GTGAAGTGTTTGGGGGAACTCTTGGTGTTAC----------

bdi-miR395k -----GTGAAGTGTTTGGGGGAACTCTTGGTGTTAC----------

bdi-miR395b -----GTGAAGTGTTTGGGGGAACTCTTGGTGTCACC---------

bdi-miR395h -----GTGAAGTGTTTGGGGGAACTCTTGGT---------------

bdi-miR395j -----GTGAAGTGTTTGGGGGAACTCTTGGTCTCACC---------

bdi-miR395m -----GTGAAGTGTTTGGGGGAACTCTTGATATCACCAAGC-----

bdi-miR395g -----GTGAAGTGTTTGGGGGAACTCTTGATGTCACC---------

osa-miR395d ----TGTGAAGTGTTTGGGGGAACTCTCGATATCAC----------

osa-miR395e ----TGTGAAGTGTTTGGGGGAACTCTCGATATCAC----------

osa-miR395f ----TGTGAATTGTTTGGGGGAACTCTCGATGT-------------

osa-miR395w ----TGTGAAGTGTTTGGGGGA-TTCTC------------------

osa-miR395r ----TGTGAAGTGTTTGGGGGAACTC--------------------

osa-miR395g -----GTGAAGTGTTTGGGGGAACTCTCGATGTCAT----------

osa-miR395s -----GTGAAGTGTTTGGGGGAACTCTCGATGTTCC----------

bdi-miR395f ----TGTGAAGTGTTTGGGGGAACTCTCGGTGTCACCAAGC-----

osa-miR395b -----GTGAAGTGTTTGGGGGAACTCTTAGTGTCGC----------

osa-miR395u -----GTGAAGCGTTTGGGGGAAATCTCAGTGTCGC----------

osa-miR395n -----GTGAAGTGTTTGGGGGAACTC--------------------

osa-miR395c -----GTGAAGTGTTTGGAGGAACTCTCGGTGTCAT----------

osa-miR395o -----ATGAAGTGTTTGGAGGAACTC--------------------

osa-miR395v -----GTGAAGTATTTGGCGGAACTC--------------------

osa-miR395h TGGTAGTGAAGTGTTTGGGGGAACTCTAGGTGGCAC----------

osa-miR395q -----GTGAAGTGTTTGGGGGAACTC--------------------

osa-MIR395p -----GTGAAGTGTTTGGGGGAACTC--------------------

osa-miR395j CTACTGTGAAGTGTTTGGGGGAACTCTAGGTGGCAC----------

osa-miR395l CTTTTGTGAAGTGTTTGGGGGAACTC--------------------

osa-miR395i -CAATGTGAAGTGTTTGGGGGAACTCTTGGTACCAA----------

osa-miR395k -CAATGTGAAGTGTTTGGGGGAACTCTTGATACCAA----------

bdi-miR395d GCACAGTGAAGTGTTTGGGGGAACTCCGGGTGGCAACCAACAATAC

bdi-miR395e GCACAGTGAAGTGTTTGGGGGAACTCTGGGTGGCAACCAGC-----

osa-miR395a GTATAGTGAAGTGCTTGGGGGAACTCCAGTTGACAC----------

osa-miR395m ATATAGTGAAGTGTTTGGGGGAACTC--------------------

osa-miR395t TTATAGTGAAGTGTTTGGGGAAACTCCGGTTGGCAA----------

**** **** * * **

**Additional file 4. Multiple sequence alignment of miR395 genes in *Brachypodium* (a) and comparison with their homologs in rice (b).**

The alignment was obtained with the ClustalW 1.83 program (http://align.genome.jp) using the ID weight matrix. The miR395 precursor sequences in rice were obtained from the miRBase (<http://microrna.sanger.ac.uk/>).
