# Supplementary material for: Deep sequencing of Brachypodium small RNAs at the global genome level identifies microRNAs involved in cold stress response
Source: BMC Genomics. 2009 Sep 23;10:449. doi: 10.1186/1471-2164-10-449 (PMC2759970; doi:10.1186/1471-2164-10-449)
Supplement: Additional file 5 — The secondary structures of cold-responsive predicted Brachypodium miRNAs. is a figure showing the secondary structure of cold-responsive predicted Brachypodium miRNAs. [file 1471-2164-10-449-S5.pdf]

bdi-miR901T (ΔG = -66.00)

```
           10         20         30         40         50
      AUU-                                        AUAUUU
UCUCUC    UAUUCUAUGCCAUGUCGUCACAUAUCCUACAUGGCA        \
AGAGAG    GUAAGAUACGGUGUAGUAGUGUAUAGGAUGUAUCGU        A
      GAGC                                        ACAACC
    100        90         80         70         60
```

sRNA (5' to 3' orientation) mapped to this predicted precursor hairpin:

in the NC library:

UAUGCCAUGUCGUCACAUAUC (sequencing frequency: 3)

in the WC library:

UAUGCCAUGUCGUCACAUAUC (sequencing frequency: 24)

bdi-miR902T (ΔG = -86.50)

```
         10        20        30        40        50        60              70        80
         U              G   UA       GA                 UG           --AA            UAGUAA
CAUUAGAUC UUAAAUAAACGGAU AGA  AAAGGUG  ACCUCUAAUCACUUAAA  CAUUUUAUGA     AAAAAAACAC      \
GUAAUCUAG AGUUUAUUUGCCUA UCU  UUUCCAC  UGGAGAUUAGUGAAUUU  GUAAAAUACU     UUUUUUUGUG      A
         -              A   UC       UC                 UC           CUUC           CACCCU
     170        160       150       140       130       120       110       100        90
```

sRNA (5' to 3' orientation) mapped to this predicted precursor hairpin:

in the NC library:

UAGAUCUUUAAAUAAACGGAUG (sequencing frequency: 4)

in the WC library:

UAGAUCUUUAAAUAAACGGAUG (sequencing frequency: 22)

bdi-miR903T (ΔG = -63.8)

```
            10         20         30         40
                          G         -- G   GG  AG
  CCGAGCUCCCUCAAUCUCUUUUCC CUUUCUGCU  U GCU  AA  \
  GGCUCGAGGGAGUUAGAGAAAAGG GAGAGACGA  G UGG  UU  G
                          G         UA G   GG  GA
    90       80        70          60       50
```

sRNA (5' to 3' orientation) mapped to this predicted precursor hairpin:

in the NC library:

GGGGAAAAGAGAUUGAGGGAG (sequencing frequency: 6)

in the WC library:

GGGGAAAAGAGAUUGAGGGAG (sequencing frequency: 31)

bdi-miR904T (ΔG = -68.70)

```
        10        20        30        40        50          60        70
    U    ACGC            A                U           U  C--   UCUA   CAU
AUAC UAAA    UUAGAGAUGGGC AAGUGCUAUCAAUUGU UGAACAAACUU GC   CAG    CGA   \
UAUG AUUU    AAUCUCUACCCG UUCACGAUAGUUGGCA ACUUGUUUGAG CG   GUC    GCU   U
    C    GACA            A                U           U  UUA   CAA-   CAG
    140       130       120       110       100        90        80
```

sRNA (5' to 3' orientation) mapped to this predicted precursor hairpin:

in the NC library:

UGUUCAUACGGUUGAUAGCAC (sequencing frequency: 3)

in the WC library:

UGUUCAUACGGUUGAUAGCAC (sequencing frequency: 22)

bdi-miR905T (ΔG = -54.19)

```
        10          20          30          40          50          60            70
         U            C     C   AAAAUAG     UA  AUAUAUAUAU     A       UG   UC      UU  U  UCG
UAUUGUUUU CUUUGACCGAGC UUUGA CAA       UCUAU  AU          UGUGA AUAAAUU  AUA  AUUGUG  CG GU   A
AUAACAAAA GAAACUGGCUUG AAACU GUU       AGAUA  UG          ACACU UGUUUAG  UAU  UGAUAU  GU CA   A
         -            C     A   ACUAAUA     GC  CACACUGAAU     A       --   --      UU  U  UGA
140          130          120              110          100          90              80
```

sRNA (5' to 3' orientation) mapped to this predicted precursor hairpin:

in the NC library:

UUCUUUGACCGAGCCUUUGAC (sequencing frequency: 7)

in the WC library:

UUCUUUGACCGAGCCUUUGAC (sequencing frequency: 39)

bdi-miR906T (ΔG = -47.73)

```
        10        20           30         40       50
             C         G    --       ACUU      ACCCCCC    UUGA
UCCGAAAUAAACC GCAGUCCAC UUCU   AUUGGA     UUGCG        UCC     \
AGGCUUUAUUUGG CGUCAGGUG AAGA   UAAUUU     GACGC        AGG     U
             A         -    GC       GCAC      GCAAUUA    CUUU
       110       100         90        80         70          60
```

sRNA (5' to 3' orientation) mapped to this predicted precursor hairpin:

in the NC library:

UGGACUGCAGGUUUAUUUCGG (sequencing frequency: 4)
in the WC library:
UGGACUGCAGGUUUAUUUCGG (sequencing frequency: 23)

bdi-miR910T (ΔG = -47.10)

```
         10        20        30        40
                        G   CA    C     CACC
 UAAAGCCUUAGGGAGUAUCUACA UGG  AGUA UUUUC    A
 AUUUCGGAAUCUCUCAUAGAUGU ACC  UCAU AGAAG    A
                        G   A-    A     AAAG
        80        70        60        50
```

sRNA (5' to 3' orientation) mapped to this predicted precursor hairpin:
in the NC library:
UGUAGAUACUCUCUAAGGCUU (sequencing frequency: 28)
in the WC library:
UGUAGAUACUCUCUAAGGCUU (sequencing frequency: 5)

bdi-miR911T

super_277:408-428 **(ΔG = -60.20)**

```
        10        20        30        40
                           A  A          A
UACUCCCUCCGUUCCUAAAUUCUUGUC UG UUUUAGUUCA A
AUGAGGGAGGCAGGGAUUUAAGAACAG AC AAAAUCAAGU U
                           C  C          U
      80        70        60        50
```

sRNA (5' to 3' orientation) mapped to this predicted precursor hairpin:
in the NC library:
AAGAAUUUAGGGACGGAGGGA (sequencing frequency: 35)
in the WC library:
AAGAAUUUAGGGACGGAGGGA (sequencing frequency: 4)

super_2:20949532-20949552 **(ΔG = -39.60)**

```
        10        20        30        40
          G          C     CG GA   U   U  A
GUAUUCCCUC GUUCCUAAAU CUUGU  U  UUU AGU CA A
CAUGAGGGAG CAGGGAUUUA GAACA  G  AAA UCA GU U
          G          A     AU GC   U   U  U
       80        70        60        50
```

sRNA (5' to 3' orientation) mapped to this predicted precursor hairpin:

in the NC library:

AAGAAUUUAGGGACGGAGGGA (sequencing frequency: 35)

in the WC library:

AAGAAUUUAGGGACGGAGGGA (sequencing frequency: 4)

bdi-miR912T (ΔG = -40.40)

```
         10        20        30       40        50
          AU-    G   AU       U  AC        AU    AGG
 AGGGUUUU   UAGCU CCG  UCAUCCA UC  CUACCAAGA  CAUG   G
 UUUCGAAA   AUCGA GGC  GGUAGGU AG  GAUGGUUCU  GUAU   C
          AAC    G   AC       C  CC        --    AUG
     100      90         80       70        60
```

sRNA (5' to 3' orientation) mapped to this predicted precursor hairpin:

in the NC library:

ACUGGAUGGCACGGGAGCUAC (sequencing frequency: 67)

AGCUGCCGAUUCAUCCAUUCA (sequencing frequency: 4)

UGGAUGGCACGGGAGCUAC (sequencing frequency: 1)

in the WC library:

ACUGGAUGGCACGGGAGCUAC (sequencing frequency: 7)

bdi-miR913T

super_1:16079568-16079588 (ΔG = -56.50)

```
           10        20        30        40        50         60
         -    CG  G       C          CA    A     AG-        UGAC
UGACCCUCA GGUA  CU UGACCCU CUUAGUUCAA  AAAU AACUA   UUUUAAAA    A
ACUGGGAGU CCAU  GA ACUGGGA GAAUCAAGUU  UUUA UUGAU   GAAAUUUU    C
         C    AG  A       A          UG    C     CAA        UCAA
        120       110       100        90        80        70
```

sRNA (5' to 3' orientation) mapped to this predicted precursor hairpin:

in the NC library:

UUUGAACUAAGAAGGGUCAAA (sequencing frequency: 43)

in the WC library:

UUUGAACUAAGAAGGGUCAAA (sequencing frequency: 5)

super_3:15856277-15856297 (ΔG = -66.70)

```
         10        20        30        40        50        60
    AC         -  C         C          C           AAA        UGAC
GUG  CCUCAGGGU AC UUUGACCCU CUUAGUUCAA CAAAUGAACUA   UUUUAAAA    A
CAC  GGAGUCCCA UG AAACUGGGA GAAUCAAGUU GUUUACUUGAU   AAAGUUUU    U
    CA         U  U         A          U           CAA        UCAA
   130        120       110      100      90       80        70
```

sRNA (5' to 3' orientation) mapped to this predicted precursor hairpin:
in the NC library:
UUUGAACUAAGAAGGGUCAAA (sequencing frequency: 43)
in the WC library:
UUUGAACUAAGAAGGGUCAAA (sequencing frequency: 5)

super_7:14469193-14469213 (ΔG = -75.70)

```
          10          20          30          40          50          60
                 C          CCA         C            AAG         UGAC
GUGACCCUCAGGGUACC UUUGACCCU   UAGUUCAA CAAAUGAACUA   UUUUAAAA     A
CACUGGGAGUUCCAUGG AAACUGGGA   AUCAAGUU GUUUACUUGAU   AAAGUUUU     C
                 A          AGA         U            CAA         UUAA
  130         120         110         100         90          80          70
```

sRNA (5' to 3' orientation) mapped to this predicted precursor hairpin:
in the NC library:
UUUGAACUAAGAAGGGUCAAA (sequencing frequency: 43)
in the WC library:
UUUGAACUAAGAAGGGUCAAA (sequencing frequency: 5)

bdi-miR914T (ΔG = -52.60)

```
          10            20          30          40          50
    G                                      AA       AU      UCA
 CC UCCAACCCUUCUUAGUUCAAGCAAAUGGAUUA    UUUUA  CACA     \
 GG AGGUUGGGAGGAAUCGAGUUUGUUUACUUGAU    AAAAU  GUGU     G
   -                                       C--      --      UUU
 100        90          80          70          60
```

sRNA (5' to 3' orientation) mapped to this predicted precursor hairpin:
in the NC library:
UUGAGCUAAGGAGGGUUGGAG (sequencing frequency: 37)
in the WC library:
UUGAGCUAAGGAGGGUUGGAG (sequencing frequency: 6)

bdi-miR915T (ΔG = -74.10)

```
          10          20          30          40          50
   -                        C             UU      CA      UG
 CC CAUUGACUCUCCUUAGUUCAA CAAAUGAACUGG  UUUU    AAAGU  U
 GG GUAACUGGGAGGAAUCAAGUU GUUUACUUGAUU  AAAA    UUUUA  G
   A                        A             UU      --      CU
    100        90          80          70          60
```

sRNA (5' to 3' orientation) mapped to this predicted precursor hairpin:

in the NC library:

UUGAACUAAGGAGGGUCAAUG (sequencing frequency: 29)

in the WC library:

UUGAACUAAGGAGGGUCAAUG (sequencing frequency: 5)

bdi-miR916T (ΔG = -59.60)

```
          10               20        30        40
     C            C         G        C    UCC
 CACU UGAUCAUUUGCC CGUCUUGUA CACUGACA GUGG   A
 GUGA ACUAGUAAACGG GCGGGACAU GUGACUGU CACC   U
     A            A         A        A    CCG
  90       80        70        60        50
```

sRNA (5' to 3' orientation) mapped to this predicted precursor hairpin:

in the NC library:

AGGGCGAGGCAAAUGAUCAAA (sequencing frequency: 33)

in the WC library:

AGGGCGAGGCAAAUGAUCAAA (sequencing frequency: 6)

bdi-miR917T (ΔG = -51.70)

```
          10        20          30        40
     U           A          CA      -      AA
 AUAC CCCUCCGUCCC AAAUAAGUGA  UGGAUU GUAUA  A
 UAUG GGGAGGCAGGG UUUAUUCACU  ACCUAA CAUAU  A
     C           C          AC      A      CU
         80        70        60       50
```

sRNA (5' to 3' orientation) mapped to this predicted precursor hairpin:

in the NC library:

ACUUAUUUCGGGACGGAGGGC (sequencing frequency: 34)

in the WC library:

ACUUAUUUCGGGACGGAGGGC (sequencing frequency: 6)

bdi-miR918T (ΔG = -74.70)

```
         10        20        30          40        50                60        70        80
  C        U   A  G  G            A-   C--         AA---------   CCAU    G  CCG    ACGAGA   AA  G
GC AGGACCGC GAC UG UA UCGAUUUUCUCA  GAU   UUUUUGCAA           UCA    GCCC AG   CCCC      GGC  GC G
CG UCCUGGCG CUG AC AU AGCUAAAAGAGU  CUA   AAAAACGUU           AGU    CGGG UC   GGGG      CCG  CG C
  U        C   -  G  G            CC   AAA         GCCGCACCAAG   CC--    G  CUA    CAGAC-   --  C
     180      170        160       150       140       130       120         110       100      90
```

sRNA (5' to 3' orientation) mapped to this predicted precursor hairpin:

in the NC library:

AAAAUCGAGUAGCAGUCCGCG (sequencing frequency: 41)

CUGACAUGGUAGUCGAUUUUC (sequencing frequency: 3)

AUCGAGUAGCAGUCCGCGGUC (sequencing frequency: 1)

in the WC library:

AAAAUCGAGUAGCAGUCCGCG (sequencing frequency: 5)

bdi-miR919T (ΔG = -108.70)

```
        10        20        30        40        50        60        70        80        90
     A   G        AC                  A         G     CGA   G  U        U      UU       CU
GGCUU GGC AGGGCCGC  GCUGCUAUUCGAUUUUCUCA GGGUUUUUU UGCAA  CGU GU CUCGGGCC CCCCGU  GGGGCAG  \
UCGAA CCG UCCUGGUG  CGACGAUGAGCUAAAAGAGU CUUAAAAAA ACGUU  GCG UA GGGCUCGG GGGGCG  CCCCGUU  G
     G   G        CC                  -         -     UUA   G  C        C      CU       CC
     180      170       160       150       140       130       120       110       100
```

sRNA (5' to 3' orientation) mapped to this predicted precursor hairpin:

in the NC library:

AAAAUCGAGUAGCAGCCCGUG (sequencing frequency: 144)

AAAAUCGAGUAGCAGCCCGU (sequencing frequency: 1)

in the WC library:

AAAAUCGAGUAGCAGCCCGUG (sequencing frequency: 28)

bdi-miR920T (ΔG = -99.60)

```
        10        20        30        40        50        60        70        80        90
          G     U         CA            UGA            CCC         C    U         AA
 GCCAUAUCUU GGCUC AGGUAGGUU  AUCCUCAAUUGAA  CGAAGGAAUUAAA  AGCAGCAAA AAAC GAAUUACC  \
 UGGUAUAGAA CCGAG UCCAUCCAA  UAGGAGUUAACUU  GCUUCCUUAAUUU  UCGUCGUUU UUUG CUUUAAUGG  A
          A     U         UA            U--            AGC         -    U         UU
  170       160       150       140           130       120        110       100
```

sRNA (5' to 3' orientation) mapped to this predicted precursor hairpin:

in the NC library:

AUCUUGGGCUCUAGGUAGGUU (sequencing frequency: 25)

in the WC library:

AUCUUGGGCUCUAGGUAGGUU (sequencing frequency: 5)

bdi-miR921T (ΔG = -110.30)

```
       10        20        30        40        50        60        70        80         90       100
               A             CUAUAAA          C   C   C            -    A      A     A   -     G  C   AG
UUUUUUUCGGCUUCU GGACCGGCUUCUC       CUGCUCUCAC UAG UUC UGGAGAAGCCGC AUC  AGAUUU GUUAG  CU CCAAA UA UUU  \
AAAAAAAGCCGAAGG CCUGGCCGAAGAG       GACGGGAGUG GUC AAG ACCUCUUCGGCG UGG  UUUAAA UAAUC  GA GGUUU AU AAA  A
               A             ACUCUUC          A   A   U            G    A      C     C   C     G  U   CC
   200        190       180       170       160       150       140       130       120        110
```

sRNA (5' to 3' orientation) mapped to this predicted precursor hairpin:

in the NC library:

UUUCGGCUUCUAGGACCGGCU (sequencing frequency: 15)

in the WC library:

UUUCGGCUUCUAGGACCGGCU (sequencing frequency: 3)

bdi-miR922T ($\Delta G = -66.80$)

```
        10        20        30        40
                           C     U       AG
GUACUCCCUCCGUCCCAAAAUAAGUGA GUGGA UUGUAUA  A
CAUGAGGGAGGCAGGGUUUUGUUCACU CACCU AACAUAU  A
                           A     U       CU
       80        70        60        50
```

sRNA (5' to 3' orientation) mapped to this predicted precursor hairpin:

in the NC library:

ACUUGUUUUGGGACGGAGGGA (sequencing frequency: 22)

in the WC library:

ACUUGUUUUGGGACGGAGGGA (sequencing frequency: 4)

bdi-miR923T ($\Delta G = -66.30$)

```
        10       20        30         40          50         60
                 A  U   G      C              GA         UUG
UAAGCCACAAAAGCACC AA UAG UGCUUU GGCUUUGGCUUUU    CAUUUGAC   A
AUUCGGUGUUUUCGUGG UU AUC ACGAAA CUGAAACCGAAAA    GUAGAUUG   U
                 A  U   A      A              --         UUA
      110       100        90          80        70
```

sRNA (5' to 3' orientation) mapped to this predicted precursor hairpin:

in the NC library:

UUAGGUGCUUUCGGCUUUGGC (sequencing frequency: 32)

in the WC library:

UUAGGUGCUUUCGGCUUUGGC (sequencing frequency: 6)

bdi-miR924T (ΔG = -58.20)

```
          10          20          30          40          50
      --              C           C  GU            A
AAAUCU  UACUCCCUCCGAC CAUAUUACUUG CG GAUUUAGUGCA C
UUUAGA  AUGGGGGAGGCUG GUAUAAUGAAC GC CUAAAUCAUGU U
      UC              U           A  GU            U
100         90          80         70         60
```

sRNA (5' to 3' orientation) mapped to this predicted precursor hairpin:

in the NC library:

AGUAAUAUGUGUCGGAGGGGG (sequencing frequency: 32)

in the WC library:

AGUAAUAUGUGUCGGAGGGGG (sequencing frequency: 5)

bdi-miR925T (ΔG = -64.40)

```
         10        20        30        40        50
                                       AAAAA   UA
GUCGACGAUUUAACUCACGUAACAUAAGUUUGAACUCAC     GUA  U
UAGCUGCUAAAUUGAGUGCAUUGUAUUCAAACUUGAGUG     UAU  C
                                       CAUUG   UA
100      90        80        70        60
```

sRNA (5' to 3' orientation) mapped to this predicted precursor hairpin:

in the NC library:

UUACGUGAGUUAAAUCGUCGA (sequencing frequency: 16)

in the WC library:

UUACGUGAGUUAAAUCGUCGA (sequencing frequency: 3)

bdi-miR926T (ΔG = -39.20)

```
        10        20        30        40
                    A   AUC  CA          A
UACUCCCUCUGUUUCUAAGU CUU   GU  UUUUAGUUCA A
AUGAGGGAGGUAAGGAUUUA GAA   CA  AGAAUCAAGU U
                    A   CGA  CC          U
      80        70        60        50
```

sRNA (5' to 3' orientation) mapped to this predicted precursor hairpin:

in the NC library:

AAGAAUUUAGGAAUGGAGGGA (sequencing frequency: 36)

in the WC library:

AAGAAUUUAGGAAUGGAGGGA (sequencing frequency: 6)

bdi-miR927T (ΔG = -50.10)

```
            10         20         30         40
     C        G                 UG   G        U   A
 GUA  UCCCUCC  UUCCUAAAUUCUUGU   UG  UUUUAGU  CA  A
 CAU  AGGGAGG  AGGGAUUUAAGAACA   AC  AAAAUCA  GU  U
     U        A                 GU   G        U   U
        80          70         60         50
```

sRNA (5' to 3' orientation) mapped to this predicted precursor hairpin:
in the NC library:
AGAAUUUAGGGAAGGAGGGAU (sequencing frequency: 37)
in the WC library:
AGAAUUUAGGGAAGGAGGGAU (sequencing frequency: 7)

bdi-miR928T (ΔG = -38.20)

```
          10          20          30
     A                       --    U     G
UUU   CUCCCUCCGGUCCAUAAUAA   AUA  GUAC  \
AAA   GAGGGAGGCCAGGUAUUAUU   UAU  CAUG  G
     C                       CA    U     A
  70          60          50          40
```

sRNA (5' to 3' orientation) mapped to this predicted precursor hairpin:
in the NC library:
ACUUAUUAUGGACCGGAGGGA (sequencing frequency: 24)
in the WC library:
ACUUAUUAUGGACCGGAGGGA (sequencing frequency: 3)

Additional data file 5. The secondary structures of cold-responsive predicted *Brachypodium* miRNAs. Sequences indicated in red and green color correspond to identified miRNAs and miRNA* sequences respectively.
