# Supplementary material for: Deep sequencing of Brachypodium small RNAs at the global genome level identifies microRNAs involved in cold stress response
Source: BMC Genomics. 2009 Sep 23;10:449. doi: 10.1186/1471-2164-10-449 (PMC2759970; doi:10.1186/1471-2164-10-449)
Supplement: Additional file 6 — The conservation of cold-responsive predicted Brachypodium miRNAs. is a table listing the identified homologs of cold-responsive predicted Brachypodium miRNAs in other plant species. [file 1471-2164-10-449-S6.doc]

**Additional data file 6**. **The conservation of cold-responsive putative *Brachypodium* miRNAs.**

| Name of miRNA | Plant species | | |
| --- | --- | --- | --- |
|  | Wheat | Barley | Rice |
| bdi-miR901T |  |  |  |
| bdi-miR902T |  |  |  |
| bdi-miR903T |  |  |  |
| bdi-miR904T |  |  |  |
| bdi-miR905T |  |  |  |
| bdi-miR906T |  |  |  |
| bdi-miR910T |  |  |  |
| bdi-miR911T | **+** |  | **+** |
| bdi-miR912T |  |  | **+** |
| bdi-miR913T |  |  | **+** |
| bdi-miR914T |  |  | **+** |
| bdi-miR915T |  |  |  |
| bdi-miR916T |  |  |  |
| bdi-miR917T |  |  |  |
| bdi-miR918T |  |  | **+** |
| bdi-miR919T |  |  |  |
| bdi-miR920T |  |  |  |
| bdi-miR921T |  |  |  |
| bdi-miR922T | **+** | **+** | **+** |
| bdi-miR923T |  |  |  |
| bdi-miR924T |  |  |  |
| bdi-miR925T |  |  |  |
| bdi-miR926T |  |  |  |
| bdi-miR927T |  |  |  |
| bdi-miR928T |  |  | **+** |

The plus symbols in the table indicate the identification of miRNA homologs in other plant species.
