# Supplementary material for: Deep sequencing of Brachypodium small RNAs at the global genome level identifies microRNAs involved in cold stress response
Source: BMC Genomics. 2009 Sep 23;10:449. doi: 10.1186/1471-2164-10-449 (PMC2759970; doi:10.1186/1471-2164-10-449)
Supplement: Additional file 7 — Real-time PCR validation of the cold-responsive expression of predicted miRNAs (miR901T and miR904T) in Brachypodium. is a figure showing the Real-time PCR analysis results of the levels of predicted Brachypodium miRNAs (miR901T and miR904T) in seedlings with and without cold-treatment. [file 1471-2164-10-449-S7.doc]

**
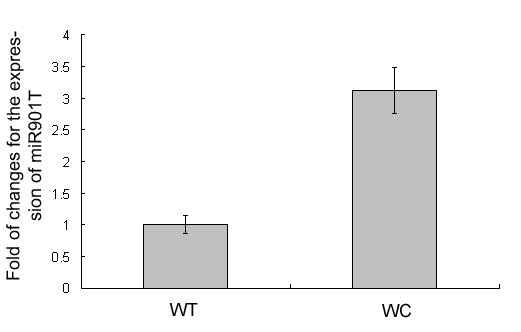
**

**
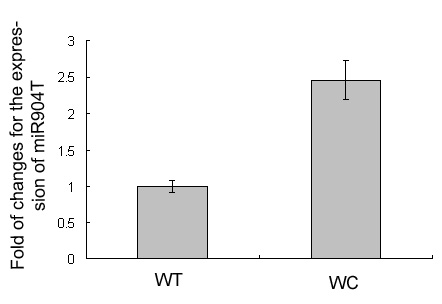
**

**Additional file 7. Real-time PCR validation of the cold-responsive expression of predicted miRNAs (miR901T and miR904T) in *Brachypodium*.**

The expression of *Brachypodium* miR901T and miR904T changed significantly (> 7 fold) after cold treatment, but their cold-responsive expression can not be validated by RNA gel blot analysis because of their low expression level. Real-time PCR has been employed to quantify the levels of miR901T and miR904T in *Brachypodium* seedlings with (WC) and without (WT) cold treatment (4°C for 24 hour). Real-time PCR analysis was performed as described by Lu *et al.* (Plant J., 2008, 55:131-151). The Poly(A) Tailing Kit (Ambion, Austin, TX, USA) was used to add Poly(A) tails for the total RNA and the Taqman reverse transcription reagents (Applied Biosystems, Foster City, CA, USA) as well as the oligo(dT) 3’-RACE adaptor [5’-GCGAGCACAGAATTAATACGACTCACTATAGG(T)_12_VN-3’, in the FirstChoice RLM-RACE kit; Ambion, Austin, TX, USA] were used for reverse transcription. The Real-time PCR was carried out using the 3’-RACE outer primer (5’-GCGAGCACAGAATTAATACGAC-3’) as the reverse primer and mature miRNA sequences as the forward primer. The miRNA levels were normalized to the level of 5.8S rRNA. The normalized miRNA levels in the WT samples were arbitrarily set to 1. Error bars represent the standard deviation of three PCR replicates of one reverse transcription reaction.

The forward primers used were listed as below:

miR901T: 5’-TATGCCATGTCGTCACATATC-3’;

miR904T: 5’-TGTTCATACGGTTGATAGCAC-3’;

5.8S rRNA: 5’- CCTGCCTGGGCGTCACGC-3’.
