# Supplementary material for: Deep sequencing of Brachypodium small RNAs at the global genome level identifies microRNAs involved in cold stress response
Source: BMC Genomics. 2009 Sep 23;10:449. doi: 10.1186/1471-2164-10-449 (PMC2759970; doi:10.1186/1471-2164-10-449)
Supplement: Additional file 8 — Alignment of cold-responsive miRNAs (bdi-miR901T and bdi-miR397) identified in Brachypodium to their predicted target mRNAs. is a figure showing the alignment of cold-responsive miRNAs identified in Brachypodium to their predicted target mRNAs. [file 1471-2164-10-449-S8.doc]

(1)

**bdi-miR901T** 5’UAUGCCAUGUCGUCACAUAUC 3’

|||||| |||| |||||0|||

3’UCAGGUUUUCAUACGGGACAGGAGUGUGUAGUUGGUUGCAGAAUGUGGU 5’

**LOB domain protein** (Bradi1g13160.1)

(2)

**bdi-miR397** 5’AUUGAGUGCAGCGUUGAUGAA3’

||||||||| ||||||||||

3’ACCTCTTCTTCTCGAGCAGTAACTCACGGCGCAACTACTCCGCGTTTTCC 5’

**Laccase** (Bradi1g24880.1)

(3)

**bdi-miR397** 5’AUUGAGUGCAGCGUUGAUGAA 3’

||||| ||||||||||||||

3’ CUUCUUCUCGAGGUGUAACUGACGUCGCAACUACUACGCCUCCUCCAUGCAG 5’

**Laccase** (Bradi2g55060.1)

(4)

**bdi-miR397**  5’AUUGAGUGCAGCGUUGAUGAA 3’

||||| |||||||||||||

3’GCUUCUCGAGGCACAACUCCCGCCGCAACUACUUGGCCUCUUCCAACCAG 5’

**Laccase precursor** (Bradi4g44810.1)

**Additional file 8. Alignment of cold-responsive miRNAs (bdi-miR901T and bdi-miR397) identified in *Brachypodium* to their predicted target mRNAs.**
