# Supplementary material for: Deep sequencing of Brachypodium small RNAs at the global genome level identifies microRNAs involved in cold stress response
Source: BMC Genomics. 2009 Sep 23;10:449. doi: 10.1186/1471-2164-10-449 (PMC2759970; doi:10.1186/1471-2164-10-449)
Supplement: Additional file 9 — Real-time PCR analysis for the expression of predicted miRNA target genes in Brachypodium after cold-treatment. is a figure showing the results of the Real-time PCR analysis for the expression of predicted miRNA target genes in Brachypodium seedlings with and without cold-treatment. [file 1471-2164-10-449-S9.doc]

**
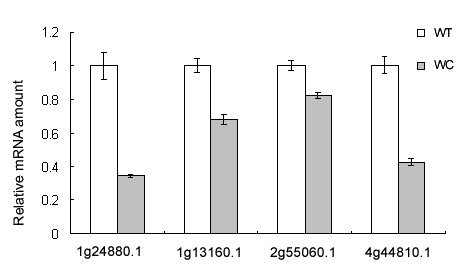
**

**Additional file 9. Real-time PCR analysis for the expression of predicted miRNA target genes in *Brachypodium* after cold-treatment.**

Total RNA was isolated from 7-day-old *Brachypodium* seedlings with (WC) and without (WT) cold treatment. Real-time PCR analysis was performed as described by Dai *et al*. (Plant Physiol., 2007, 143:1739-1751). The SuperScripts II reverse transcriptase (Invitrogen, Carlsbad, CA, USA) was used for reverse transcription and SYBR Green PCR Master mix (Applied Biosystems, Foster City, CA, USA) was used for quantitative PCR. The amplification of a *Brachypodium* *Tubulin* gene was used as an internal control to normalize all data. The normalized miRNA levels in the WT samples were arbitrarily set to 1. Quantitative PCR reactions were repeated three times. Error bars represent the standard deviation.

Primers used for RT-PCR are listed as below:

Bradi1g24880.1:

5’-TTGGTCTCTCTTCATGGCAA-3’;

5’-TTGCACATTGAAATCGAAGTG-3’;

Bradi2g55060.1:

5’-ACAATCTACGGCGCGCTCATCATA-3’;

5’-GTGCGCATCAATCTCCACATCGTT-3’;

Bradi4g44810.1:

5’-TCGGCGAGTGGTGGGACA-3’;

5’-TCGGAGACGTTGGGTGCTGC-3’;

Bradi1g13160.1:

5’- CGAGAGGAAACACTCAAGAAGGCTGA-3’;

5’-CTATGCTCTACGGCAAGGTACGGTTT-3’;

*Tubulin:*

5’-GTTCACCGTGTACCCATCTC-3’;

5’-AGAAGGACAGCCACATCAGT-3’.
